# Cereal Biomass Valorization for Functional Food Ingredients: Fractionation and Circular Biorefinery Strategies

**DOI:** 10.1111/1541-4337.70681

**Published:** 2026-10-27

**Authors:** Rahul Biswas, Marjo Pöysä, Samson Adeoye Oyeyinka, Abayneh Getachew Demesa, Savindi Wanninayake, Samuel Perez‐Vega

**Affiliations:** ^1^ Department of Separation Science, LUT School of Engineering Sciences Lappeenranta–Lahti University of Technology LUT Kouvola Finland; ^2^ Systemic Food Processing (SFP) Research Group, Kouvola Unit Lappeenranta–Lahti University of Technology LUT Kouvola Finland; ^3^ Centre of Excellence in Agri‐Food Technologies, National Centre for Food Manufacturing University of Lincoln Holbeach UK; ^4^ Centre for Innovative Food Research (CIFR), Innovation Research Group, Department of Biotechnology and Food Technology, Faculty of Science University of Johannesburg Johannesburg Gauteng South Africa

**Keywords:** biopolymer recovery, cereal side streams, green separation technologies, structure–function relationships, zero‐waste food systems

## Abstract

Food processing must transition toward resource‐efficient systems that operate within planetary boundaries. Cereals, as climate‐resilient staples, offer strong potential to valorize nutrient‐rich fractions. Zero‐waste biorefinery concepts and green fractionation technologies, integrated with structure–function principles, enable the conversion of whole‐grain biomass into functional ingredients for food applications. This review systematically evaluates recent advances in fractionation and separation technologies, including nonthermal physical and biochemical approaches. A Preferred Reporting Items for Systematic reviews and Meta‐Analyses (PRISMA)‐guided analysis of peer‐reviewed articles (2020–2025) was conducted to assess extraction strategies, functional performance, and sustainability‐related aspects. Mechanistic insights into recovered biopolymers are discussed to design process–functional relationships, with an emphasis on fractionation strategies such as synergistic recovery, fraction precision, and yield optimization relative to process complexity. The analysis shows that 46.75% of studies focused on common grains, whereas 53.25% utilized side streams (42.86% bran) for the recovery of proteins (32.47%), fibers (55.84%), lipids (10.39%), and phytochemicals (89.61%). Although hybrid techniques enhanced selectivity, multi‐fraction recovery, and the preservation of biopolymer functionality, linear pathways dominated (70.13%), particularly in ultrasound‐assisted methods (57.14%). Few studies investigated whole‐system biorefinery approaches (14.29%), sustainability assessments (<5%), or pilot‐scale validation (10.39%). Findings reveal critical opportunities for integrating life cycle assessment, techno‐economic analysis, and digital modeling to facilitate scale‐up and industrial translation. Furthermore, underutilized crops and industrial residues show promise for circular valorization. The review highlights the potential of sustainable biorefineries to reduce waste, enhance resource efficiency, and support circular‐driven food innovation.

**Abbreviations**
ABTS: 2,2′‐azino‐bis(3‐ethylbenzothiazoline‐6‐sulfonic acid) radical scavenging assay
AI: artificial intelligence
ANNs: artificial neural networks
ATR: attenuated total reflectance
BSG: brewer's spent grain
CAPEX: capital expenditures
CE: circular economy
CP: cold plasma
DES: deep eutectic solvents
DPPH: 2,2‐diphenyl‐1‐picrylhydrazyl radical scavenging assay
EFSA: European Food Safety Authority
EH: enzymatic hydrolysis
EU: European Union
F: fermentation
FIs: functional ingredients
FRAP: ferric reducing antioxidant power assay
FTIR: Fourier‐transform infrared spectroscopy
GHG: greenhouse gas
GI: glycemic index
GRAS: generally recognized as safe
HPH: high‐pressure homogenization
LCA: life cycle assessment
M: microwave
MIR: mid‐infrared spectroscopy
NIR: near‐infrared spectroscopy
OH: ohmic heating
OHC: oil‐holding capacity
OPEX: operating expenses
ORAC: oxygen radical absorbance capacity
PBs: planetary boundaries
PCA: principal component analysis
PEF: pulsed electric field
PLS‐R: partial least‐squares regression
PRISMA: Preferred Reporting Items for Systematic reviews and Meta‐Analyses
PWE: pressurized water/fluid extraction
SCFAs: short‐chain fatty acids
SDGs: Sustainable Development Goals
SFE: supercritical fluid/CO_2_ extraction
SFP: sustainable food processing
SWE: subcritical water/fluid extraction
TEA: techno‐economic analysis
UDA: unsupervised domain adaptation
US: ultrasound
WHC: water‐holding capacity

## 1 Introduction

A growing global population, alongside the adverse effects of climate change and ecosystem degradation, has placed immense pressure on global food systems to deliver adequate, nutritious, and accessible food. In response, the industrial food processing sector has expanded considerably to meet rising demand. This expansion is, however, not without consequence, and it is raising concerns not only about food security but also about the long‐term stability of the biosphere (Poppy and Baverstock 2019). Critically, the food sector is a major contributor to greenhouse gas (GHG) emissions, excessive energy and freshwater use, and nutrient losses. These factors collectively intensify environmental impacts that directly transgress key planetary boundaries (PBs) (Perez‐Vega 2022). From a broad perspective, the PB framework can extend life cycle assessment (LCA) by establishing a safe operating space for food systems (Steffen et al. 2015) and guiding measurable actions on key control variables, such as limiting GHG emissions, reducing nitrogen and phosphorus use, capping freshwater consumption, keeping cropland below specific thresholds, and curbing biodiversity loss to ensure long‐term sustainability (Willett et al. 2019).

Sustainable food systems aim to ensure the simultaneous production of nutritious food worldwide and minimize environmental impact while securing resources for future generations (Brundtland 1987). This also supports synergies across multiple Sustainable Development Goals (SDGs) and transforms food production toward resilience and circularity (Campbell et al. 2018). Sustainable food systems can thus help drive progress toward meeting SDGs related to climate action (13), sustainable consumption and production (12), food security (2), good health and well‐being (3), water sustainability (6), life on land (15), poverty reduction (1), quality education (4), gender equality (5), and clean energy (7). Despite notable technological advancements, the current industrial food processing paradigm has historically emphasized yield, convenience, and shelf life over nutritional quality and sustainability (Augustin et al. 2016). This creates a systemic imbalance loop: increased industrial processing exacerbates ecological harm and fails to adequately mitigate global nutritional deficiencies.

By considering the food system within SDGs and PB frameworks, it becomes evident that sustainable food processing (SFP) strategies need to be redesigned to align with industrial practices to ensure environmental, ecological, and nutritional security goals (Donner et al. 2024; Galanakis 2022; Lindgren et al. 2018). Moreover, as SFP deals with a critical leverage point for successful transition to planetary sustainability, it is crucial to select suitable feedstocks. In this respect, low‐impact and nutrient‐dense food matrices represent a key leverage point for SFP (Perez‐Vega 2022), and cereals, in particular, can play a pivotal role in the transition of the food system.

Cereals offer a strong case for greater consideration due to their widespread cultivation, climate resilience, minimal input requirements, nutritional importance, and potential for sustainable production (Gazza and Nocente 2023; VYR 2014). When processed using sustainable methods that preserve bran and germ layers, cereals not only reduce the environmental impact but can also enhance the health‐promoting potential of food products (Tse and Reaney 2025). These benefits largely result from the retention of bioactive components, including dietary fiber, β‐glucans, arabinoxylans, phenolic acids, carotenoids, tocopherols, alkylresorcinols, phytosterols, and essential micronutrients, which are associated with improved antioxidant activity, glycemic control, cholesterol level management, and gut health (Mingfang et al. 2025; Tian et al. 2020; Wu et al. 2022). Despite their value, current industrial processes often discard nutrient‐dense fractions such as bran and germ layers, which reduces the functional food potential of many feedstocks and generates high volumes of underutilized by‐products and waste (Grasso 2020; Hassan et al. 2021; Saleh et al. 2019). Food production and processing are currently responsible for around 29.7% (5.5 Gt CO_2_‐eq) of total global GHG emissions, with processing and post‐harvest activities accounting for nearly one‐third of this share. Additionally, food losses and waste account for 8%–10% of total emissions, exposing systemic inefficiencies across the food system (FAO 2023).

Although cereals are globally important staple foods and contain many valuable components, industrial cereal processing, using techniques such as conventional flaking, air classification, and milling, generally focuses on extracting refined endosperm. As a result, the bioactive potential of the outer cereal layers remains underutilized due to their complex matrix and the resulting processing constraints (Arzami et al. 2022; Mao et al. 2022). For instance, the soft kernel texture of oat might limit processing due to the higher lipid content (typically 5%–9%) compared with other cereals, such as wheat, barley, maize, and rye (2%–3%). This higher lipid level increases susceptibility to oxidation, even though the oats are used whole as grains for flaking and hydrothermal treatment (Ames and Rhymer 2003; Gates et al. 2008; Sammalisto et al. 2022). Barley and rye are mainly processed for malt and bread production and are seldom valorized for use of their functionally‐rich bran (Galanakis 2022; Heiniö et al. 2016; Jaksics et al. 2022). Historically, bran was used for low‐value animal feed, but bran from oat and barley is now recognized as a rich source of nutrients and bioactive compounds for functional ingredients (FIs) (Bautista‐Expósito et al. 2020; Hassan et al. 2025; Li et al. 2021; Liu et al. 2021; Martín‐Diana et al. 2021; NEMES et al. 2024). The most significant volumes of cereal by‐products arise from industrial wheat milling, where the separation of endosperm for refined flour production generates wheat bran containing the pericarp, testa, and aleurone layers. This fraction accounts for approximately 20%–25% of the grain's weight, and about 150 million tons of bran are produced worldwide (Bautista‐Expósito et al. 2020; Cherif et al. 2020; Martín‐Diana et al. 2021). Along with whole grains, which account for approximately 39% of food production waste, these by‐products constitute significant opportunities for fractionation and valorization in functional food applications (Cherif et al. 2020; Martín‐Diana et al. 2021).

Sustainability and process circularity are key concepts for the SDGs in many industrial and social sectors and are fundamental to achieving near‐zero food waste (Guerrini et al. 2020; Yilmaz‐Turan et al. 2020). Initiatives such as the European Circular Economy Action Plan and the United Nations 2030 Agenda aim to minimize post‐harvest losses and valorize by‐products by recovering valuable compounds from food processing waste and reintroducing them as raw materials into the economy. The need has led to extensive research on extraction and separation methods, as traditional extraction methods are often expensive, time‐consuming, and unsustainable due to excessive energy and solvent use (Guerrini et al. 2020). Safer, more efficient, and sometimes more economical green alternatives have been developed (Guerrini et al. 2020; Yilmaz‐Turan et al. 2020). Additionally, research is being undertaken to make greater use of hitherto underexploited crops. For example, studies on highland barley processing have been initiated to provide an empirical basis for developing novel functional foods and health products (Cao et al. 2023). The global functional food sector was valued at $382.51 billion in 2019 and is anticipated to expand rapidly due to rising consumer interest in nutritious and well‐balanced diets and the consequent demand for functional foods and nutraceuticals (Martín‐Diana et al. 2021). According to the European Food Safety Authority (EFSA), nutraceuticals are concentrated forms of nutrients or other substances that have physiological effects and are a growing market segment occupying the space between food and medicine (Martín‐Diana et al. 2021). Agri‐foods, which encompass a wide range of products from fresh produce to processed foods, are central to meeting global nutritional requirements (Shan et al. 2025). This highlights the potential of sustainable cereal feedstock transformation through zero‐waste biorefinery principles and circular economy (CE) models to recover and reintegrate functional components into food applications.

Recent advances in green and clean fractionation technologies, such as ultrasound (US), microwave (M), pulsed electric field (PEF), ohmic heating (OH), cold plasma (CP), enzymatic hydrolysis (EH), fermentation (F), membrane separation, pressurized water extraction (PWE), sub‐ (SWE), and supercritical fluid extraction (SFE), have opened sustainable pathways for the valorization of whole grains and bran. These techniques can reduce solvent use, improve resource efficiency, and help preserve heat‐sensitive nutrients and bioactive compounds (Koubaa et al. 2016; Nayak et al. 2024; Perez‐Vega et al. 2023). Most studies, however, remain limited to isolated unit operations or single compound recovery, and very few investigations perform multiple operations or comprehensive ingredient extraction (Sun et al. 2024). Moreover, many still lack integration into system‐level CE strategies (Aït‐Kaddour et al. 2024). Consequently, although individual green fractionation technologies have demonstrated potential for improving recovery efficiency and preserving functional compounds, their integration into multi‐fraction, zero‐waste cereal biorefineries remains insufficiently explored.

Recent literature has addressed various aspects of cereal biomass valorization, yet the scope remains dispersed across feedstocks, processing technologies, target compounds, and application domains. Comprehensive studies of cereal waste and by‐product valorization have documented a broad range of physical, chemical, and biological approaches for converting cereal residues into food, feed, biomaterials, biofuels, and other value‐added products, with an emphasis on emerging applications and sustainability considerations (Bamigbade et al. 2025; Ravichandran and Dixit 2025). F‐based valorization has also been examined in detail, particularly for producing proteins, fibers, phenolics, prebiotic oligosaccharides, and reducing antinutritional factors, alongside emerging considerations of artificial intelligence (AI), scalability, and economic feasibility (Adil et al. 2026). Other reviews have adopted a more focused, feedstock‐ or technology‐specific approach, including fractionation of maize and rice (Sukop et al. 2026), value addition of barley bran and husk (Hu and Gilbert 2025), pretreatment and lignocellulosic conversion of cereal residues (Ale et al. 2025), and extraction and purification of plant proteins (Akyüz et al. 2024). Some previous reviews have also provided detailed insights into specific bioactive classes or processing technologies, such as phenolic interactions, CP, or individual green extraction strategies, but have not considered the whole fractionation of cereal biomass within an integrated, food‐oriented biorefinery framework (Jeevarathinam et al. 2026; Rocchetti et al. 2022). These studies demonstrate substantial progress in cereal valorization; however, they primarily emphasize individual feedstocks, compound classes, technologies, pretreatment routes, or downstream applications rather than integrating these elements into a unified cereal biorefinery system. Table 1 compares the scope and major focus of recent relevant reviews with the present study, highlighting the broader integration of fractionation, multi‐ingredient recovery, functionality, sustainability, and scale‐up considerations in the present review.

**TABLE 1:** Comparison of recent and existing literature frameworks with the scope of the present review framework.

Review study	Main scope	Fractionation	Multi‐ingredient recovery	Sustainability	Scale‐up	Main distinction from present review
Bamigbade et al. (2025)	Cereal waste, by‐products	✓	Limited	✓	Limited	Broad waste valorization
Ravichandran and Dixit (2025)	Cereal, millet waste	✓	Limited	✓	Limited	Broad value‐added products
Adil et al. (2026)	Fermentation	Limited	✓	✓	✓	Fermentation‐centered
Sukop et al. (2026)	Maize, rice	✓	Limited	✓	Limited	Two cereals, fraction structure–function
Hu and Gilbert (2025)	Barley bran, husk	Limited	Limited	✓	Limited	Single cereal, by‐products
Ale et al. (2025)	Cereal lignocellulosic waste	✓	Limited	✓	✓	Pretreatment, lignocellulose
Akyüz et al. (2024)	Plant proteins	✓	No	✓	Limited	Protein‐centered
Present review	Multi‐cereal biomass	✓	✓	✓	✓	Integrated cereal biorefinery framework

*Note*: ✓ indicates that the review explicitly addresses the corresponding aspect as a substantive component of its scope; Limited indicates that the aspect is mentioned or discussed but is not a primary focus; and No indicates that the aspect is not substantially addressed.

Despite these contributions, an important gap remains in systematically connecting cereal biomass fractionation with multi‐fraction ingredient recovery, functional performance, process integration, sustainability, and scale‐up readiness. Most previous studies have primarily investigated broad waste valorization, individual cereal residues, specific fractionation or extraction technologies, processing pathways, lignocellulosic conversion, or individual ingredient classes. Consequently, limited details are available on the comparative performance of different fractionation strategies across diverse cereal matrices when ingredient recovery yield, selectivity, functional properties, process complexity, and potential for sequential recovery are considered together. In particular, the difference between linear and integrated fractionation pathways has received limited quantitative consideration, although this distinction is important for developing zero‐waste food biorefineries capable of recovering multiple valuable fractions from the same biomass stream. Furthermore, sustainability assessment remains insufficiently connected to fractionation performance, with limited integration of LCA considerations, CE implementation, PB perspectives, techno‐economic feasibility, and pilot‐scale validation.

A system‐based, circular approach to cereal processing, grounded in LCA thinking and CE principles, is key to advancing sustainable transformation. In such a framework, each unit operation is evaluated against three criteria: ingredient recovery efficiency; environmental pressure relative to PB thresholds; and alignment with system circularity. These evaluation metrics facilitate process optimization and address the trade‐offs among yield, sustainability, and system circularity. In the context of cereal processing, this system‐based, circular approach involves valorizing multiple grain fractions into high‐value additions while adhering to SDGs and the limits of the PB framework. Such a strategy can enable a regenerative agricultural cycle and catalyze the emergence of nutrition‐sensitive industry clusters, especially in regions with high levels of cereal production and processing. Furthermore, the synergy of sustainability metrics and nutritional targets early in technology design can drive scalable, industry‐ready solutions. To bridge current gaps, a multifractional, integrated biorefinery model is needed to enable full‐spectrum grain utilization while reducing waste and enhancing nutritional quality. This framework is illustrated in Figure 1 as a conceptual biorefinery model for advancing cereal processing toward regenerative food systems and planetary health.

**FIGURE 1:**
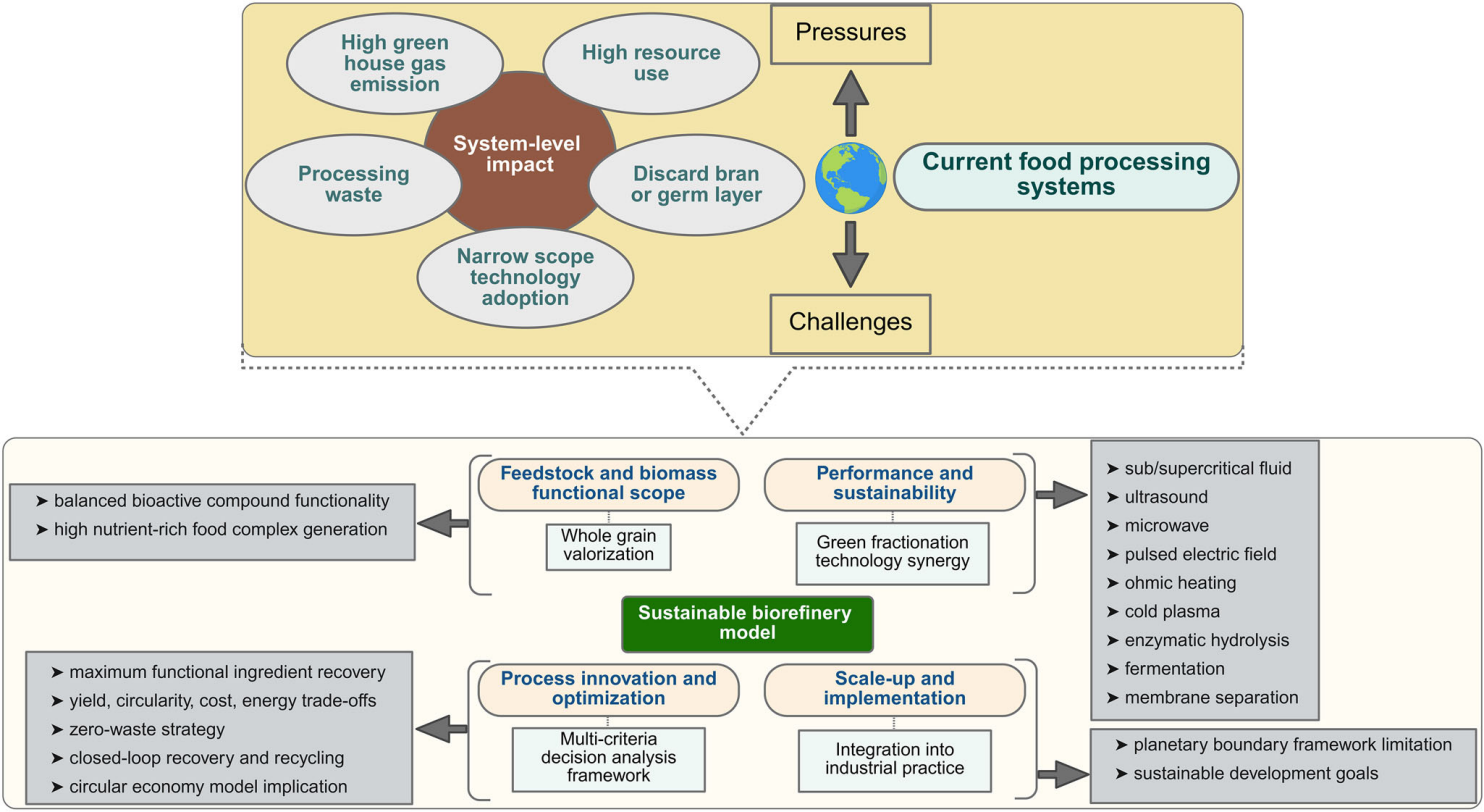
Systematic approach of sustainable food processing model.

In contrast, the present review adopts a system‐oriented perspective that integrates (i) quantitative analysis of ingredient recovery yields across cereal matrices, (ii) structure–function relationships determining functional performance, and (iii) sustainability metrics aligned with LCA considerations, CE concepts, and PB frameworks. The review compares linear and integrated fractionation strategies within a zero‐waste biorefinery concept, considering yield, process complexity, scalability, and industrial applicability. Instead of focusing on a single target compound or processing technique, particular emphasis is given to recovering multiple ingredient classes, such as proteins, fibers and prebiotic fractions, lipids, and phytochemicals. In addition, the review evaluates the current level of pilot‐scale validation and identifies opportunities for techno‐economic analysis (TEA), process modeling, digital tools, and multi‐criteria optimization to balance ingredient recovery, functional performance, environmental impact, and economic feasibility. This integrated approach enables the development of a structured roadmap for industrial‐scale sustainable cereal biorefineries, identifying opportunities for multi‐fraction recovery, functional optimization, and circular food system innovation.

In this context, linear (single‐step) fractionation refers to processes that isolate a single primary target component through a single dominant extraction or separation operation, whereas integrated (multi‐step) fractionation involves sequential or combined processing stages to recover multiple ingredient fractions from the same biomass stream. Throughout the review, ingredient yields are grouped into functional categories: protein isolates or concentrates; fiber or prebiotic fractions, including dietary fibers such as β‐glucans, arabinoxylans, and soluble oligosaccharides; and lipids or phytochemicals, such as phenolic acids, flavonoids, and ferulic acids. The systematic literature review then evaluates comparative ingredient yields, functional performance, extraction efficiency, process integration, sustainability considerations, and the current level of scale‐up and industrial readiness across diverse cereal matrices. Innovation pathways through advanced process modeling, TEA, and multi‐criteria optimization are also identified to support the development of a boundary‐safe roadmap for industrial‐scale integration.

## 2 Literature Review Methodology

This study employed a structured approach to systematically identify, select, and evaluate relevant literature published within the defined study period on advanced cereal fractionation technologies for producing functional food ingredients. The methodology was performed in accordance with the PRISMA (Preferred Reporting Items for Systematic reviews and Meta‐Analyses) guidelines (Haddaway et al. 2022).

### 2.1 Database Search Strategy

A comprehensive search was conducted on reputable research databases, including Web of Science, Scopus, and PubMed. The search strategy was designed to capture studies on the green fractionation of cereal grains and side streams, with a focus on multi‐ingredient recovery and sustainability. Keywords were grouped into three main categories: (i) feedstock type, (ii) processing and extraction technologies, and (iii) target ingredients and functionality. Boolean operators (AND/OR) were applied to combine keywords and ensure comprehensive coverage. The final search strings were adapted to each database's syntax.

The keywords were combined using Boolean operators to apply the following search string:

((fraction* OR “emerging process*” OR separation* OR extract* OR biorefinery*)

AND

(oat* OR barley* OR rye OR wheat* OR maize)

AND

(milling* OR flake OR supercritical OR subcritical OR SFE OR ultrasound OR sonication OR microwave OR “pulsed electric field*” OR PEF OR “ohmic heating” OR “high hydrostatic pressure*” OR HHP OR “cold plasma” OR hydrolysis OR “enzyme‐assisted” OR fermentation)

AND

(“functional food” OR ingredient* OR superfood* OR protein* OR fibre* OR fiber* OR “dietary fibre” OR starch OR prebiotic* OR carbohydrate OR phytochemical* OR bioactive* OR antioxidant* OR phenolic* OR tocopherol))

OR

((“cereal biorefinery*” OR “cereal process*” OR “cereal fraction*” OR “grain biorefinery*” OR “grain process*” OR “grain fraction*”)

AND

(“sustainable process technology” OR “sustainable food system” OR “planetary boundary” OR “environmental impact” OR sustainable* OR sustainability* OR “life cycle assessment” OR LCA OR “techno‐economic” OR TEA OR “energy use” OR “water footprint” OR “circular economy” OR “zero waste” OR “valorization” OR optimization OR “multi‐criteria decision‐analysis” OR pareto OR CAPEX OR OPEX OR model* OR pilot* OR “mass‐balance” OR closure OR regulatory* OR “novel food*” OR “machine learning” OR AI OR “digital twin”)).

The literature search was filtered by subject area, publication year, language, and article type from databases and imported into the Rayyan literature review tool for duplicate removal and screening using predefined inclusion and exclusion criteria and quality considerations by two reviewers. Although the primary search strategy focused on the predefined cereal terms presented above, relevant studies involving other cereal grains were retained during screening when they met the predefined eligibility criteria and were considered relevant to the scope of cereal fractionation and valorization. The search strategy was determined for its appropriateness to the review objectives and its ability to identify relevant studies by cross‐checking the retrieved records against relevant publications identified during the preliminary literature assessment. During the initial screening, the retrieved records were examined to determine whether the search terms captured relevant studies across the predefined cereal feedstocks, processing technologies, and target ingredients. Study relevance was assessed during title/abstract and full‐text screening in accordance with the eligibility criteria. The search terms were refined where necessary to improve the retrieval of studies relevant to the review objectives. Publications were retained only when their relevance could be established according to the predefined eligibility criteria and sufficient information was available for the intended qualitative or quantitative synthesis.

### 2.2 Eligibility Criteria and Selection of Studies

Articles were selected for the systematic literature review according to predefined eligibility criteria. The inclusion criteria were (i) original peer‐reviewed experimental studies published between 2020 and June 18, 2025; (ii) studies written in English with full text available; (iii) studies investigating cereal grains, including oat, barley, rye, wheat, maize, or cereal processing side‐streams such as bran, germ, and brewer's spent grain (BSG); (iv) studies investigating green fractionation, extraction, or valorization technologies, including US, M, SFE, SWE, PWE, PEF, OH, CP, EH, F, or combinations of these technologies; and (v) studies reporting quantitative outcomes related to the recovery of target components, such as proteins, dietary fiber/prebiotics, lipids, phytochemicals, or other bioactive compounds, or reporting environmental or techno‐economic performance.

Studies were excluded if they were (i) reviews, meta‐analyses, perspectives, editorials, or patents; (ii) conference abstracts or other publications without sufficient full‐text experimental information; (iii) non‐English‐language publications; (iv) publications for which the full text was unavailable; (v) studies unrelated to cereal fractionation, extraction, or valorization; (vi) studies involving non‐target raw materials or products; (vii) studies focused exclusively on conventional processing without a relevant green or emerging fractionation/valorization approach; or (viii) studies that did not provide sufficient quantitative information on the recovery or performance of the investigated process.

The retrieved records were initially screened based on titles and abstracts after duplicate removal, and potentially relevant studies were subsequently subjected to full‐text screening against the predefined eligibility criteria. Particular attention was given to the relevance of the feedstock, processing technology, target product, and availability of quantitative outcomes required for comparative analysis. Disagreements or uncertainties identified during the screening and eligibility process were resolved through discussion and consensus between the reviewers. The comprehensive literature search retrieved 3530 records from the selected databases, and 77 articles were included in this study, as illustrated in Figure 2.

**FIGURE 2:**
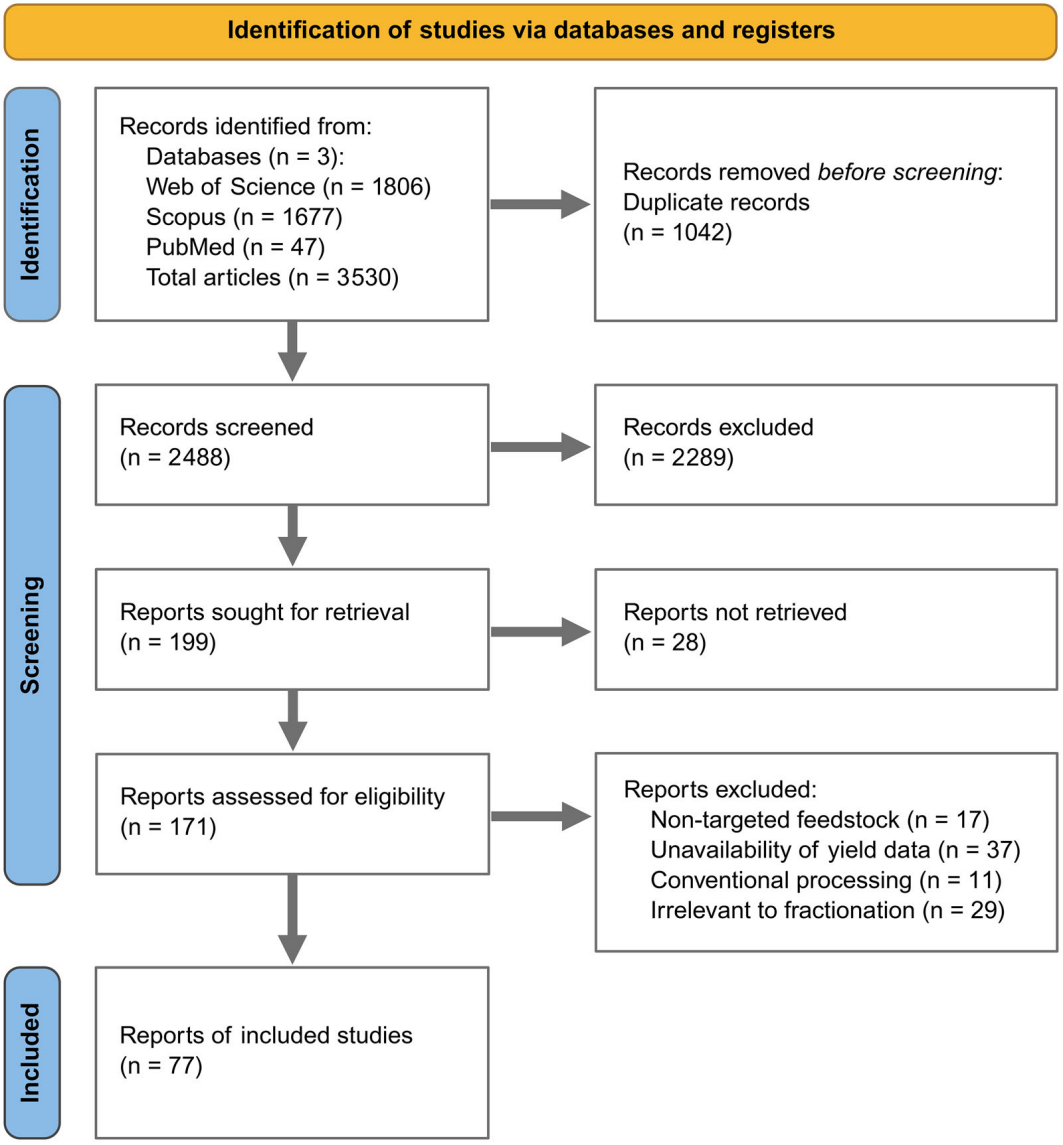
PRISMA flow diagram of the systematic review.

### 2.3 Study Quality Assessment and Data Quantification

The methodological quality and suitability of the identified studies were considered during the screening and full‐text evaluation by two reviewers using predefined quality considerations. The assessment focused on (i) relevance of the study to the review objectives; (ii) adequacy of the experimental or processing information; (iii) availability of quantitative outcomes; (iv) clarity of the reported processing conditions; and (v) suitability of the reported data for comparative analysis. Studies that did not provide sufficient information to establish their relevance, process performance, or quantitative outcomes were excluded from the final synthesis. Thus, the assessment was used to determine the evidential suitability of the included studies for the intended qualitative and quantitative synthesis and to assess whether sufficient and relevant information was available for comparison of cereal fractionation and valorization technologies, rather than to assign a formal standardized methodological quality score.

Yield data for protein, fiber, prebiotic, lipid, and phytochemical recovery were collected from the selected articles. Because the included studies reported recovery values using different units and experimental bases, the reported values were standardized, where possible, to a common basis of percentage of the initial dry material (% w/w) to facilitate comparison. The compiled data were grouped according to processing method and biomass type. Statistical descriptors, including mean, median, standard deviation (SD), minimum‐to‐maximum range, and interquartile range (IQR), were used where sufficient comparable data were available to provide a comparative assessment of recovery effectiveness and process performance across different technologies and feedstocks.

### 2.4 Limitations of the Literature Search Strategy

Although a comprehensive and structured search strategy was applied, several limitations should be considered when interpreting the findings. First, the literature search was restricted to Web of Science, Scopus, and PubMed; therefore, relevant studies indexed in other databases may not have been captured. Second, the search was limited to English‐language publications and studies published between 2020 and June 18, 2025, which may have excluded relevant evidence published in other languages or outside the defined period. Third, the identification of studies depended on the predefined search terms and Boolean combinations; consequently, studies using terminology not represented in the search strategy may have been overlooked. In addition, the exclusion of studies without sufficient quantitative information may have reduced the number of studies available for direct comparison. Finally, substantial heterogeneity among the included studies in terms of cereal matrix, pretreatment, processing conditions, extraction methodology, yield definitions, and reporting practices limits direct comparison and statistical aggregation across all technologies. These limitations were considered when interpreting the quantitative comparisons and overall conclusions of the review.

## 3 Trends in Cereal Biomass Fractionation

This systematic review synthesized results from peer‐reviewed studies and consolidated perspectives on cereals into nutrient‐rich ingredient extraction targets, technological feasibility, and sustainability evaluations. Figure 3 illustrates key dimensions and findings from those studies, highlighting the field's ongoing early transition toward systemic analysis.

**FIGURE 3:**
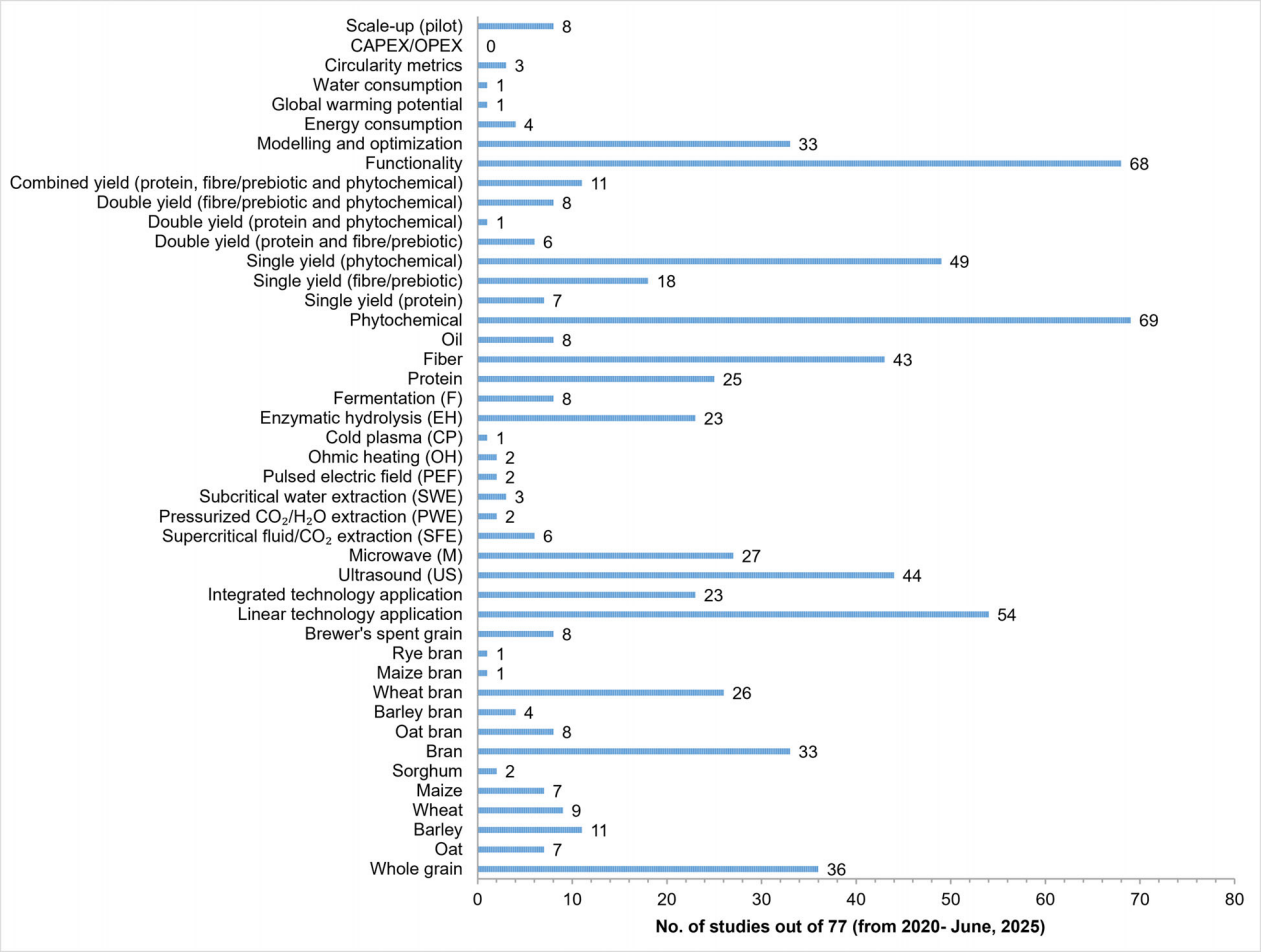
Systematic literature reviews outcomes of cereal‐based ingredients extraction.

According to this systematic analysis, most studies focused on cereals such as wheat, barley, oat, maize, and sorghum, which together accounted for nearly 46.75% of the studies reviewed. Value‐added ingredients from bran streams, especially wheat bran, were the most common feedstocks investigated (33.77%), reflecting their abundance in the food biorefinery industry and their recognized potential as protein‐ and fiber‐rich substrates. However, relatively few studies explored rye and maize bran (1.30%) or sorghum (2.60%), suggesting underutilization of alternative grains that may become more important under critical scenarios. BSG was studied in 10.39% of studies, indicating growing recognition of brewing residues as promising substrates for circular valorization.

The most commonly adopted processing technologies were US and M applications (57.14% and 35.06%, respectively), often used in linear extraction configurations. Methods such as SFE, SWE, or PWE, PEF, OH, and CP were investigated in significantly fewer studies (1.30%–7.79%). The prevalence of linear extraction approaches (70.13%) indicates a yield‐centric research paradigm that prioritizes the recovery of certain phytochemicals (63.64%) over integrated valorization. The limited use of whole‐system biorefinery concepts was evident in 14.29% of the research, demonstrating the simultaneous recovery of proteins, fibers, and phytochemicals. Almost 88.31% of the studies focused on the functionality or bioactivity of recovered components, whereas systemic sustainability assessments were rarely considered. For example, only 5.19% of studies quantified energy consumption; 1.30%–3.90% addressed water use, circularity, or GHG emissions; and none provided data on capital expenditures (CAPEX) or operating expenses (OPEX). LCA was performed in only one study, and techno‐economic insights were not fully included, despite their importance for PB‐aligned process evaluation (Perez‐Vega et al. 2023). Nonetheless, the study optimized variables for cradle‐to‐gate LCA without quantifying bioactives, considering scale‐up, or performing an economic evaluation. Although process optimization was reported in almost 42.86% of the literature, a modeling approach was used in only a few studies. Only 10.39% of studies attempted pilot‐scale demonstration, with most work confined to laboratory trials (≤400 g batches).

### 3.1 Cereal Fractionation Technologies: Linear vs. Integrated

This systematic literature review analyzed recent advancements in cereal fractionation technologies, with particular emphasis on the extraction yields of key FIs, namely, protein, dietary fiber/prebiotics, lipids, and phytochemicals from various studied grains. The studies were categorized by processing approaches, such as linear versus integrated biorefinery systems. Table 2 provides a study‐level comparison of the reviewed fractionation strategies through the association of cereal biomass, target ingredient, processing pathway, extraction conditions, recovery performance, functionality, and technological readiness. These assessments evaluate technological suitability, recovery efficiency, product purity, functional properties, processing conditions, and opportunities for fractionation. Integrated fractionation procedures coordinate the simultaneous recovery of multiple potential fractions from a single feedstock, thereby significantly enhancing whole‐mass utilization. The single‐product hydrocolloid extraction into multi‐product separations transitions captures earlier discarded proteins and other compounds, although it typically raises operational complexity and costs (Liu, Gao, et al. 2024). The most comprehensive pool of functional proteins is produced when physical and enzymatic treatments are combined, which considerably exceeds the results of isolated mechanical stages (Timira et al. 2024). However, greater recovery through integrated processing does not necessarily translate into greater industrial feasibility, because additional unit operations can increase process complexity, CAPEX, OPEX, and downstream separation requirements. From an industrial‐readiness perspective, dry and mechanical fractionation generally have advantages due to their established equipment, relatively simple operation, and lower water demand, although they may offer lower selectivity or purity. In contrast, intensified approaches such as US, M, OH, SFE, enzymatic, and hybrid processing can improve selective recovery or functionality but require greater control of operating conditions and, in several cases, specialized equipment or longer processing times. As a result, their industrial potential depends on whether the additional processing requirements are justified by the value of the recovered products.

**TABLE 2:** Summary of reviewed cereal fractionation studies and semi‐quantitative assessment.

Reference	Biomass	Target ingredients	Fractionation mode	Processing pathway	Extraction technology and conditions	Recovery yield, purity, and functional properties	Highlights	Industrial readiness and opportunities	Score^a^
Gaber et al. (2025)	Oat whole grain, active, and standardized commercial cultivars	Protein concentrates and texturized protein	Dry fractionation (air clasification) → structural texturization	Multi‐step (physical)	SFE defatting (350 bar, 45°C, 200 mL/min) → pin milling (17,800 rpm) + air classification (20,000 rpm, 65 m^3^/h, 6 kg/h → extrusion	Yield: ∼15%–20% Protein: 44.2% standardized dry fraction; 55.8% active dry fraction; 53.9% wet‐milled Dry fractionate: higher EAI (44.2–48.0 m^2^/g) and WSI (7.5%–9.8%); wet milling: higher ESI (31.4 min) and WHC (1.7 g/g)—cutting force ∼140 N; concentrates: potential for low‐ and high‐moisture extrusion	First oat‐only protein biorefinery chain	Pilot scale SFE Optimize extrusion parameters; scale SFE and extrusion for industry	4.3
Sargautis and Kince (2023)	Oat commercial flakes	Oat protein concentrate	EH → ionic shift	Multi‐step (enzymatic‐physical)	EH (S/L 1:10 w/w; α‐amylase: 75–80°C, 30 min) + NSPH (60°C, 20 min → 80°C, 20 min) + ionic shift aggregation (0.1 M NaCl, 75°C, 4 h)	Yield: 44.8% (α‐amylase + NaCl); 35.9% without NaCl; 33.1% with α‐amylase + xylanase + NaCl Protein: 84.20% (α‐amylase + NaCl); maximum 86.46% with α‐amylase + xylanase + NaCl WHC up to 3.0 g/g; OHC up to 2.1 g/g; solubility <7%; foamability <8%	Ionic shift enhances protein aggregation and recovery; high nutritional quality with essential amino acids (lysine‐limited)	Lab/bench scale Improve solubility and foamability of protein concentrate; test in functional foods and plant‐based products; scale enzymatic‐ionic process	4.0
Sargautiene et al. (2023)	Oat commercial flakes	Synbiotic formulation: β‐glucan + akkermansia muciniphila	Enzymatic precipitation → drying	Multi‐step (enzymatic‐physical‐biological)	EH (pH 4.5; α‐amylase: pH 7.0, 85°C) → ethanol precipitation (1:8, 30°C, 60 min) → 20 kDa ultrafiltration/diafiltration → drying (freeze‐dried or spray‐dried: 90°C exhaust) → fermentation	Yield: 80% β‐glucan: 90% Enhanced SCFAs production (propionic, acetic acids); both drying methods are suitable; freeze drying has a higher cost	First study integrating next‐gen probiotic with oat β‐glucan; functional SCFAs enhancement	Lab scale Validate synbiotic formulation in vivo; optimize cost‐effective drying for commercial applications	4.0
NEMES et al. (2024)	Oat commercial bran	Phenolic acids, lipid‐fatty acids	Acid pretreatment + SSF	Multi‐step (chemical–biological)	Acid pretreatment (3% H_2_SO_4_, 1:5 w/v, 20–25°C, 2 h) + SSF (*Aspergillus niger*, 1 × 10^6^ spores/g, 50% moisture, 30°C, 6 days) → solvent extraction	Yield increased: 4.66%–7.33% lipid; 2.52%–5.81% GAE TPC Unsaturated fatty acids: 80.29% DPPH inhibition 83.33	Time‐course profiling reveals dynamics of phenolic and lipid accumulation; acid pretreatment enhances accessibility	Lab scale Combine acid pretreatment and SSF for industrial‐scale valorization; monitor phenolic/lipid dynamics to maximize functional compound yield; evaluate cost‐effectiveness	4.0
Bai et al. (2022)	Oat commercial bran	Starch‐rich fraction; lipid‐, β‐glucan‐ and protein‐depleted fractions	M‐assisted chemical extraction after thermal treatment	Multi‐step (physical–chemical)	M‐assisted chemical extraction following thermal treatment (steaming 20 min; 800 W, 2 min; hot‐air drying 140°C, 15 min; defatting: petroleum ether, 1:5 w/v, 23°C, 4 h × 3; β‐glucan hydrolysis: 0.1 M acetate buffer, pH 5.2, 55°C, 30 min; deproteinization: 0.02 M carbonate buffer, pH 9.0, 45°C, 1 h × 2)	Yield: 67.80%–80.34% lipid; 77.66%–88.90% β‐glucan; 18.32%–19.41% to 7.62%–9.87% protein depletion β‐glucan: 1.05%–1.93%; protein: 7.62%–9.87%; starch: 46.91%–55.01% starch Lowest GI 66.11 and hydrolysis index (48.09) for steamed oat bran: protein removal increased solubility to 30%–40%	Thermal and chemical treatment improves starch digestibility; steaming preserves low GI Removal of lipids, β‐glucan, and protein increased starch digestibility, with inhibition hierarchy lipids > β‐glucan > protein	Lab scale; steaming is readily scalable Optimize thermal and chemical treatments to balance digestibility and β‐glucan retention; develop low‐GI functional foods; require sequential integration of solvent extraction and enzymatic fractionation	3.9
Radenkovs et al. (2021)	Oat, rye, wheat bran	FA and hydroxycinnamates	EH and SFE comparative	Multi‐step (enzymatic‐physical)	EH (pH 4.6, 44°C ± 1°C, 100 rpm, 48 h; S/L 1:10) ± SFE (150 bar, 50°C, 10% EtOH, 5.4 mL/min) + solid‐phase extraction	FA release: rye 0.37% by EH; wheat 0.26%; oat 0.034%; SFE gave up to 0.46% total FA from rye FA: 94% purity	EH is sustainable, scalable, and cost‐effective for FA recovery	Bench‐to‐pilot Industrial‐scale enzymatic hydrolysis for ferulic acid; integrate with SFE for sustainable multi‐product recovery; assess cost‐efficiency	4.2
Ibrahim et al. (2025)	Oat cultivars, whole grain	Phenolic‐rich bioactive extract	US‐assisted solvent extraction	Linear (physical)	US (optimized by RSM) (20 kHz, 500 W; 95% ethanol 1:20 w/v, 24 h, 25°C pre‐soaking; 35–55 min, 45–65°C, 70%–90% amplitude; optimum 55 min, 65°C, 90% amplitude) → filtration → evaporation	Yield: 9.60%–54.80%; model optimum 45.05%; extraction efficiency 83.75%, TPC: 9.68% GAE DPPH scavenging 10.52%–72.30%; high antioxidant potential	Cultivar‐specific optimization demonstrates scalable green extraction	Lab/bench scale Pilot‐scale validation; optimize for different cultivars	3.8
Ibrahim, Nadeem, Sultan, et al. (2024)	Oat cultivars, whole grain	Dietary fiber‐rich fraction: TDF, IDF, and SDF; phenolic‐rich fiber	Physical green fractionation	Linear (physical‐green)	US‐assisted extraction (20 kHz, 750 W, 10–30 min, 20–40°C, 60%–80% amplitude; 95% ethanol 1:10 w/v, 24 h, 25°C; aqueous S/L 1:20 w/v); optimized sonication power‐time, amplitude, and temperature	Yield: 60.05%–80.76% TDF; 56.15% IDF; 24.75% SDF TDF: 58.6%–83.20%; FA: 0.15%; residual fat: 0.98%–1.34% DPPH activity 29%–59%; water solubility 0.86–1.24 g/g; WHC 1.10–1.46 g/g; OHC 0.96–1.97 g/g	Sonication efficiently extracts fibers and polyphenols; cultivar‐dependent performance; supports nutraceutical applications	Lab/bench scale Test industrial scalability; validate functional and antioxidant performance in finished food products	4.1
Perez‐Vega et al. (2023)	Oat groat	Protein‐rich phase, oil‐rich phase, fiber‐rich phase	US‐assisted three‐phase partitioning (integrated product)	Linear (physical)	Milling → US‐assisted three‐phase partitioning (40 kHz, 500 W; optimized: 15 min, 4.74 wt% (NH_4_)_2_SO_4_, 50 vol% butanol) → phase separation	Yield: 4.5%–15.3% protein‐rich (optimized 15%); 5%–8.6% oil‐rich (optimized 8%); 66.3%–78.8% fiber‐rich (optimized 72%) Protein: 64.1%–78.7%; oleic acid: 38.10%–42.81%; linoleic acid: 25.51%–33.86% Elastic weak‐gel behavior (*G*′ > *G*″; tan δ <1); defatting increased *G*′, water absorption, and peak pasting viscosity	Allow simultaneous fractionation into protein, oil, and fiber with high mass recovery and strong biorefinery potential; environmental impact is dominated by butanol; solvent recycling is essential	Bench‐to‐early pilot Optimize industrial sustainability; implement solvent recycling to reduce environmental impact; quantify bioactives; perform economic evaluation; scale‐up needed	4.2
Ibrahim, Nadeem, Khalid, et al. (2024)	Oat cultivars, whole grain	TDF, SDF, IDF	Physical green fractionation	Linear (physical‐green)	US‐assisted extraction (20 kHz, 500 W; 95% ethanol 1:10 w/v, 24 h, 25°C; 10–30 min, 20–40°C, 60%–80% amplitude; model optimum: 25.08 min, 26.97°C, 69.47% amplitude); RSM optimization	Yield: 63.20%–79.82%; extraction efficiency ∼85% TDF: 43.75%–62.75%; IDF: 32.75%–42.25%; SDF: 11.0%–20.5% porous honeycomb structure; good thermal properties; relative crystallinity 47.54%; surface area 1.76 m^2^/g; pore volume 1.57 cc/g; thermal stability up to 240°C; potential bile‐acid adsorption	Sonication outperforms hot water, acidic, enzymatic, and alkali methods; excellent fiber structure	Lab/bench scale Benchmark against alternative fiber extraction methods; assess energy efficiency and industrial feasibility	4.2
Malenica et al. (2025)	Oat bran, industrial by‐product	Phenolic‐rich antioxidant extract	US‐induced cell disruption	Linear (physical)	US extraction (70% ethanol, 1:20 w/v; stirring 30 min; 400 W, 5–30 min, 20%–50% amplitude, <30°C; optimum for TPC/DPPH: 17.5 min, 20% amplitude; ABTS optimum: 17.5 min, 50% amplitude); gravity filtration	TPC: 0.119% GAE Antioxidant capacity up to 18.2 µM TE/g DW (ABTS) and 15.5 µM TE/g DW (DPPH)	Technology versatility across biomasses Shortened extraction time with high polyphenol yield and strong radical scavenging activity	Lab scale Conduct LCA; test continuous US; industrial validation	3.7
Li et al. (2024)	Oat bran	Free and bound phenolics, ferulic acid, flavonoids, water extractable AX	SSF enrichment	Linear (biological)	SSF (1:10 solid–liquid ratio, 2% fermentum, 37°C, 96 h)	Yield: 3.37‐fold free phenolics; 3.99‐fold bound phenolics; 6.44‐fold FA; 2.88 bound FA; 44.1% SDF and 4.51‐fold AX increased TPC: bound 0.23% GAE, free 0.11% GAE; flavonoids: bound 0.13% CE, free 0.12% CE; SDF: 5.36% Phytic acid reduction by 35%; increased DPPH (41.23%, 51.02%), ABTS (79.48%, 90.01%), –OH (51.41%, 60.49%), O_2_ (15.78%, 17.91%) radical scavenging, reducing power, anti‐lipid peroxidation	Fermentation releases bound phenolics and reduces antinutrients; improves nutritional/functional profile of oat bran	Lab scale Develop functional oat bran products with enhanced SDF, phenolics, and reduced antinutrients; scale SSF process; assess stability and bioavailability in final foods	4.1
Grgić et al. (2023)	Oat, barley bran	Bran modification (enzyme activity and phytochemical)	US modification	Linear (physical)	High‐intensity US (24 kHz, 400 W; 87–348 kJ/kg; 40–85°C) → freeze drying	Phytic acid reduced 17% (oat), 39% (barley); total phenolics reduced 27%–55%; antioxidant activity decreased 28%–48%; hydration properties increased 42%–59%	US reduces antinutrients while modifying functional properties; modified bran suitable for liquid and baked products	Bench/early pilot scale Apply US‐modified bran in liquid or baked formulations; optimize reduction of antinutrients while maintaining antioxidant activity; explore combination with other functional ingredients	3.8
Călinoiu and Vodnar (2020)	Oat, wheat bran	Phenolic‐rich extracts	Thermal + US‐assisted extraction	Linear (thermal‐physical)	US + thermal pretreatment (ultrasonic bath 40°C, 1 h; 80°C, 10 min) → centrifugation	Yield: 22.49% (wheat) and 25.84% (oat) increased TPC (wheat): 0.049% GAE, FA: 0.0031%; avenanthramide 2c (oat): 0.00063% DPPH inhibition 47.4% (wheat), 51.75% (oat)	Thermal pretreatment improves bioaccessibility; US enhances extraction efficiency	Lab/bench scale Combine thermal pretreatment and US for bran phenolics at commercial scale; investigate bioaccessibility and functional ingredient applications in bakery or cereal products	4.0
Milićević et al. (2021)	Oat, wheat bran	Total phenolic content	US‐assisted extraction	Linear (physical)	US‐assisted (1:20 w/v solid–liquid ratio, 70:30 ethanol–water ration; 25°C; 750 W, 20 kHz, 10 min) → solid–liquid separation	Wheat bran TPC: 0.23% GAE; oat bran TPC: 0.15% GAE	Peleg's model, applied to describe extraction kinetics, allows rapid comparison of US efficiency across brands	Lab/bench scale Apply Peleg's kinetic model for industrial US optimization; compare different cereal bran for large‐scale phenolic recovery	3.9
Anis et al. (2024)	Barley varieties	SDF and β‐glucan	US‐facilitated enzymatic release	Multi‐step (physical‐enzymatic)	US‐assisted enzymatic extraction (defatting: 80% ethanol, 1:5 w/v, 12 h; 130.5 W, 41.70°C, 17.5 min); RSM optimization	Yield: 5.29% Mixed β(1 → 3) and β(1 → 4) glycosidic linkages; nitrogen‐free extract: 73.68%–78.37%; fiber: ∼10%; protein: 7%–8.85%; residual fat: ∼1.22% WHC: 14.07 g/g; OHC: 6.99 g/g; foaming capacity: 126.17%; foaming stability: 96.26%; initial moisture desolvation endotherm at 50–59°C followed by a prominent melting/structural transition 130°C; highly thermostable up to 170–220°C; primary thermal pyrolytic decomposition occurred between 220°C and 450°C (30%–45% mass loss)	Provides cultivar‐sensitive process optimization for functional fiber extraction Optimized US + EH generated SDF/β‐glucan fractions with exceptional hydration and foaming capacity across cultivars	Lab scale Test industrial scalability and process robustness; further optimize functional traits across varieties	4.0
Chen et al. (2024)	Highland barley	Antioxidant activity of β‐glucan fractions	M + US‐assisted extraction	Multi‐step (physical‐green)	M pretreatment (80% ethanol, 1:10 w/v ration, 80°C, 4 h pretreatment; 480 W, 4 min) → US extraction (480 W, 20 min; holding 55°C, 2 h); RSM optimization	Yield: 25.67% DPPH scavenging Arabinose: 38.23%; xylose: 31.60% Purified fractions show concentration‐dependent antioxidant activity; low‐molecular weight fraction has higher antioxidant potential	Combined US‐M extraction improves β‐glucan yield and antioxidant quality; fraction‐dependent functional properties Hierarchy of scavenging activity: ABTS > –OH > superoxide > DPPH	Lab scale Explore formulation in food and cosmetic products; assess industrial‐scale M–US extraction	4.0
Liu, Wu, et al. (2024)	Highland barley	Phenolic compounds	US‐assisted enrichment	Multi‐step (physical‐biological)	US‐assisted germination (300 W, 60 h) → GABA (1:2 w/v, 30°C, 8 h) → centrifugation	Yield: 34.96% (TPC) and 43.17% (flavonoids) increased FA: ∼46%; TPC: 0.7.7% GAE Antioxidant activity enhanced across all treatments; most effective	Combines germination, US, and GABA stress; novel method for phenolic enrichment; demonstrates process synergy	Lab scale Apply US‐assisted germination with bioactive stress at pilot scale; combine with other cereals for phenolic‐rich functional ingredients; explore synergistic effects on antioxidant and bioactive enrichment	4.0
Cao et al. (2023)	Highland barley	β‐glucan (polysaccharide/fiber)	M + US extraction	Multi‐step (physical + enzymatic‐green)	M extraction (7 W/g, 2 min) → enzymatic purification (thermostable α‐amylase (20,000 U), starch glucosidase (10,000 U), pancreatin (5000 U)) → US modification (40 kHz, 900 W, 3 s on:5 s off pulse, 30 min)	Yield: 3.4% extractability β‐glucan: 93.42% Antioxidant activities DPPH 45%, ABTS 84%, Hydroxyl 68%, Superoxide 54%	Moderate US (30 min) enhances antioxidant activity via disaggregation; excessive US reduces function	Lab scale Optimize US intensity and duration for industrial‐scale β‐glucan extraction; assess formulation into functional foods or nutraceuticals	4.0
Li et al. (2021)	Highland barley grains (roller‐milled)	β‐glucan	M + US + enzymatic hydrolysis	Multi‐step (physical‐enzymatic)	M (defatting: 80% ethanol, 1:4 w/v, refluxed 2 h; drying: 50°C, 12 h; 800 W, 3 min) + US (50% amplitude, 50°C, 20 min) + EH extraction → centrifugation	Yield: 78.4% (core); 72.7% (innermost); 71.2% (second layer); 68.2% (third layer); 36.4% (outermost) β‐glucan: 87.17% (core); 87.06% (innermost); 85.20% (second layer); 82.80% (outrtmost) Viscosity increased with higher molecular weight (core layer maximum); higher initial foamability with low molecular weight; stable foams and more rigid with high molecular weight	Grain fractionation affects β‐glucan content; inner fractions ideal for high‐molecular weight functional β‐glucan	Bench scale Target inner barley fractions for high‐molecular weight β‐glucan extraction; integrate into functional foods, beverages, or nutraceuticals emphasizing molecular weight‐dependent functionality	4.1
Fall et al. (2025)	Highland barley bran	BPC, SDF, IDF, SCFAs	Structural disassembly → enzymatic release → bioconversion	Multi‐step (physical‐enzymatic‐biological)	M (300 W, 3 min) → cellulase hydrolysis (20 U/mL, 37°C) → germination (95% relative humidity, 30°C, 48 h) → in vitro digestion; centrifugation	Yield: SDF increased from 9.17% to 10.97% and IDF decreased from 59.28% to 46.27%; BPC enhancement 75.44%; high phenolic bioaccessibility (68.3%) SCFAs: 0.032% Antioxidant capacity followed the sequence: pretreated > post‐GI digestion > post‐colonic fermentation; strong prebiotic and antioxidant response	Sequential green valorization of bran into a functional fiber system	Lab scale Validate in vitro results in vivo; scale‐up M‐assisted processing	4.2
Wang et al. (2024)	Highland barley bran	Extractable and non‐extractable polyphenols	Chemical solubilization vs. physiological release benchmarking	Multi‐step (physical–chemical–biological)	US (*n*‐hexane defatted 1:5 w/v, 12 h; 300 W, 30 min) + fermentation; chemical vs. physiological extraction; SEM/FTIR/XRD characterization	Yield: 0.72% GAE TPC by chemical extraction; 0.27% GAE extractable; 0.45% GAE non‐extractable FA: 0.032% Stronger antioxidant signals, while physiological extraction revealed lower but bioaccessible fractions, exposing overestimation by conventional methods	First quantitative comparison of chemical vs. physiological polyphenol extraction; links matrix architecture to release behavior	Lab scale Validate physiological relevance in vivo; optimize extraction for realistic bioaccessibility	4.1
Xi et al. (2023)	Highland barley bran	SDF	Steam explosion → US → EH	Multi‐step (physical‐enzymatic)	Steam explosion (85% ethanol defatting 1:4 w/v, 12 h, 50°C; 1.5 MPa, 90 s) + US (1:10 w/v, 50°C, 1 h, 200 W) + enzymatic hydrolysis (celluclast 100 µL/10 g, pH 4.5) → centrifugation → ethanol precipitation	Yield: 20.01% alkaline; 10.69% EH; 7.92% US; IDFconverted into SDF from 67.72% (control) down to 57.11%–59.74% Arabinose: 38.85%; xylose: 42.23%; carbohydrates: ∼85%; protein: ∼5%; fat: ∼1% WHC 4.8 g/g; OHC 5.04 g/g; CAC 30.46 mg/g; NIAC 897.08 µg/g	Steam explosion US allows tunable extraction with strong functional properties	Pilot/semi‐industrial scale Industrial‐scale validation method; assess functional food incorporation and health effects	4.0
Liu et al. (2021)	Hull‐less barley bran	β‐glucan	M‐assisted US extraction	Linear/hybrid (physical‐green energy)	M+US extraction (80% ethanol 1:4 w/v defatting, reflux 2 h, drying 50°C, 2 h; 1:25 w/v ratio, 800 W, 1.5 min; 40 kHz, 500 W, 50% amplitude, 50°C, 10 min)	Yield: 2.16% High‐molecular weight β‐glucan: 86.58% Superior foam stability and emulsifying properties	M+US efficient in yield, time, and functional quality; recommended for industrial extraction	Lab scale Scale hybrid M + US extraction for industrial β‐glucan recovery; exploit greater functional properties for bakery, beverage, or emulsified products	4.0
Özen et al. (2024)	Hull‐less barley	β‐glucan	Pressurized M‐assisted green extraction	Linear (physical‐green solvent)	M‐pressurized CO_2_/H_2_O extraction (1:10.10 w/w ratio, 30 bar, 215 rpm, 47.74°C, 19.92 min); RSM optimization	Yield: 62.43% (17.5% higher) β‐glucan: 67.17% Greater solubility and WHC in minutes using only CO_2_ and water	First M‐pressurized CO_2_ method; rapid, low‐energy, and solvent‐safe	Bench‐to‐pilot scale Scale up M‐pressurized CO_2_ method; explore other safe solvent combinations for β‐glucan extraction	4.4
Álvarez‐Romero et al. (2023)	Barley	Phenolic compounds	M vs. US comparative	Linear (physical‐green)	M‐assisted (1:40 w/v, 80% v/v methanol, 100°C, 1100 W, 15 min) and US‐assisted (1:40 w/v, 71% v/v methanol, 64°C, 70 W, 10 min) extraction; RSM optimization	Yield: 25%–32% more than US TPC: 0.11%–0.18% GAE DPPH 1.35–3.37 mg TE/g; M slightly higher efficiency than US; extraction scalable	First direct comparison of M vs. US for industrial phenolic extraction; optimized via RSM	Bench‐to‐pilot scale Compare M vs US on pilot scale; optimize parameters for industrial phenolic extraction; explore functional ingredient applications	4.0
Hong et al. (2022)	Qingke (highland hull‐less barley)	Dietary fiber, β‐glucan, pentosan, tocopherols, phenolics	Thermal processing (boiling, steaming, M, frying, steam explosion, dry frying, trisaccharide treatment) comparative	Linear (physical‐thermal)	Thermal treatments (21 bar, 30 s) → solvent extraction for phenolics	Yield: ∼12.5% SDF; 15.9‐fold increase pentosan β‐glucan: up to ∼4.5%; tocopherols: up to 0.048%; –OH radical scavenging up to 446.06 mg ASC/g; ABTS and Fe^2+^ chelating measured	PCA shows steam explosion is best for retaining bioactives	Pilot/semi commercial scale Implement steam explosion at pilot/industrial scale for highland barley; combine with other green processing methods; develop functional food ingredients rich in β‐glucan and antioxidants	4.1
He et al. (2020)	Qingke (Tibetan hulless barley)	Polysaccharides (functional fiber)	Comparative green extraction	Linear (physical)	Hot water, US (24 kHz, 650 W, 70% amplitude, 20–25°C, 20 min), M (480 W, 85°C, 10 min)‐assisted extraction, PWE (1:3 w/v rato, 16 bar, 55°C, 40 min) → centrifugation → freeze‐drying	Yield 2.93%–3.62%; β‐glucan: ∼79%–86% Reducing power 41.83 µg Trolox/mg; NO scavenging 49.35%; Fat binding 2.49 g/g	PWE most effective; comparative study shows optimal method for bioactive polysaccharides	Lab scale Pilot green extraction of polysaccharides (PWE preferred); integrate comparative study insights to maximize β‐glucan bioactivity and functional fiber recovery	4.0
Fareed et al. (2025)	Barley malt residue	XOS, xylose	M‐induced matrix disruption → enzymatic saccharification	Multi‐step (physical‐enzymatic)	M‐assisted EH; biological purification (30 bar, 1000–1400 W, 2–6 min; 1:5 w/v, 1235.1 W, 6 min; xylanase dosage: 89.12 U/g substrate, 4 h, 50°C, 200 rpm); RSM optimization	Yield: 20.81% XOS Oligosaccharide: degree of polymerization 2–5 (xylobiose, xylotriose, xylotetraose, xylopentaose) Prebiotic activity comparable to commercial standards	First direct XOS production without harsh pretreatment; scalable and cost‐effective	Lab scale Assess industrial scalability and cost‐efficiency; integrate biological purification into production	4.3
Falade et al. (2025)	BSG	SDF (AX, β‐glucan)	US + M‐assisted alkali extraction	Multi‐step/Hybrid (physical–chemical)	US (28 kHz, 76% amplitude, 0.93 W/cm^2^ intensity, 0.89 W, 25 miv) + M (1000 W) → filtration/precipitation	Yield: 39.74% SDF AX: 34.2%; β‐glucan: 19.14%; xylose: 21.41%; arabinose: 13.38% Extraction time reduced from 60 to 16.25 min; polysaccharide integrity preserved	Intensified extraction; produces high‐quality fibers with selective polysaccharide enrichment	Bench‐to‐early pilot scale Scale up method for industrial SDF recovery from BSG; optimize extraction for selective AX or β‐glucan enrichment; integrate into prebiotic fiber products	4.1
Alonso‐Riaño et al. (2020)	BSG	Polyphenols, protein, sugars	US‐assisted extraction	Multi‐step (physical–biological)	US (750 W, 20 kHz, 47°C) → ultrafiltration	Yield: 24% TDF; 8.2% protein AX: 5.4%; TPC: ∼0.33%; protein retention up to 82%; FA: 0.0011% FRAP shows strong correlation; hydrolysis enhances esterified phenolics	Simultaneous recovery of polyphenols, protein, sugars; kinetic modeling confirms process efficiency	Lab/bench scale Expand US and ultrafiltration for simultaneous recovery of polyphenols, proteins, and sugars at larger scale; develop green, multi‐component BSG valorization streams	3.9
Zago et al. (2022)	BSG from light and red malted barley	Low‐molecular‐weight phenolic compounds: 4‐vinylguaiacol (4‐VG)	M + US‐assisted	Hybrid (physical‐green)	M (800 W, 30 min, 2450 MHz) pretreatment + US‐assisted solvent extraction (70% ethanol, 1:30 w/v, 80°C, 60 min)	Yield: 1.3% FAE TPC FA: 0.0034%; 4‐VG: ∼80% increased DPPH radical inhibition increased 5.8%–56.9%: 4‐VG DPPH inhibition 96% and FA 75%	Sustainable production of phenolics from BSG; extraction time is more influential than power	Lab scale Scale‐up M+US for BSG valorization; optimize extraction parameters for maximal low‐molecular‐weight phenolics; integrate into functional ingredients	4.0
Bonifácio‐Lopes et al. (2022)	BSG	Hydro‐ethanolic phytochemical extract	Ohmic heating extraction	Linear (physical‐green energy)	Ohmic heating (1:5 w/v; 60% ethanol, 50‐mL jacketed static ohmic cell, 8 cm distance electrodes, 45 and 75 V, 25 kHz, ∼35°C, 30 min) → filtration	Yield: 1.2% GAE TPC FA: 0.0036% ABTS 30.7 mg AAE/g; ORAC 42.62 mg TE/g; ACE inhibition IC_50_ 26.7 mg GAE/g	Pilot‐scale is green and efficient for antioxidants and antihypertensive extracts	Lab scale Scale up method for BSG valorization into antioxidant and antihypertensive extracts; investigate safe dosage levels and incorporation into functional foods or nutraceuticals	3.9
Iadecola et al. (2022)	BSG	Phenolic compounds	US‐assisted extraction	Linear (physical‐green energy)	US (1:2 w/v or 20:80 v/v, 65:35% ethanol; 37 kHz, 80°C, 50 min) → filtration; box‐behnken optimization	Yield: 0.41% GAE TPC; gained efficiency than conventional FA was predominant phenolic acid DPPH 0.42 mg TE/g; ABTS 5.82 mg TE/g	Rapid, inexpensive, and effective; suitable for food and industrial applications	Lab scale Apply US at pilot or industrial scale for BSG phenolics; explore use in food ingredients, supplements, or biorefinery valorization	4.0
López‐Linares et al. (2021)	BSG	Phenolic compounds	M‐assisted extraction	Linear (physical‐green solvent)	M‐assisted extraction using DES (ChCl:glycerol 1:2 molar ratio, 10% w/v S/L ratio, 37.46% v/v water, 100°C, 13.3 min) → vacuum filtration	Yield: 0.29% GAE TPC FA: 26.4%; *p*‐coumaric acid: 20.6% High solid recovery >84.4%; DES superior to 80% methanol	First DES‐assisted M‐extraction on BSG; environmentally friendly; residual solids are usable for biorefinery	Lab scale Combine M‐assisted DES extraction with BSG biorefinery; optimize residual solids use for fiber, protein, or biofuel streams; enhance green processing efficiency	3.9
Martín‐García et al. (2020)	BSG	Phenolic extracts	PEF	Linear (physical‐nonthermal)	PEF (1:1 w/w S/L ratio with 463 mS/cm electrical conductivity of suspension; 5 × 5 × 5 cm^3^ batch chamber with 5 × 5 electrodes, 22 mm spaced apart; 10 µs monopolar pulses; 2.5 kV/cm electric field strength, 50 Hz, 14.5 s; 9.06 kJ/kg energy input) → alkaline hydrolysis	Yield: +43.23% overall enhancement total phenolics; 2.7‐fold (+62.8%) increased free phenolics; 1.7‐fold (+39.6%) increased BPC Free phenolics: 0.01%; BPC: 0.54% Suitable as functional bioactive ingredients for dietary fortification; high concentrations of naturally occurring antioxidant flavanols, flavones, and hydroxycinnamic acid derivatives	PEF as pretreatment enhances phenolic recovery; nonthermal, energy‐efficient	Lab scale Apply PEF as green pretreatment for BSG valorization; optimize for pilot/industrial throughput; potential for high‐value phenolic extracts	3.9
Sukop et al. (2025)	Maize kernels	Fiber‐, starch‐, protein‐rich fractions	Wet mechanical fractionation → electro‐permeabilization	Multi‐step (physical‐electro‐physical)	Wet milling (pH 2.5, lactic acid, 0.2% SO_2_ from sodium metabisulfite) → sieving → sedimentation (50 mL/min flow rate) + PEF (1.5 kV/cm, 5 µs, 100 Hz, 4718 pulses, 17 kV, 202 A, 50 kJ/kg einput energy; electrodes 9 cm gap, 10 batches of 1620 g ground slurry at <25°C)	Yield: 39.6% (lab) 43.2% (pilot) fiber‐rich fraction; 47.4% (lab) 33% (pilot) starch‐rich; 4.5% (lab) 3.95% (pilot) protein‐rich fraction; 110.07% AX, 105.60% TDF, 28.51% protein, 65.79% starch, 32.88% BPC TDF: 26.7%, AX: 11.32%; starch: 90%–95.6%; protein: 43%–50.9% Improved water‐holding (+44%); high protein solubility (51.2%–52.3%); mild protein unfolding and exposure of hydrophilic domains while preserving overall protein conformation; higher peak viscosity (4323 cP) and breakdown viscosity (1347 cP) of starch‐rich fraction; fiber‐rich fraction had lower pasting viscosity (1625 cP) due to enhanced fiber welling and water competition against starch	First coupling of PEF with maize wet fractionation enhances nutrient recovery; ideal for gluten‐free bread/food matrices	Pilot‐scale validated in industrially relevant environment Evaluate industrial PEF; optimize energy and cost	4.4
Burlini et al. (2020)	Yellow and white corn germs	Phenolic extracts (FA) and lipophilic extracts (phytosterols)	Dual‐fraction cascaded extraction and enzymatic comparison	Multi‐step (physical–chemical)	SFE (300 atm, 40°C, 2.5 L/min CO_2_ flow rate, 10 min) + US (48 kHz, 30 min) + alkaline hydrolysis (2 M NaOH; 1:20 w/v)	Yield: 6.22% (yellow), 3.95% (white); 0.84% GAE (yellow), 0.74% GAE (white), 0.66% FAE (yellow), 0.57% FAE (white) TPC; 19.98% (yellow), 13.91% (white) oil *Trans*‐FA: 56.9%–63.7%; β‐sitosterol: 59.48%–60.72%; campesterol: 19.31%–20.98%; stigmasterol: 6.21%–6.99%; sitostanol: 7.8%, campestanol: 2.0%–2.5%, cycloartenol: 1.2%–1.9% DPPH IC_50_ 11.41–14.33 µg/mL	Sonication and hydrolysis maximize phenolic extraction; SFE effective for lipophilic compounds	Lab scale Develop multi‐step SFE + US + EH for maize germ at industrial scale; target both phenolic and lipophilic bioactives; explore food and nutraceutical applications	4.0
Jiang et al. (2025)	Corn bran	SDF	EH → thermal restructuring	Multi‐step (enzymatic‐thermal)	EH (1.5% w/w cellulose (2 × 10^6^ U/g), 1% w/w xylanase (10^5^ U/g) + high‐temperature cooking → ethanol precipitation (4 volumes of 95% ethanol at 4°C for 10 h)	Yield: 13.80% (∼3.9‐fold increase) SDF: 84.93%; xylose: 36.01%; arabinose: 35.17% 89.03% water solubility, −32.40 mV zeta potential, 701.1 m^2^ specific surface area; strong WHC, OHC, and glucose adsorption, enabling targeted functional ingredient design	Links molecular composition and crystallinity to functional traits	Lab scale Conduct in vivo studies; refine functional food ingredient design based on SDF properties	4.1
Garcia‐Ortiz et al. (2023)	Polyembryonic and non‐polyembryonic maize sprouts	Hydrolysable polyphenols, condensed tannins	M + US	Hybrid (physical‐green)	M (800 W, 5 min) + US (20 kW, 25 kHz, 20 min) extraction → filtration → resin purification	Yield: 2.86% (8.9‐fold higher) Phenolics: 3.39%; hydrolysable polyphenols: 0.56% GAE DPPH 12.04 mmol TE/100 g; FRAP 4.50 mmol TE/100 g; ABTS 14.34 mmol TE/100 g	Hybrid M + US extraction enhances bioactive recovery	Lab scale Validate hybrid method for commercial sprout bioactives; explore genotype‐specific valorization	4.1
Garcia‐Ortiz et al. (2022)	Red corn grains	Pigments (liquid), residual flour	M + US assisted	Hybrid (physical‐green)	M (800 W, 5 min) + US (25 kHz, 20 min) assisted extraction; liquid‐solid separation	Yield: intense orange‐red and yellow pigments (*a*∗ = 14.3–17.0, *b*∗ = 24.3–38.3); residual flour protein up to 11.16%, starch up to 61.3%, fat reduced 57% Solubilized anthocyanins, phenolics, and carotenoids Improved techno‐functional properties (water absorption 121.92%, oil, and organic molecule retention)	Enables pigment extraction while valorizing residual flour; green extraction approach	Lab scale Valorize residual flour from pigment extraction; explore functional ingredient applications and process energy efficiency	4.0
Boateng et al. (2023)	Purple corn pericarp	Polyphenol‐rich extract (anthocyanins, phenolics)	M → US	Linear/hybrid (physical)	Dry milling → US (500 W, 20 kHz, 50% amplitude, 28°C, 21 min) or M → filtration	Yield: 22.08% Cyanidin‐3‐*O*‐glucoside: 8.2%; cyanidin chloride: 5.6%; delphinidin: 3% TAC 34.99 g/kg; TPC 121.26 g/kg; CT 260.59 g/kg; DPPH 507.36 mg TE/g; ABTS 793.81 mg TE/g	US improves phenolics profile, structure, and product quality vs. M; nonthermal and energy‐efficient method	Lab scale Scale M + US extraction; compare methods for energy efficiency and bioactive stability	4.1
García‐Ortíz et al. (2023)	Red corn	Pigment extract (polyphenols, tannins)	Hybrid M–US	Linear/Hybrid (physical‐green)	M (800 W, 10 min), US (25 kHz, 20 min), hybrid extraction	Yield: 0.6% (25% higher) Semi‐purified natural flavonoid‐rich pigment powder; free of water‐soluble sugars and non‐target polar co‐extractives after fractionation Highest antioxidant activity with hybrid extraction	Hybrid outperforms M or US alone; green, efficient, maximizes bioactive recovery across varieties	Lab scale Scale hybrid M–US extraction; evaluate energy efficiency and bioactive stability across corn varieties	4.1
Kumar et al. (2025)	Purple corn pericarp	Anthocyanins, tannins, flavonoids	Solvent‐mediated molecular extraction	Linear (physical‐green solvent)	US (20 kHz, 500 W, 30% amplitude, 10 min)‐assisted DES (Glycerol:Lactic acid 2:1; 1:50 ration S/L); filtration	Yield: anthocyanin (2.7%); tannin (24.9%); flavonoids 2.6% Cyanidin‐3‐*O*‐glucoside: 2.1%; cyanidin chloride: 1.2%; delphinidin: 1%; FA: 0.2% Recovery translated into very strong antioxidant capacity (CUPRAC 392 mg TE/g)	DES tunability enables selective phytochemical fractionation	Lab scale Assess energy use; pilot DES solvent recovery	4.0
Olalere and Gan (2023)	Defatted wheat germ	Protein extract	M‐assisted DES	Multi‐step (physical–chemical)	M (*n*‐hexane defatted with 1:5 w/v; 186 W, 3.28 min)‐assisted extraction using alkaline‐based DES (K_2_CO_3_‐glycerol 0.52 g/mL; 1:39 w/v)	Yield: 33% Polypeptide bands resolved at 35, 25, and 11 kDa (hydrophobic prolamins and glutenins <48 kDa), with minor bands at 245, 180, 63, 22, and 17 kDa	First use of DES for wheat germ protein extraction; optimized via Box–Behnken design; practical and scalable; optimal absorbed M energy 677 J/min	Lab scale Scale DES‐based protein extraction; assess applicability in food formulations; evaluate solvent reuse and environmental footprint	4.1
Xie et al. (2023)	Defatted wheat germ	Wheat germ protein	US + alkaline extraction + isoelectric precipitation	Multi‐step (physical–chemical)	US (1:25 w/v; 400 W, 10 min)‐assisted extraction + alkaline extraction (pH 9) + isoelectric precipitation	Yield: 36.65 recovered rate; 88.66% extraction efficiency WHC 2.55 g/g; OHC 4.86 g/g; superior foaming and emulsifying; form a stable interfacial viscoelastic film around air droplets	US improved extraction efficiency and functional properties; ultrasonic cavitation breaking internal hydrophobic bonds and enhancing molecular dispersion	Lab scale Scale US + alkaline extraction; integrate into plant‐protein products; evaluate environmental footprint	4.3
Pan et al. (2025)	Defatted wheat germ meal	Soluble proteins, peptides, phenolics, GABA	Matrix disruption → enzymatic release → microbial bioconversion	Multi‐step (physical‐enzymatic‐biological)	US (40 kHz, 80 W, 5 s pulse regime, 30 min) + EH (amylase 300 U/mL, pH 6.9, 37°C, 30 min; cellulase 300 U/mL, pH 5.2, 50°C, 30 min) → fermentation (1%—450 µL *Bacillus subtilis* B‐2 inoculum, 30°C, 72 h) → ultrafiltration (3 and 10 kDa molecular weight cutoffs)	Yield: +139.72% increased Polypeptides: 0.037% (<3 kDa); TPC: 0.063% GAE; amino acids: +42.85% increased, total essential and total amino acids ratio: 80.73% GABA rose 10‐fold; peptides showed the highest antioxidant activity (40.54% ABTS and 18.53% DPPH) with enriched in valine (810.87%), histidine (5.97%), arginine (3.40%)	Integrated physical–biological cascade efficiently generates bioactive proteins	Lab scale Simplify multi‐step process; enhance yield and efficiency	4.1
Tian et al. (2022)	Wheat germ albumin	Antioxidant polypeptides (<3 kDa)	M → EH → purification	Multi‐step (physical‐enzymatic)	M (600 W, 65°C)‐assisted hydrolysis (9.32 × 10^3^ U/g, pH 7, 9 min) + ultrafiltration (5 and 10 kDa cutoff, 0.1 MPa; 3 kDa cutoff, 0.15 MPa) + gel chromatography	Yield: 50.05% <3 kDa; 33.69% >10 kDa; 10.64% 5–10 kDa; 5.62% 3К5 kDa <3 kDa peptide pool (gel fraction): Short‐chain oligopeptides (1–3) and inactive single amino acids (4) DPPH EC_50_ 1.78 mg/mL; O_2_^•−^ scavenging IC_50_ 4.08 mg/mL	M‐assisted hydrolysis reduces processing time; purification yields potent antioxidant fractions	Lab scale Scale M + EH for purified antioxidant peptides; test bioactivity in food or nutraceutical matrices; evaluate process efficiency and cost	4.2
Nemes et al. (2025)	Wheat bran	Organic acids, phenolics	Hybrid pretreatment → solid‐state bioconversion	Multi‐step (physical–biological)	US (20 kHz, 152–750 W, 5 min pulse cycles) + M (300–400 W, 2 min) pretreatment → SSF → solid–liquid separation	Yield: 1.5‐fold higher TPC (+21.30% increased) Vanillic acid: 0.12%; protocatechuic acid: 0.026%; lactic acid: 2.2%; acetic acid: 0.3% Phenolics and organic acids with a strong antioxidant response; enriched in liberated phenolic aglycones, short‐chain organic acids, and simple monosaccharides	First integration of US + M + SSF for bran valorization	Lab scale Assess industrial feasibility; optimize energy use; continuous processing	3.9
Hassan et al. (2025)	Wheat bran commercial	Fermented bran ingredients	Enzymatic depolymerization → microbial bioconversion	Multi‐step (enzymatic‐biological)	Xylanase hydrolysis (55°C, 12 h) + SSF (37°C, 24 h) → drying → incorporation into bread	Yield: 6.9% (∼6.3‐fold increase) AX Free phenolics: 0.7% GAE (∼2.9‐fold increase); BPC: 0.3% GAE High phenolic content translated into antioxidant‐active functional bread at 15% inclusion; increased WHC, water and protein solubility	Demonstrates direct food application of fermented bran fraction	Pilot scale food formulation Explore broader microbial consortia and functional food applications; scale‐up studies needed	4.0
Rudjito et al. (2024)	Wheat bran	Feruloylated AX, AXOS	Hydrothermal‐enzymatic biorefinery	Multi‐step (physical‐enzymatic)	SWE (1:14 w/v; 10–11 MPa, 160°C, 35 min) → EH (37°C, 48 h)	Yield: 74%–75% AX FA: 0.12%; preserved high molar mass (10–100 kDa) Functional release of bound hemicellulose; increased SCFAs and high purity prebiotics (AXOS) production	Integration of SWE + EH enhances AX and FAX release; scalable, circular approach	Pilot‐scale validated in industrially relevant environment Evaluate in vivo prebiotic/health effects; optimize SWE + EH for commercial functional food ingredients	4.3
Chen et al. (2020)	Black wheat bran	Phenolic extracts	Extrusion + US‐assisted extraction	Multi‐step (physical‐thermal)	Extrusion + US with two‐step orthogonal optimization (35 kHz, 300 W, 50°C, 40 min)	Yield: 0.3% GAE Chlorogenic acid: 0.2% DPPH scavenging 85.5%; IC_50_ almost 0.4 mg/mL	Sequential optimization enhanced phenolics and antioxidant activity; cultivar‐specific design	Pilot scale Scale extrusion + US for black wheat bran; assess cultivar‐specific optimization for industrial application; integrate antioxidant‐rich extracts into functional foods	4.0
Yilmaz‐Turan et al. (2020)	Wheat bran	FAX and protein‐rich isolate	Cascade extraction (SWE + protein isolation)	Multi‐step (physical‐biochemical)	SWE (1:14 w/v; 10–11 MPa) → protein isolation via lactic acid fermentation (35°C, 8 h) + alkaline extraction + EH (α‐amylase, 37°C, 5 h)	Yield: ∼70% Protein recovery: ∼50%; AX: 79.8%–93.1%; FA: 0.8%; FAX: 100 kDa DPPH EC_50_ almost 10–30 mg/mg	Demonstrates multi‐step valorization of wheat bran; recovers both fiber and protein fractions from same biomass	Pilot‐scale validated in industrially relevant environment Integrate SWE + protein isolation into pilot‐scale cascade valorization; explore functional food or nutraceutical applications; optimize extraction for energy efficiency	4.0
Martín‐Diana et al. (2021)	Wheat bran	FA; SDF; phenolic compounds	EH + hydrothermal and pressure + spray‐drying + microencapsulation	Multi‐step (enzymatic‐thermal‐physical)	EH (1% w/w, 47°C, pH 5, 20 h) → thermal pressure (hydrothermal: 115°C, 15 min; high‐hydrostatic‐pressure: 608 MPa, 5 min) → spray drying (130°C inlet, 85°C outlet)	Yield: ∼53% with pressure; ∼22% without pressure *Trans*‐FA: 0.25%; phenolics: 0.93% GAE: TDF: 15.21%; β‐glucan: 2.5%; protein: 10.36%; phytic acid: 0.85% DPPH almost 3000 µmol TE/100 g; tumor necrosis factor‐α inhibition up to 54%	Multi‐step, scalable, energy‐efficient route Optimal process combination preserves bioactivity	Pilot‐scale validated in industrially relevant environment Scale‐up multi‐step process; explore microencapsulation for functional foods; test in vivo bioactivity and stability	4.3
Cherif et al. (2020)	Soft wheat bran	Polyphenol extracts	DES‐assisted green extraction	Multi‐step (physical–chemical)	US‐assisted extraction (1:30 w/v; 100 Hz, 120 W, 120 W/L energy density, 50°C, 40 min) + DES (glycero:citric acid:glycine 4:1:1) + thermal extraction (90°C, 24 h)	Yield: 9.5% FAE FA: 0.1% AAR 283.04 µmol DPPH/g; PR 124.47 µmol AAE/g	Combining US, DES, and thermal extraction enhances yield; novel DES formulation applied	Lab scale Expand US + DES + thermal extraction for industrial polyphenol recovery; explore different DES formulations and eco‐friendly solvents; assess functional ingredient applications	4.0
Guerrini et al. (2020)	Durum wheat bran	Phenolic extracts (antioxidant/antimicrobial)	US‐assisted + alkaline hydrolysis	Multi‐step (physical–chemical)	US (48 kHz, 25 min), alkaline hydrolysis (2 M NaOH; 1:20 w/v) → filtration → evaporation/lyophilization	Yield: 0.48%–0.54% Total phenolics: 61.1% GAE: *trans*‐FA: 40.6% DPPH IC_50_ 3.61 µg/mL; ABTS IC_50_ 1.16 µg/mL	Sustainable extraction; extract applied in cosmetic formulations	Lab scale Apply method at pilot scale for durum wheat and rice bran; incorporate extracts into cosmetic or nutraceutical products; optimize yield vs. energy consumption	4.1
Li et al. (2020)	Wheat bran, milling by‐product	AX hydrogels (functional biopolymer)	Hydrogel fabrication via enzymatic crosslinking	Multi‐step (physical‐biochemical)	SWE (1:10 w/v; 160°C, pH 7, 10 min) + enzymatic crosslinking (laccase, 25°C, 30 min) → precipitation	Yield: 15.95%; 17.98% AX; 19.62% FA recovery AX: 93%; FA: 1.5%; high molecular weight: ∼389 kDa; (1 → 4)‐β‐d‐Xylp: 62%–65%; mono‐substituted at *O*‐3: 15%; di‐substituted at *O*‐2,3: 10%–13% Functional property: gelling ability (storage modulus *G*′); laccase oxidized monomeric FA ester substituents into crosslinking 8–5‐diferulate bridges	Green fabrication of functional hydrogels; focuses on material properties for bioactive applications	Lab scale Develop SWE + enzymatic crosslinked AX hydrogels at industrial scale; explore functional biomaterials or food hydrogel applications; optimize gel properties for specific uses	3.9
Ferri et al. (2020)	Wheat bran, industrial	FA extract	Multi‐enzyme EH	Multi‐step (enzymatic)	Rehydration (120°C, 1 bar, 20 min; 1:20 w/v) → multi‐enzyme system (2.6 U/g alcalase + 200 U/g termamyl, pH 6.4, 60°C, 1 h) → solid phase extraction	Yield: 0.11% (40‐fold scale‐up), 0.087% (lab) Soluble proteins: 15.45%; phenolics: 0.077% GAE Strong free‐radical scavenging	Scalable, non‐toxic, industrially feasible; optimized for 40‐fold bioreactor scale‐up	Benche scale with 40‐fold scale‐up Scale multi‐enzyme EH for FA recovery from wheat bran; integrate into circular biorefinery; assess industrial feasibility and energy/resource efficiency	3.8
Chen et al. (2022)	Wheat bran	BPC	M or US‐assisted extraction comparison	Multi‐step (physical–chemical‐green)	Defatting → alkaline hydrolysis → US (240 W, 48 min) + M (420 W, 2 min)	Yield: 0.85% GAE (US); 0.83% GAE (M) Total phenolic acids and flavonoids: 0.35% (US), 0.36% (M); FA: 0.29% (US), 0.31% (M) DPPH 77.5% (US), ABTS 72.1% (M)	M is more energy‐efficient and faster; US confirms lab‐scale effectiveness	Lab scale Pilot‐scale M and US comparison; standardize quantification; develop functional ingredients from wheat bran phenolics	4.1
Bautista‐Expósito et al. (2020)	Wheat bran, milling by‐product	Soluble wheat bran hydrolysate (phenolic acids, ferulic acid)	EH + hydrothermal treatment	Multi‐step (enzymatic‐thermal)	Hydrothermal pretreatment (121°C, 1 bar (1 atm), 30 min) → EH (cellulase, xylanase, α‐L‐arabinofuranosidase, and feruloyl esterase complex 1%; 47°C, pH 4.4, 20.8 h; 1:20 w/v) → solubilization → filtration → spray drying or further processing	Yield: 79.15%–83%; monosaccharide recovery 87.26% FA: 0.8%; diferulic acid isomers (1–2): 0.6%: vanillin FA: 0.23% ORAC 22.67 g TE/100 g; DPPH 5.90 g TE/100 g; MCP‐1 inhibition 36.1%	Sustainable valorization of wheat bran into nutraceuticals; RSM and enzyme screening are critical for optimization	Lab scale Scale enzymatic + hydrothermal wheat bran valorization; optimize enzyme cocktails; conduct in vivo efficacy studies for nutraceutical use	4.1
Li et al. (2025)	Wheat bran, milling by‐product	SDF	CP + EH and comparative	Multi‐step (physical‐enzymatic)	CP (0.75 A, 20 s) → EH (1:25 w/v, 0.004% acidic xylanase + 0.001% α‐l‐arabinofuranosidase; pH 5.0, 50°C, 2.5 h) → ethanol precipitation	Yield: 7.07% (CP), 15.08% (EH), 14.65% (CP + EH) Molecular breakdown: depolymerized to ∼9.3 (CP), ∼5.5 (EH), ∼5.2 (CP + EH) kDa; xylose: 39.56%, arabinose: 18.93%, AX: >58% (CP + EH) Functional properties: GAC 363.58 mg/g, α‐AAIR 30.58%, CAC 90.22 µmol/g, BSAC 5.89 mg/g, T‐AOC 16.83 U/mL; greater water solubility (∼85%)	Combined treatment improves yield and physicochemical/functional properties; first use of CP for wheat bran SDF	Lab scale Scale‐up CP+EH for wheat bran SDF production; investigate incorporation into functional foods, prebiotics, or nutraceutical ingredients; optimize for energy efficiency and product quality	4.1
Sun et al. (2025)	Wheat bran, milling by‐product	FAX (prebiotics)	Fermentation (1:1 w/v; 13% *B. subtilis* dose; 37°C, 36 h) + acid‐assisted autohydrolysis	Multi‐step (biological‐chemical)	SSF + acid‐assisted autohydrolysis gradient ethanol precipitation	Yield: 6.6% (12.5‐fold increase) FA: 0.45%; arabinose: 10.28%; xylose: 15.8%; AX: branching degree: 19.1; oligomeric/polymeric pools: 6.25 kDa (17.09%), 2.82 kDa (20.34%), 1.49 kDa (46.18%), 0.79 kDa (12.49%), 0.38 kDa (3.89%) Produced highest SCFAs (31.04 mM), favorable butyric‐acetic acid ratio	Combines green fermentation with autohydrolysis; fractionation allows selective prebiotic activity	Lab scale Optimize fermentation and autohydrolysis for selective FAX fractionation; explore prebiotics in gut health formulations and functional foods	4.0
Cavallero et al. (2025)	Wheat bran, milling by‐product	Polyphenols, flavonoids	Hybrid‐assisted solvent fractionation	Linear/hybrid (physical)	US (50% amplitude, 3 cycles of 10 s pulse), M (550 W, 70°C, 5 min)	Yield: 0.3% GAE, 0.058% QE Rich in *trans*‐FA Optimized solid/liquid ratio significantly elevated polyphenol yield, producing extracts with strong antioxidant and antibacterial activity	Hybrid extraction enhances bran valorization	Lab scale Scale up extraction; test in functional foods	3.9
Zhuang et al. (2024)	Wheat bran, milling by‐product	Soluble carb‐protein extract (prebiotic)	Dual EH	Linear (enzymatic)	Alcalase (0.8% v/w) + *Bacillus velezensis* hydrolase (2% v/w) → solid–liquid separation	Yield: 28% (11.4% protein, 18.5% carb increased) Proteins: 31.15%; sugars: 41.89%; total phenols: 1.83%; total sugars: 41.89% DPPH >60%; –OH radical scavenging almost 60%; enhanced in vitro butyrate production; bioactive prebiotic functionality	Dual‐enzyme treatment simultaneously enhances antioxidants and prebiotic value	Lab scale Scale dual‐enzyme process; in vivo validation of prebiotic effects; integrate into functional food product development	4.1
Spaggiari et al. (2023)	Triticum durum (whole grains and flour)	Phytochemical–azelaic acid	US, Naviglio, traditional maceration comparison	Linear (physical‐green)	US (1:10 w/v; 30 min), cyclic solid–liquid dynamic extraction	Yield: 3.5% (Naviglio), 0.96% (maceration), 0.4% (US) TPC: 0.091% GAE MIC 0.176% to >5% v/v	Extracts have antibacterial and antioxidant properties; Naviglio is most effective	Lab‐to‐exploratory scale Explore scale‐up and formulation into topical or mild food applications; optimize extraction for higher azelaic acid yield	3.8
Kirusnaruban et al. (2025)	Wheat kernels/flour, commercial	Phenolic acids	Thermo‐induced matrix disruption	Linear (physical–chemical)	M‐assisted extraction (400 W, 170°C, 15 min)	Yield: 0.5% GAE Gallic acid: 0.0092%–0.18% Enriched in secondary metabolites (derivatives of hydroxybenzoic and hydroxycinnamic acids); demonstrating rapid water‐based phenolic intensification	Time‐efficient green alternative to solvent extraction	Lab scale Benchmark against US; optimize M‐chemical extraction	3.8
Argun (2022)	Wheat, coarse bran, and razmol shorts	Phenolics, flavonoids, lipids	SFE	Linear (physical‐green)	Particle classification → SFE extraction (23–33 MPa, 60°C, 120 min)	Yield: 1.31%–1.60% (bran), 0.33%–0.38% (shorts) TPC: 0.18%–0.22% (bran), 0.36%–0.48% GAE (shorts) PUFA: ∼55%–59% (bran), ∼57% (shorts), almost 57%; linoleic acid: 49.92%–53.96%; oleic acid: 21.87%–25.62%; palmitic acid: 15.19%–17.48%; linolenic acid: 3.19%–3.80% ABTS 8.4–16 mg TE/g	SFE is eco‐friendly; finer fractions (shorts) enhance bioactive recovery	Lab scale Explore industrial SFE process; optimize for phenolic and PUFA yield; develop functional food applications	4.1
Habuš et al. (2021)	Wheat bran	Functional wheat bran with reduced PPO activity	High‐Intensity US + micronization	Linear (physical‐mechanical)	Micronization → high intensity US (400 W, 24 kHz, 80% amplitude; 10 µm, 10–15 min)	Yield: ∼11% phenolics increased PPO activity: 93% reduced Ultrasonic cavitation increased hydration and soluble fiber functionality	US and micronization improve technological properties and prevent browning	Lab scale Scale method; explore functional food applications and process energy optimization; combine with other treatments for enhanced bioactive retention	3.9
Razem et al. (2025)	Wheat bran	Phenolics, flavonoids	Solvent‐assisted molecular extraction	Linear (physical)	US (70% amplitude, 24 kHz, 400 W, 40°C, ethanol, 25 min) → evaporation	Yield: 1.3% GAE, 0.2% CE 4‐hydroxybenzoic acid: 0.21%; FA: 0.0075% Exceptionally high phenolic and flavonoid recovery produced potent antioxidant activity (DPPH, CUPRAC)	Advanced analytical tracking of antioxidant decay pathways	Lab scale Improve thermal stability via encapsulation; scale‐up process	3.9
Sahu et al. (2021)	Wheat whole grain, bran, flour	Defatted fractions and lipid‐phenolic extracts	SFE vs. hexane comparative	Linear (physical‐green solvent)	Defatting (350 bar, 50°C, 15 g/mL CO_2_ flow rate, 5 h), solvent fractionation (hexane)	Yield: 2.1%–2.6% (5.2%–9.6% more lipid than hexane) TPC: ∼0.09% GAE; FA: 0.0092% DPPH almost 55 mg TE/g; ABTS almost 280 mg TE/g; α‐amylase inhibition	Demonstrates greener, effective fractionation of wheat fractions; preserves phenolics and bioactivity; bran feasibility confirmed	Lab scale Industrial SFE fractionation; combined with valorization of phenolics; assess energy efficiency, solvent recycling, and scale‐up feasibility	3.9
Cengiz et al. (2021)	Wheat bran (einkorn, emmer)	Phenolic acid extracts	US‐assisted extraction	Linear (physical)	US (37 kHz, 120 W, 100% irradiation power) → filtration; US optimized via RSM and CCRD	Yield: 0.19% 2‐hydroxycinnamic acid: 0.87%; salicylic acid: 0.039%; *trans*‐FA: 0.034% –OH and phenolic core structures and free scavenging capabilities	Rapid, low‐solvent, effective extraction; suitable for food and nutrition applications	Lab scale Apply US at pilot/industrial scale; explore functional ingredient development; energy optimization for large‐scale use	3.8
Bhattacharya et al. (2020)	Wheat bran, industrial	AXOS (prebiotic)	EH	Linear (enzymatic)	Sequential EH (pH 7.5; 30°C, 6 h; 50°C, 24 h) → ultrafiltration (30 kDa membrane)	Yield: 67.37% water‐soluble AX; 30.6% AXOS (1.6‐fold increase over single‐EH) Arabinose xylose ratio: ∼1.5; degree of polymerization: 2–4 Promotes gut bacteria growth and SCFAs production (∼0.90 g/L butyric acid)	Functional prebiotic production from highly substituted AX	Lab‐to‐exploratory scale Develop prebiotic AXOS from wheat bran at scale; integrate with gut health product formulations; assess synergistic enzyme processes for efficiency	4.0
Arslan et al. (2020)	Wheat germ	Stabilized wheat germ and lipid (tocopherols, tocotrienols)	Moist heat (steaming), dry heat, M	Linear (physical‐thermal)	Physical stabilization + M (2450 MHz, 180–360 W, 12–5 min) → filtration → rotary evaporation	Yield: 0.096%–0.098%; α‐tocopherol: 0.049%; β‐tocopherol: 0.037% FFA: 5.5%–2.7% reduced; total oxidation: lowered value DPPH inhibition 48.9%–64.9%	Compares multiple stabilization approaches; steaming is most effective, microwave also viable	Pilot/pre‐industrial comparative scale Optimize heat and microwave stabilization for oils; combine with functional ingredient production; evaluate shelf life and bioactive retention	3.9
Al‐Hilphy et al. (2020)	Wheat bran	Total phenolics	Ohmic heating‐assisted extraction	Linear (physical‐green energy)	Ohmic heating‐assisted extraction (60 Hz, 60–220 V to 4.28–15.71 V/cm, 12–4 min, 30–96°C) → filtration	Yield: 0.046% Polar bioactive compounds: FA, dimers, sinapic, *p*‐coumaric, syringic, vanillic, and benzoic acids Antioxidant effectiveness 56%–84%	Pilot‐scale unit is energy‐efficient (63% savings); promising for food bioactive extraction	Pilot scale Scale pilot method for industrial bioactive extraction; integrate into functional foods or natural antioxidants; optimize field strength and time for maximum yield and energy efficiency	3.9
Rebolleda et al. (2020)	Wheat bran (*Triticum aestivum*)	Oily extract (alkylresorcinols, tocopherols, steryl ferulates)	Lipophilic extraction by SFE	Linear (physical‐green solvent)	SFE (250 bar, 40°C, 120 min) → separator	Yield: 2.5% Alkylresorcinols: 4.7%; tocopherols: 0.7%; steryl ferulates: 1.8%; total fatty acids: 71.2% (PUFA: ∼63%, linoleic acid: ∼58%) ABTS 270 µmol TE/g; DPPH 26 µmol TE/g	Effective for lipophilic bioactives; first quantification of steryl ferulates in wheat bran extract	Semi‐pilot scale Scale SFE extraction of lipophilic bioactives; develop refining/storage strategies for high‐FFA extracts; industrial valorization for antioxidant‐rich ingredients	3.9
Xiao et al. (2024)	Sorghum grains, distiller's/flours	Protein isolates	Enzymatic‐assisted wet fractionation	Multi‐step (enzymatic‐physical)	α‐Amylase (2.5% v/w; 70°C, 2 h) ± cellulase (2.5% v/w; 50°C, 1 h) hydrolysis → wet/dry milling → protein isolation	Yield: 9.1% (flours), 64.25% (distiller's grain); protein recovery rate 91.92%–92.70% (up to 98.75%) Protein: 84.76%; monomeric α‐kafirins: 20–28 kDa); cross‐linked dimeric/trimeric kafirin complexes: 37–75 kDa; high‐molecular weight: >75 kDa: α‐helix: 16.73%–31.75%; random coil 41.49%–53.01%, with lower β‐sheet 22.96%–35.01% Improved WHC (2.8–6.1 g/g), OHC (3.5–6.7 g/g), and in vitro digestibility (85%–86%), highest solubility (68.8%–82.8%), highlighting efficient liberation of storage proteins	A combination of α‐amylase and cellulase significantly enhances protein recovery and purity; sorghum protein is comparable to soy and pea	Lab‐to‐pilot scale Scale up combined α‐amylase and cellulase treatment; explore other varieties and application in plant‐protein products	4.2
Baah et al. (2025)	Decorticated white sorghum meal (Macia)	Modified sorghum meal with altered starch digestibility, phenolic extract	M‐assisted + phenolic addition	Linear (physical–chemical)	M (250 W, 15 min) heat moisture treatment + phenolic extract addition	Yield: 1.1% CE α‐Helix: 83.1% Resistant starch increased 28.21%–55.87%; starch hydrolysis index reduced 69.89%–54.33% and GI lowered 78.08%–56.57%	Improves resistant starch; useful for modulating digestibility	Lab scale Integrate method and phenolic fortification to produce sorghum‐based functional foods with high resistant starch; explore digestibility modulation and in vivo metabolic effects	3.8

Abbreviations: α‐AAIR, α‐amylase activity inhibition ratio; AAE, ascorbic acid equivalent; AAR, antiradical activity; ABTS, 2,2′‐azino‐bis(3‐ethylbenzothiazoline‐6‐sulfonic acid) radical scavenging assay; ACE, angiotensin‐I converting enzyme; ASC, ascorbic acid content; AX, arabinoxylan; AXOS, arabinoxylan oligosaccharides; BPC, bound phenolic content; BSAC, bile salt adsorption capacity; BSG, brewer's spent grain; CAC, cholesterol adsorption capacity; CCRD, central composite rotatable design; CE, catechin equivalent; CP, cold plasma; CT, condensed tannins; CUPRAC, cupric ion reducing antioxidant capacity assay; DES, deep eutectic solvents; DPPH, 2,2‐diphenyl‐1‐picrylhydrazyl radical scavenging assay; EC_50_, effective concentration for 50% activity; EH, enzymatic hydrolysis; FA, ferulic acid; FAE, ferulic acid equivalent; FAX, feruloylated arabinoxylan; FFA, free fatty acids; FRAP, ferric reducing antioxidant power assay; FTIR, Fourier transform infrared spectrometry; GABA, γ‐aminobutyric acid; GAC, glucose adsorption capacity; GAE, gallic acid equivalent; GI, glycemic index; IC_50_, inhibitory concentration for 50% activity; IDF, insoluble dietary fiber; kDa, kilodalton; LCA, life‐cycle assessment; M, microwave; MCP, monocyte chemoatractant protein; MIC, minimum inhibitory concentration; NIAC, nitrite ion adsorption capacity; NSPH, non‐starch polysaccharides hydrolysis; –OH, hydroxyl radical scavenging activity; OHC, oil‐holding capacity; ORAC, oxygen radical absorbance capacity; PCA, principal component analysis; PEF, pulsed electric field; PPO, polyphenol oxidase; PR, ferric‐reducing Power; PUFA, polyunsaturated fatty acids; QE, quercetin equivalent; RSM, response surface methodology; SCFAs, short‐chain fatty acids; SDF, soluble dietary fiber; SEM, scanning electron microscopy; SFE, supercritical fluid/CO_2_ extraction; SSF, solid‐state fermentation; SWE, subcritical water extraction; TAC, total anthocyanins; T‐AOC, total antioxidant capacity; TDF, total dietary fiber; TE, trolox equivalent; TPC, total phenolic content; US, ultrasound; WHC, water‐holding capacity; XOS, xylooligosaccharides; XRD, x‐ray diffraction.

^a^Score (1–5) refers to a combined (semi‐quantitative) assessment of (i) extraction efficiency and functionality; (ii) process sustainability and green metrics; (iii) scale‐readiness; and (iv) robustness of experimental validation.

#### 3.1.1 Separation and Recovery Efficiency

Nutrient recovery efficiency is fundamentally driven by the choice of extraction techniques and the structural properties of the biomass matrix. The comparative performance of various fractionation methods was analyzed and discussed with respect to the recovery of proteins, dietary fibers, oils, and phytochemicals from different cereal biomasses. Table 3 presents the standardized and stratified recovery‐yield outcomes across different processing sequences. The studies were categorized by processing approaches, such as linear versus integrated biorefinery systems. However, standardization does not eliminate methodological heterogeneity because the included studies varied in feedstock matrix, pretreatment, extraction conditions, fraction definition, product purity, and yield calculation; the values should be interpreted as reported recovery ranges within the reviewed processing strategies (technology‐specific standards) instead of as direct measures of intrinsic technology (standardized efficiency). Table 2 retains the study‐specific processing conditions, product characteristics, functionality, and technological opportunities, whereas Table 3 quantitatively aggregates standardized recovery yields. This distinction reflects that a higher recovery value may reflect differences in biomass composition, extraction severity, or product definition rather than superior process efficiency. The mechanistic effectiveness of agricultural biomass valorization depends on balancing cellular energy disruption with the preservation of thermolabile bioactives. The specific geometrical configuration of these matrices defines the functional, nutritional, and structural integrity of the final product. Variations in extraction yield among technologies can be attributed to their differing effects on cell disruption mechanisms. Strong oxidative forces can directly cleave lignin in cell walls, altering solvent polarity and enhancing mass transfer of intracellular compounds (Jeevarathinam et al. 2026). Correspondingly, M and US interventions induce rapid volumetric heating or physical cavitation, which effectively breaks the noncovalent bonds adhering bound phenolic compounds to dietary fibers (Rocchetti et al. 2022). The specific nanoscale interaction between starch and protein allows for more efficient hydration and component separation during milling (Sivakumar et al. 2023). Protein nanoparticles spontaneously self‐assemble at the oil and water interface, forming robust mechanical barriers that make them more efficient at stabilizing pickering emulsions (Yang et al. 2025). Thus, recovery and purity should be interpreted together with processing intensity, functional retention, resource requirements, and industrial readiness when comparing fractionation technologies. In addition, no single fractionation technology consistently provides the highest recovery, purity, functionality, and industrial feasibility simultaneously. Mechanical approaches offer greater process maturity and simpler operation, whereas intensified and enzymatic technologies can improve selectivity or functional performance at the expense of process complexity, equipment requirements, residence time, or operating cost. SFE is particularly suited to high‐value lipophilic fractions, whereas OH and US have great scale‐up potential where their energy or extraction benefits may balance the need for equipment and process control.

**TABLE 3:** Cereal biomass fractionations by various biorefinery strategies, with major ingredient yields analyzed.

No. of studies	Feedstock per studies with applied technology strategies	Recovery yields (% w/w)	Mean	Median	SD	Min–Max	IQR (25%–75%)	References
5	Linear: oat (2) (SFE, EH); wheat (2) (M) (US); sorghum (1) (EH)	Protein	57.86	55.80	20.72	33.00–88.66	16.65	Gaber et al. (2025), Olalere and Gan (2023), Sargautis and Kince (2023), Xiao et al. (2024), Xie et al. (2023)
2	Integrated: wheat (1) (M + EH); maize (1) (M + US)		30.61	30.61	27.50	11.16–50.05	19.45	Garcia‐Ortiz et al. (2022), Tian et al. (2022)
7	Linear: oat (2) (US, EH); barley bran (2) (M, US); wheat bran (2) (CP, EH); maize bran (1) (EH)	Fiber/prebiotic	25.74	13.80	32.97	1.45–90.00	28.47	Ibrahim, Nadeem, Khalid, et al. (2024), Jiang et al. (2025), Li et al. (2025), Liu et al. (2021), Sargautiene et al. (2023)
9	Integrated: barley (4) (PWE + M, US + EH, M + US); BSG (2) (M + US, M + EH); barley bran (2) (US + EH, M + US); wheat bran (1) (CP + EH)		27.03	20.81	19.51	2.16–62.43	25.09	Anis et al. (2024), Cao et al. (2023), Chen et al. (2024), Falade et al. (2025), Fareed et al. (2025), Li et al. (2025), Liu et al. (2021), Özen et al. (2024), Xi et al. (2023)
2	Integrated: barley (1) (M + US + EH); barley bran (1) (M + US + EH)		65.85	65.85	3.61	63.3–68.4	2.55	Li et al. (2021)
42	Linear: oat (1) (US); barley (3) (M, US); wheat (5) (M, US, SFE); maize (4) (US, SFE, EH), sorghum (1) (M); BSG (4) (PEF, OH, US, M); oat bran (5) (US, EH, F); wheat bran (17) (M, US, SFE, EH, OH); rye bran (2) (EH, SFE)	Phytochemical	2.97	0.99	6.02	0.03–31.36	1.75	Al‐Hilphy et al. (2020), Álvarez‐Romero et al. (2023), Argun (2022), Arslan et al. (2020), Baah et al. (2025), Bautista‐Expósito et al. (2020), Bonifácio‐Lopes et al. (2022), Burlini et al. (2020), Călinoiu and Vodnar (2020), Cavallero et al. (2025), Cengiz et al. (2021), Chen et al. (2020, 2022), Cherif et al. (2020), Guerrini et al. (2020), Habuš et al. (2021), Iadecola et al. (2022), Ibrahim et al. (2025), Kirusnaruban et al. (2025), Kumar et al. (2025), Liu, Wu, et al. (2024), López‐Linares et al. (2021), Malenica et al. (2025), Martín‐García et al. (2020), Milićević et al. (2021), NEMES et al. (2024), Radenkovs et al. (2021), Razem et al. (2025), Rebolleda et al. (2020), Sahu et al. (2021), Spaggiari et al. (2023)
7	Integrated: barley (1) (US + F); maize (2) (M + US); BSG (1) (M + US); wheat bran (3) (M + US, US + F, M + F)		2.17	0.60	4.41	0.05–12.13	0.84	Boateng et al. (2023), Cavallero et al. (2025), García‐Ortíz et al. (2023), Nemes et al. (2025), Wang et al. (2024), Zago et al. (2022)
6	Linear: oat (1) (US); barley (3) (PWE, US, M); maize (1) (PFE); oat bran (1) (M)	Protein	14.13	8.57	16.61	1.24–43.00	18.91	Bai et al. (2022), He et al. (2020), Perez‐Vega et al. (2023), Sukop et al. (2025)
		Fiber/Prebiotic	59.90	77.98	31.75	12.23–84.27	40.80	
1	Linear: wheat bran (EH)	Protein	15.80	—	—	—	—	Ferri et al. (2020)
		Phytochemical	1.20	—	—	—	—	
5	Linear: oat (1) (US); oat bran (2) (US, F); barley bran (1) (US); wheat bran (1) (F)	Fiber/Prebiotic	44.18	24.72	27.63	23.57–80.75	42.57	Grgić et al. (2023), Ibrahim, Nadeem, Sultan, et al. (2024), Li et al. (2024), Sun et al. (2025)
		Phytochemical	2.94	0.75	5.46	0.09–12.7	0.50	
2	Integrated: barley (1) (M + EH); wheat bran (1) (SWE + EH)	Fiber/prebiotic	71.33	71.33	4.07	68.45–74.2	2.88	Fall et al. (2025), Rudjito et al. (2024)
		Phytochemical	0.82	0.82	1.10	0.04–1.6	0.78	
1	Barley (M + EH + F)	Fiber/Prebiotic	57.24	—	—	—	—	Fall et al. (2025)
		Phytochemical	0.03	—	—	—	—	
6	Linear: barley (1) (M); BSG (1) (US); wheat bran (4) (EH, SWE)	Protein	14.36	9.28	9.98	7–31.15	11.53	Alonso‐Riaño et al. (2020), Bhattacharya et al. (2020), Hong et al. (2022), Martín‐Diana et al. (2021), Yilmaz‐Turan et al. (2020), Zhuang et al. (2024)
		Fiber/Prebiotic	25.08	21.52	15.50	5.35–45.00	21.12	
		Phytochemical	1.21	0.82	1.25	0.06–3.39	1.29	
4	Integrated: wheat (1) (M + F); maize (1) (M + US); wheat bran (2) (EH + F, SWE + EH)	Protein	17.13	16.24	6.92	10.8–25.25	9.97	Garcia‐Ortiz et al. (2023), Hassan et al. (2025), Li et al. (2020), Pan et al. (2025)
		Fiber/Prebiotic	24.56	17.06	22.32	6.88–57.24	13.98	
		Phytochemical	0.88	0.64	0.91	0.06–2.18	0.58	
1	Integrated: wheat (US + EH + F)	Protein	13.99	—	—	—	—	Pan et al. (2025)
		Fiber/Prebiotic	66.20	—	—	—	—	
		Phytochemical	0.06	—	—	—	—	

*Note*: Yield values were collected from the original studies and standardized to a uniform unit (% w/w). Although some methodological heterogeneity exists, including differences in cereal type, pretreatment, extraction conditions, and product purity, only yield data were aggregated by processing method and biomass type. Statistical summaries include mean, median, standard deviation (SD), minimum–maximum range, and interquartile range (IQR). Linear and integrated strategies represent single‐ and multi‐step fractionation strategies, respectively.

Abbreviations: BSG, brewer's spent grain; CP, cold plasma; EH, enzymatic hydrolysis; F, fermentation; IQR, interquartile range; M, microwave; OH, ohmic heating; PEF, pulsed electric field; PWE, pressurized CO_2_/H_2_O extraction; SD, standard deviation; SFE, supercritical fluid/CO_2_ extraction; SWE, subcritical water extraction; US, ultrasound.

##### 3.1.1.1 Protein

Protein recovery applying linear fractionation methods achieved the highest average extraction yields, particularly from oat, wheat, and sorghum. The mean yield was 57.86% w/w, with a median of 55.80% w/w, indicating a relatively balanced distribution. Nonetheless, the extraction values showed considerable heterogeneity, ranging from 33.00% to 88.66% w/w, with an IQR of 16.65. This variation can be attributed to differences in grain quality, functional composition, and processing techniques. For example, US‐assisted protein recovery from wheat achieved up to 88.66% w/w (Xie et al. 2023), whereas SFE on oat yielded 55.8% w/w (Gaber et al. 2025). In contrast, integrated methods (e.g., M + EH, M + US) applied to wheat and maize resulted in a lower mean protein recovery of 30.61% w/w, with a higher SD (27.50) and a broader range (11.16%–50.05% w/w), whereas strategies such as M + F, PFE, PWE, SWE + EH, and US + EH + F generally yielded even lower protein amounts. Moreover, bran‐ and BSG‐based linear extractions yielded significantly less protein (mean: 15.33% w/w), suggesting inefficient extraction of low‐protein substrates or different processing goals (e.g., prioritizing fiber or polyphenols). Although sorghum was barely investigated, it showed high protein recovery via EH‐assisted extraction (64.25% w/w), with efficiencies found up to 98.75% (Xiao et al. 2024), highlighting its underexplored potential for protein valorization. The relatively wide range of protein recovery reflects differences in both processing strategy and feedstock characteristics. The high recovery reported for wheat using US‐assisted processing contrasts with the lower mean recovery observed for integrated bran and BSG strategies, where additional fractionation steps and matrix complexity can affect protein recovery. Thus, the higher numerical recovery of individual linear processes should not be interpreted as evidence of higher overall process performance without considering purity and co‐product recovery.

##### 3.1.1.2 Fiber

The effect of the fiber content extraction process was evident in significant variation between the feedstock biomass and the methods used. Linear processing of oat, barley bran, wheat bran, and maize bran achieved a mean yield of 25.74% w/w (median: 13.80% w/w; range: 1.45%–90.00% w/w; SD: 32.97). However, integrated strategies yielded slightly higher mean values of 27.03% w/w (median: 20.81% w/w), with lowered variability (SD: 19.51; IQR: 25.09), suggesting improved stability and sequential process stages. Notably, triple‐step integrations, such as M + US + EH applied to barley and barley bran, achieved high fiber yields (mean: 65.85% w/w; range: 63.3%–68.4% w/w; SD: 3.61) (Li et al. 2021), indicating strong potential for maximizing dietary fiber recovery. These methods are especially effective for β‐glucan‐rich matrices (e.g., oat and barley) (He et al. 2020; Ibrahim, Nadeem, Khalid, et al. 2024; Ibrahim, Nadeem, Sultan, et al. 2024). BSG also exhibited promising potential, and M + US treatments delivered soluble dietary fiber yields up to 39.74% w/w (Falade et al. 2025), highlighting the suitability of BSG for circular bioingredient valorization. The broad variation in fiber recovery also indicates differences in biomass composition and fraction definition. Integrated physical–enzymatic strategies can provide substantially higher fiber recovery in selected barley systems, but this advantage may involve additional process and separation steps. Consequently, recovery gains should be evaluated against processing time, enzyme requirements, and downstream separation rather than considered independently.

##### 3.1.1.3 Phytochemicals

Phytochemical compounds are more sensitive and often present at lower concentrations than proteins or fibers, so their extraction yields are typically lower, underscoring the importance of maximizing recovery. In accordance with previous studies, such as linear techniques, yielded an average of 2.97% w/w (median: 0.99% w/w; range: 0.03%–31.36% w/w; SD: 6.02) and exhibited a variance due to the extraction method (e.g., SFE, US), feedstock substrate, and sensitivity of compounds. Maize bran revealed greater phytochemical extraction employing US (31.36% w/w) and SFE (19.98% w/w) (Burlini et al. 2020; Kumar et al. 2025). Integrated methods generally yielded lower phytochemical concentrations (mean: 2.17% w/w; median: 0.60% w/w; IQR: 0.84% w/w), likely due to degradation during extended processing or insufficient targeting of sensitive bioactives. The substantially wider variability observed for phytochemical recovery indicates the stronger dependence of extraction performance on solvent polarity, temperature, matrix accessibility, and extraction selectivity. As a consequence, the highest reported yield should not automatically be considered the most industrially attractive outcome, particularly when high solvent consumption, solvent recovery, or downstream concentration is required.

##### 3.1.1.4 Oils or Lipophilic

The recovery of oils or lipophilic fractions from oat, wheat, and maize bran or germ is strongly influenced by the target fraction, feedstock, and processing method. When specifically extracted as oil‐rich fractions or employing defatting, they consistently achieved higher yields than general lipophilic extractions. For instance, US‐assisted three‐phase partitioning of oat groats produced an oil‐rich fraction of 8.56% (Perez‐Vega et al. 2023), whereas the SFE of wheat bran yielded polyunsaturated fatty acid‐rich lipid extracts at up to 1.6% (Argun 2022). The process of defatting applied to wheat germ and flour using SFE steadily enhanced oil recovery by 5.2%–9.6% compared with conventional hexane extraction while effectively preserving antioxidants such as tocopherols and phenolics (Sahu et al. 2021). Multi‐step integrated strategies combining US and EH improved the recovery of phenolic fractions and subsequent SFE of lipophilic fractions from maize germ, yielding substantial ferulic acid and unsaponifiable lipids (Burlini et al. 2020).

#### 3.1.2 Effect of Composition on Fractionation

In addition to processing conditions, the intrinsic physical and chemical properties of cereal biomass are key determinants of fractionation efficiency, influencing both extraction yields and responses to mechanical, thermal, or enzymatic treatments. A clear pattern from the studies is that feedstock characteristics significantly influence extraction, affecting yields and processing requirements. The differences in recovery among different cereals, such as oat, wheat, and maize can be attributed partly to differences in kernel composition, grain hardness, cell‐wall architecture, starch–protein organization, lipid distribution, phenolic localization, and endogenous enzyme activity. Oat matrices contain relatively accessible soluble β‐glucans and cell‐wall‐associated phenolic compounds, which can facilitate aqueous and enzymatic recovery. In contrast, stronger protein–starch associations and heterogeneous localization of bound phenolics in wheat and maize may increase mass‐transfer resistance and require more intensive pretreatment. Nonetheless, recovery differences should not be interpreted as an inherent superiority of one cereal, because cultivar, anatomical fraction, particle size, moisture, pretreatment, and target compound also strongly influence the observed response. Thus, fractionation efficiency results from the interaction between biomass composition and process conditions rather than from cereal type alone.

Particularly in non‐conventional fractionation, lipid‐linked protein–fiber complexes and comparatively flexible cell‐wall structures in certain cereals facilitated higher recoveries. Oat substrates offered high protein (SFE: 55.8% w/w) and fiber (up to 80.75% w/w with US and 47.6% w/w with EH) recoveries (Gaber et al. 2025; Ibrahim, Nadeem, Sultan, et al. 2024; Sargautis and Kince 2023). Furthermore, US‐assisted and F processes resulted in solid fiber extractions (24.65%–24.72% w/w) from bran, as well as adequate phytochemical levels (Grgić et al. 2023; Li et al. 2024). When the integrated processes, such as M + US + EH, were applied, fiber extraction from barley reached up to 68.4% w/w (Li et al. 2021). Wheat bran yielded considerable fiber (74.2% w/w) with SWE + EH and reasonable phytochemical extraction (Rudjito et al. 2024). Meanwhile, maize responded well to PEF for dual recovery of protein (43% w/w) and fiber (26.7% w/w) (Sukop et al. 2025), whereas EH produced fiber content of 13.8% w/w (Jiang et al. 2025), although protein yield under integrated processing remained low. Collectively, these observations indicate the need to customize process design to biomass‐specific microstructures and compositional profiles to optimize recovery efficiency.

The feedstock–process interaction is also affected by particle size, moisture content, cultivar, and maturity. Figure 4 illustrates the relationship between biomass composition and key feedstock characteristics and their influence on fractionation efficiency. Specific surface area and diffusion distance are critical parameters for matrix accessibility, and their relationship is non‐proportional, whereas reduced particle size generally decreases diffusion distances. Moisture affects matrix plasticization, molecular mobility, solvent penetration, and dielectric or acoustic energy transfer. These effects are also important for lipid recovery because endogenous lipases can promote lipid hydrolysis; uncontrolled moisture in wheat bran has been associated with free fatty acids reaching approximately 15% as oleic acid (Rebolleda et al. 2020). However, excessive comminution can increase energy demand, promote agglomeration, and complicate solid–liquid separation. Cryogenic micronization of whole‐wheat bran followed by high‐intensity US increased phenolic accessibility and reduced polyphenol oxidase activity by up to 93% (Habuš et al. 2021). Conversely, direct M treatment of intact wheat kernels demonstrated that extensive fractionation is not always necessary when volumetric energy transfer is sufficiently rapid (Kirusnaruban et al. 2025). Cultivar and maturity also influence grain hardness, starch and protein organization, lipid content, β‐glucan concentration, cell‐wall composition, and the distribution of free and bound phytochemicals. Consequently, comparisons among cereals should preferably use equivalent anatomical fractions and normalized analytical bases, whereas process conditions should be re‐optimized when transferring a fractionation process between feedstocks.

**FIGURE 4:**
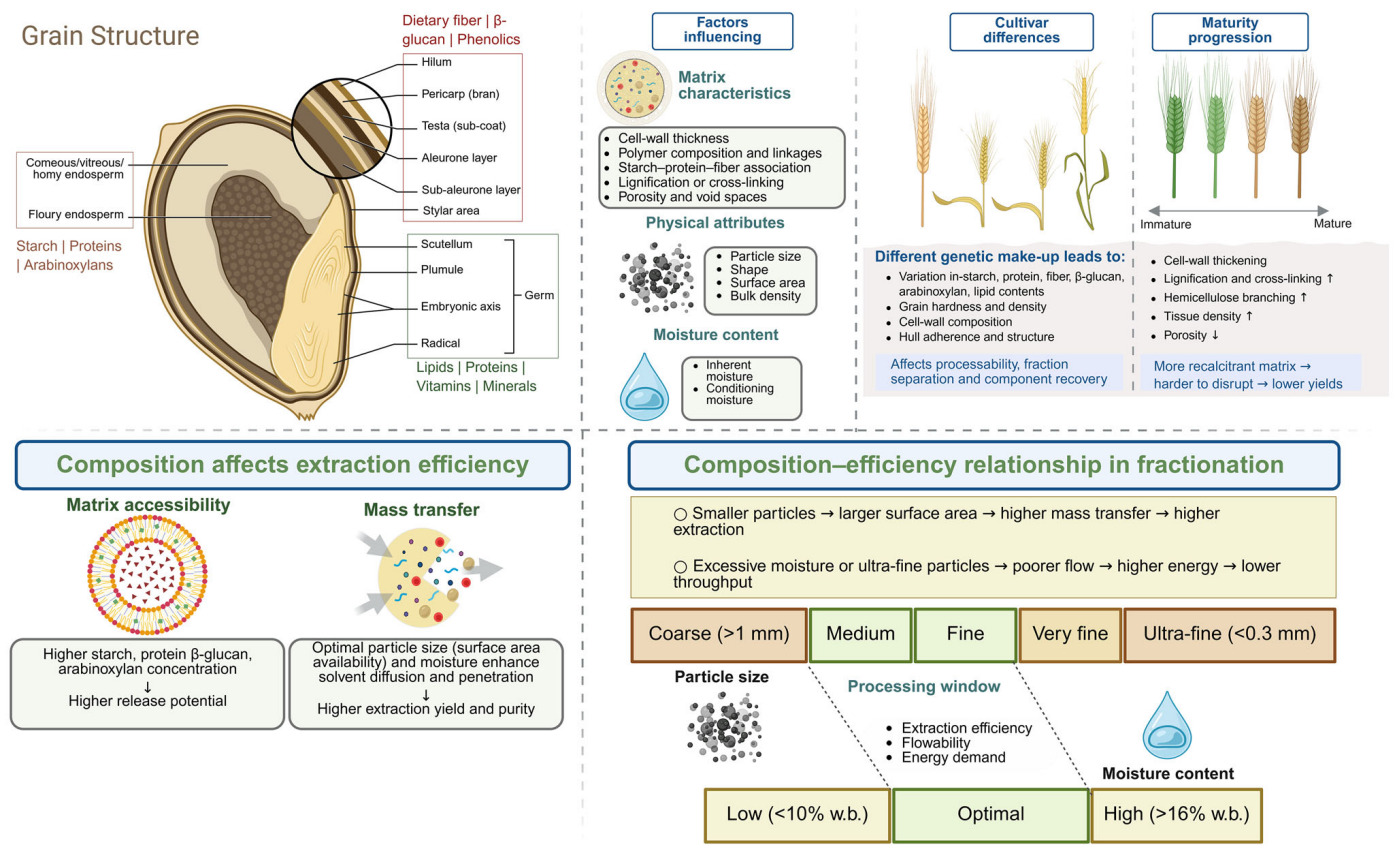
Biomass composition and structural attributes governing fractionation efficiency.

#### 3.1.3 Interaction of Fractionation Strategies

The separation of compounds and the development of functional biopolymers depend on the chosen fractionation strategy, which ultimately determines the structural integrity of the biomass and the retention of associated bioactive substances in the final product. This interaction determines whether inherent macromolecular assemblies are preserved, partially modified, or fully disassembled during processing, thereby directly influencing the functionality of the extracted fractions. Different fractionation strategies can be conceptualized as a continuum ranging from preservation of intrinsic macromolecular complexes to complete molecular isolation, with hybrid systems positioned between these extremes. In this framework, performance cannot be evaluated solely based on yield or purity. Instead, it depends on how effectively each strategy balances structural complexity with functional retention—bioactivity and techno‐functional properties. Figure 5 systematically illustrates this continuum by distinguishing synergistic fractionation, purification‐driven isolation, and hybrid fractionation according to their degree of structural preservation and the resulting product characteristics, including compositional complexity, bioactivity retention, purity, and techno‐functional performance.

**FIGURE 5:**
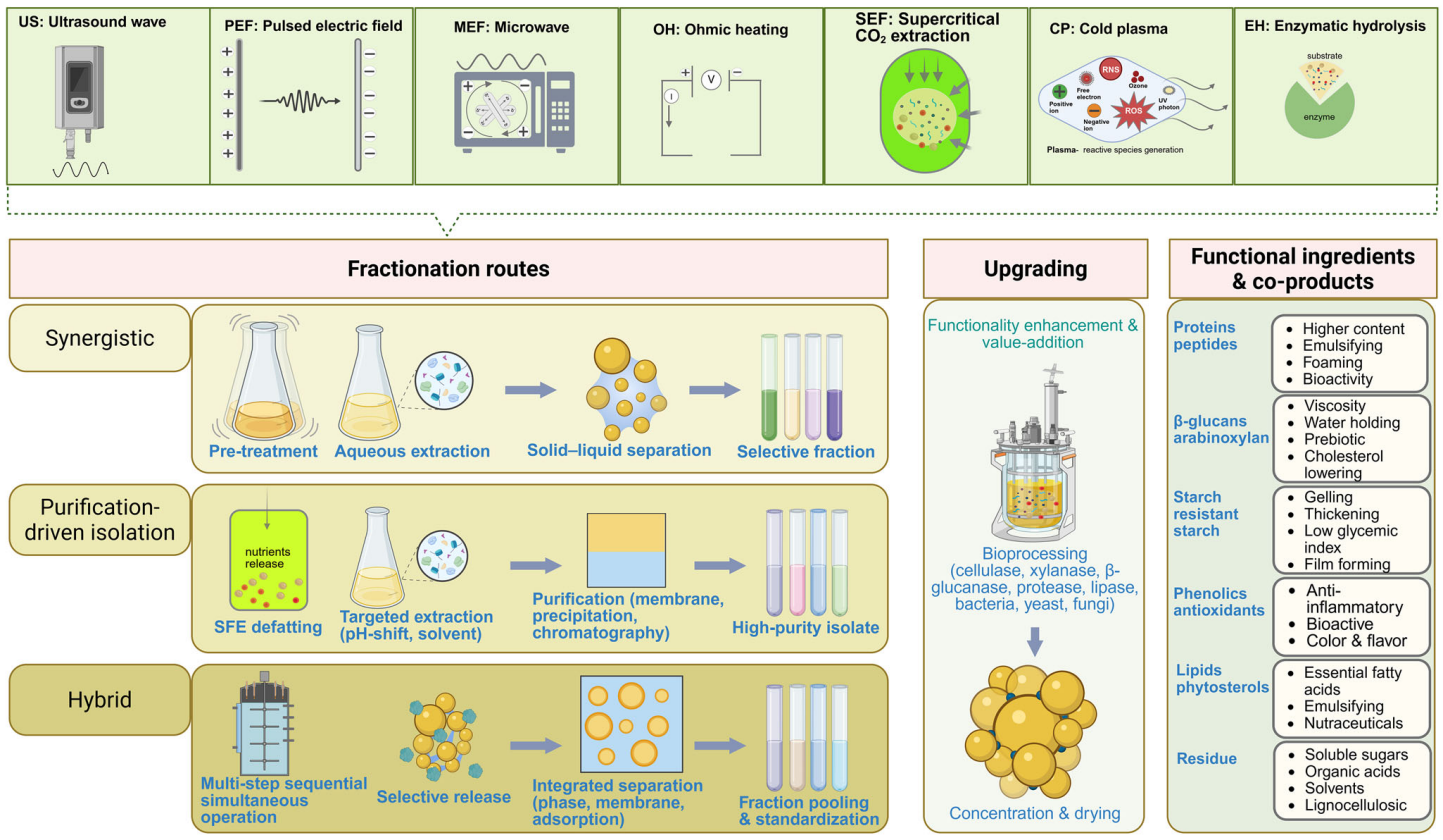
Conceptual processing arrangements and interactions for intensified fractionation.

##### 3.1.3.1 Synergistic Fractionation

The separation of compounds and the development of functional biopolymers depend on fractionation options. The synergistic recovery strategy enhances antioxidant capacity in protein–fiber complexes by protecting the natural polyphenols bound to the macromolecular matrix. This technique effectively prevents the separation of proteins from polysaccharides, preserving the natural synergistic network, which often contains bound phenols. Polyphenolic–protein–polysaccharide complexes are extracted using pressurized water, thereby linking high polyphenol retention to significantly improved free‐radical scavenging abilities (Wu et al. 2020). The simultaneous co‐extraction of proteins and soluble saccharides using thermal and homogenization aids yields functional biopolymers with strong lipid‐lowering and colonic F properties without the need for severe isolation steps (Chen et al. 2023). This is also demonstrated by a sequential aqueous extraction, which yields natural protein–polysaccharide fractions with higher emulsifying properties because the protective fiber network remains intact (Li et al. 2023). Moreover, co‐extraction with ultrasonic assistance modifies the structural dynamics, creating strong, naturally occurring composite materials that perform better than separate emulsifiers (Zie et al. 2026). The use of supercritical CO_2_ under optimal conditions, guided by whole‐recovery principles, has the potential to efficiently extract antioxidant‐rich lipid fractions from rye bran (Povilaitis and Venskutonis 2015) and barley pearling flour (Temelli et al. 2013), underscoring the importance of preserving matrix coherence for functional performance. The antioxidant response can be enhanced by the rapid release of phenolic compounds, as demonstrated by US‐assisted treatment (Cengiz et al. 2021). M‐assisted extraction produced FRAP activity of approximately 4.63 mg Fe^2^^+^/g and ORAC activity of approximately 48.98 µmol TE/g, whereas hydrothermal M treatment produced total phenolic content of approximately 5.41–5.77 mg GAE/g dry weight (Cavallero et al. 2025; Kirusnaruban et al. 2025). For a combined M–EH–US strategy applied to oat‐derived β‐glucan fractions, ABTS, hydroxyl‐radical, and superoxide‐scavenging activities reached approximately 84.36%, 68.32%, and 54.32%, respectively, together with 93.42% β‐glucan purity (Cao et al. 2023). Hence, a more thorough assessment of synergistic performance should compare the combined process with each individual operation under equivalent conditions. The synergistic gain should be expressed as a relative improvement in antioxidant activity or specific phenolic recovery, together with extraction yield and energy input. Such normalization would help distinguish genuine process synergy from simple additive effects arising from longer residence time or higher energy input.

##### 3.1.3.2 Purification‐Driven Isolation

On the other hand, fraction purification focuses on isolating highly pure components through sequences of alkaline solubilization, isoelectric precipitation, dialysis, and drying. Although these traditional methods provide precise control over the end product's molecular weight and composition, they pose the risk of losing the smaller bioactive molecules that provide it antioxidant activity. Protein solubility and foaming capability are modified when protein–polysaccharide complexes are formed using alkaline extraction and isoelectric precipitation. Notably, the pH selected during precipitation significantly affects these alterations (He et al. 2025). Fractional ethanol precipitation followed by rigorous column chromatography is used to isolate highly specific and pure protein–bound polysaccharides (Nuerxiati et al. 2022). These purified fractions exhibited strong thermal stability; however, the pronounced separation of bound natural compounds suggests that achieving high antioxidant activity depends solely on the fundamental composition of the purified complex itself, such as specific uronic acid content, rather than on the synergistic enhancement typically provided by co‐extracted polyphenols. This is also supported by the separation of protein–polysaccharide conjugates by sequential acid‐alkaline and enzymatic treatments followed by precipitation, where the resulting antioxidant capacity was completely contingent on the closely bound phlorotannins and specifically maintained sulfated fucans (Bojorges et al. 2025).

However, increasing purity can remove undesirable compounds, although it can also eliminate naturally occurring compounds that contribute to antioxidant activity and techno‐functional performance. Cereal matrices are intrinsically multicomponent systems, and phenolics can interact with proteins, polysaccharides, lipids, and minerals. As a result, intensive purification may disrupt matrix interactions that influence antioxidant stability, release during digestion, or physiological accessibility. For functional foods, the optimum ingredient may be a standardized multifunctional fraction rather than the highest‐purity isolated component. For instance, dry oat fractionation can generate protein concentrates with approximately 44.2%–55.8% protein while retaining favorable techno‐functional properties, whereas more intensive aqueous processing can increase protein purity to around 84.2%–86.5% but may reduce overall recovery to almost 44.8% (Gaber et al. 2025). Moreover, around 35.9%–47.6% of protein can remain in the fiber‐rich residue after aqueous extraction, indicating that increasing purity may be accompanied by lower biomass utilization (Sargautis and Kince 2023). Similarly, for bioactive fraction recovery, removal of proteins and polysaccharides may increase analytical phenolic purity while reducing matrix protection or altering release kinetics. Therefore, purification should be selected according to the intended application. High‐purity isolates may be justified for standardized nutraceutical applications, whereas food formulations may benefit from moderately purified fractions that retain synergistic proteins, fibers, phenolics, and lipids. Accordingly, future studies should focus on both chemical purity and biological or techno‐functional performance after each purification stage to determine whether additional purification provides a meaningful functional benefit.

##### 3.1.3.3 Hybrid and Phase‐Separation

In advanced separation techniques, phase partitioning and novel solvent extraction procedures can be the best choice to overcome the process complexity and yield balance for isolating multiple fractions simultaneously without the severe degradation observed in conventional precipitation. Deep eutectic solvents (DES) or complex phase separations can selectively partition proteins while maintaining mild conditions that theoretically protect bioactive structures (Akyüz et al. 2024). Hybrid protein–polysaccharide extracts revealed that combining a simple ultrasonic pretreatment with specific pH‐shifting resulted in a crude form of phase separation that significantly increased the release of intracellular phenolic compounds, thereby increasing the extract's antioxidant capacity (Vega‐Gómez et al. 2024). Although this technology provides a distinct way to customize the exact protein‐to‐fiber ratio (Shi et al. 2024), its translation into food applications requires significant adjustment of analytical validation and safety assessment to ensure food‐grade quality, alongside compliance with regulatory frameworks such as GRAS (United States) and novel food regulations (EU), which remain key barriers to industrial implementation. However, the industrial feasibility of DES depends strongly on solvent recovery, recyclability, toxicity, and economic feasibility. DES have attracted interest because their components can be selected from relatively benign compounds such as organic acids, sugars, amino acids, and polyols, whereas their low vapor pressure and responsive polarity provide opportunities for extracting phenolics and other polar compounds. Nevertheless, their classification as green should not be based only on low volatility or the origin of their components. High viscosity can decrease mass transfer and increase pumping requirements, whereas the recovery of non‐volatile DES from product streams is more challenging than recovery of conventional solvents such as ethanol or CO_2_. In addition, water content can substantially modify DES physicochemical properties and extraction performance. Therefore, solvent recyclability should be demonstrated experimentally rather than assumed. Recyclability should be evaluated over repeated extraction cycles by monitoring extraction performance, solvent composition, accumulation of impurities, and product quality. Toxicological assessment and residual‐solvent control are also important for food applications, particularly because the safety profile of a DES depends on its individual components, composition, concentration, and potential interactions. The environmental and economic assessment should additionally consider DES preparation, component production, water consumption, energy requirements, solvent purification, recycling, and end‐of‐life management. These factors become particularly important at industrial scale because solvent recovery and downstream purification can contribute substantially to capital and operating costs. By comparison, SFE systems can recycle more than 95% of CO_2_ in closed‐loop configurations, although their pressure‐rated equipment results in high CAPEX (Rebolleda et al. 2020). Thus, the economic attractiveness of DES‐based extraction will depend on whether solvent recycling and product recovery can be integrated without diminishing the advantages of low volatility and adjustable selectivity. At present, limited long‐term solvent‐reuse data, incomplete toxicological characterization for many DES formulations, high viscosity, uncertain downstream solvent recovery, and insufficient pilot‐scale validation remain major barriers to industrial adoption.

## 4 Functional, Nutritional, and Biological Evaluation of Cereal Fractions

In addition to compositional recovery, the functional performance of extracted cereal fractions is pivotal in determining their potential for use in food matrix design. Numerous studies have evaluated techno‐functional and biofunctional properties of protein–fiber–polyphenol‐rich fractions derived from cereals and side streams. These properties can be differentiated into three interconnected categories: functional, nutritional, and biological properties. Functional properties describe the technological behavior of the recovered fractions during processing and storage. Nutritional properties relate to protein digestibility, amino acid availability, starch digestibility, and glycemic response. Biological properties encompass antioxidants, enzyme‐inhibitory, prebiotic, and other bioactive effects. Their distinction and relevance to cereal fraction utilization are summarized in Table 4. The native molecular conformation of ingredients is eventually fractured by harsh processing conditions. High‐moisture extrusion employs intense shear and thermal stress in order to structure proteins into fibrous networks; however, this severity often compromises moisture retention and lipid distribution, reducing sensory tenderness (Gulzar et al. 2025). Although the antioxidant properties of extracted polyphenols and pigments are effectively preserved by moderate green extraction methods, they usually have extended contact durations that may adversely affect the target compound's functional efficiency (Ameer et al. 2017; de Souza Mesquita et al. 2021). Therefore, the value of cereal fractions should not be assessed solely according to extraction yield or compositional recovery, but also according to how processing‐induced structural changes influence their technological functionality, nutritional quality, and biological activity.

**TABLE 4:** Key characteristics and relevance of process‐derived ingredients.

Property	Assessment focus	Indicators	Food relevance
**Functional**	Physicochemical behavior during processing and formulation	Solubility, WHC, OHC, emulsification, foaming, gelation, viscosity, pasting behavior, particle size, and thermal stability	Texture, stability, processing performance, and product formulation
**Nutritional**	Nutrient composition, accessibility, and digestion	Quality, availability, digestibility, characteristics, antinutritional factors, glycemic response	Nutrient utilization, satiety, and metabolic response
**Biological**	Physiological or biochemical activity	Antioxidants (DPPH, FRAP, ORAC), antimicrobial, enzyme‐inhibitory, anti‐inflammatory, and prebiotic effects	Potential health‐related functionality and bioactivity

Abbreviations: DPPH, 2,2‐diphenyl‐1‐picrylhydrazyl radical scavenging assay; FRAP, ferric reducing antioxidant power assay; OHC, oil‐holding capacity; ORAC, oxygen radical absorbance capacity; WHC, water‐holding capacity.

### 4.1 Functional Properties and Food‐Product Performance

Ideal feedstocks for nutrient‐rich complex foods are those exhibiting high solubility, emulsifying capacity, digestibility, and bioactivity features most commonly found in oat, barley, wheat bran, and selected sorghum varieties (Grgić et al. 2023; Li et al. 2021; Liu et al. 2021; Xiao et al. 2024). From a technological perspective, these properties should be evaluated in relation to the requirements of the intended food matrix rather than as isolated measurements.

Protein‐rich cereal fractions demonstrated favorable techno‐functional properties such as good foaming capacity (up to 126.17%), oil‐holding capacity (OHC: 6.99 g/g), and water‐holding capacity (WHC: up to 14.07 g/g), in several feedstocks (Anis et al. 2024; Ibrahim, Nadeem, Sultan, et al. 2024; Xiao et al. 2024). These characteristics render the products suitable for applications in plant‐based meat analogs, emulsified systems, and dairy alternatives (Gaber et al. 2025; Xie et al. 2023). Improved solubility (up to 89.03%) and strong emulsifying/foaming behavior were particularly notable in extracts from oat, barley, and wheat bran processed using hydrothermal and enzymatic treatments (Jiang et al. 2025; Li et al. 2021; Liu et al. 2021). The practical relevance of these measured properties depends strongly on the intended food matrix. High WHC and OHC can improve moisture and lipid retention in structured systems, whereas excessive hydration, swelling, or aggregation may be unfavorable in low‐viscosity beverages. For example, M‐modified highland barley bran showed an increase in the soluble‐to‐insoluble dietary fiber ratio from 3.68% to 15.47%, accompanied by increases in WHC (2.55–4.78 g/g) and swelling power (2.01–4.56 mL/g); further, F increased their activity up to around 5.89 g/g and 6.06 mL/g, respectively (Fall et al. 2025). Similarly, oat protein concentrates obtained through enzymatic starch hydrolysis exhibited high water‐binding (2.83–3.0 g/g) and OHC (2.16–2.21 g/g) (Sargautis and Kince 2023). Such hydration characteristics favor incorporation into cereal‐based food products, comminuted meat systems, and structured plant‐based meat analogues, where moisture retention and matrix stabilization are desirable. Their use in fluid systems may require secondary stabilization to control swelling, aggregation, and sedimentation.

Starch‐based fractions exhibited relevant gelling (Li et al. 2020), viscosity (Li et al. 2021), and crystallinity (up to 47.54%) (Ibrahim, Nadeem, Khalid, et al. 2024) behavior, which are important for structuring low‐glycemic or functional bakery and snack products. Moreover, cation exchange capacities (up to 30.46 mg/g), bile salt adsorption, and α‐amylase inhibition were observed in several insoluble fractions, indicating potential for glycemic response modulation and lipid‐binding functionalities (Li et al. 2025; Xi et al. 2023). Although the latter properties have nutritional and biological implications, they also influence the functionality of insoluble fractions by affecting hydration, matrix interactions, and the retention of other food components.

The relationship between processing and functionality is particularly evident in protein solubility and interfacial behavior. Controlled physical disruption and EH can expose buried peptide groups and increase protein solubilization, whereas excessive unfolding followed by intermolecular reassociation may promote aggregation and reduce dispersibility. US‐assisted enzymolysis of defatted wheat germ increased total soluble protein by approximately 139.72% and polypeptide content by 31.78%, indicating that acoustic cavitation can facilitate structural opening and subsequent enzymatic cleavage (Pan et al. 2025). In contrast, oat protein fractions obtained through enzymatic starch depolymerization exhibited low protein solubility indices (<7%) across pH 3–9 and poor foaming properties (<8% initial foamability, with rapid collapse within 10 min), which limits their application as primary foaming or emulsifying agents in fluid and aerated systems (Sargautis and Kince 2023). Such fractions may instead be more suitable for high‐solid formulations such as nutritional bars and extruded snacks, where controlled aggregation and low dispersibility can be advantageous for maintaining product structure. Thus, functional measurements should be interpreted in terms of their intended formulation role. High WHC can suppress syneresis during thermal processing and refrigerated storage, whereas high OHC can contribute to lipid and flavor retention during processing. Conversely, poor solubility and unstable foaming may restrict application in beverages and aerated products but may be acceptable, or even desirable, in dense solid matrices. This property‐to‐application relationship is essential for determining whether a recovered cereal fraction can function as a structural ingredient, stabilizer, emulsifier, or texturizing component.

### 4.2 Nutritional Properties and Digestibility

The nutritional performance of cereal fractions is strongly influenced by processing‐induced changes in protein structure, amino acid distribution, peptide accessibility, and starch organization. These changes can either improve nutrient accessibility or generate structural modifications that restrict digestion. M treatment can also induce protein unfolding through rapid dielectric heating, exposing previously buried scissile bonds to pepsin and pancreatin. Subsequent fermentative proteolysis can further increase soluble peptides; however, prolonged F may over‐hydrolyze functional peptides into free amino acids, thereby changing both nutritional and biofunctional performance. Processing can also alter amino acid availability and distribution among recovered fractions. In US‐pretreated and *Bacillus subtilis*‐fermented defatted wheat germ, lysine increased from 258.54 to 706.48 mg/L, corresponding to a 173.26% increase, whereas total amino acids reached 1112.14 mg/L and the essential and total amino acids ratio increased (68.74%–80.73%) (Pan et al. 2025). These results demonstrate the potential of targeted microbial bioconversion to improve the nutritional quality of cereal protein fractions by modifying their amino acid distribution. However, fractionation itself can redistribute individual amino acids between processing streams. In enzymatic separation of oat flakes, the insoluble fiber fraction became enriched in arginine and lysine, whereas the recovered protein concentrate exhibited increased cysteine (+27.3%) and methionine (+28.3%) but a modest reduction in lysine to approximately 3.44–3.48 g/100 g protein from 3.71 g/100 g (Sargautis and Kince 2023). Therefore, amino acid quality should be evaluated at the fraction level rather than inferred only from total protein recovery.

Low‐molecular‐weight peptide generation provides an additional connection between processing and nutritional accessibility. Membrane separation of papain‐hydrolyzed wheat germ albumin produced a <3 kDa peptide fraction corresponding to approximately 50.05% of the hydrolysate mass. This fraction exhibited stronger antioxidant activity than the larger molecular‐weight fractions, with a DPPH EC_50_ of 1.78 mg/mL, which decreased to 1.46 mg/mL, together with an IC_50_ of 4.08 mg/mL against superoxide radicals (Tian et al. 2022). Similarly, <3 kDa fractions from US‐pretreated and fermented germ exhibited the highest DPPH (18.53%), ABTS^•+^ (40.54%), and FRAP (0.56 mM FeSO_4_·7H_2_O) activities (Pan et al. 2025). These findings suggest that controlled hydrolysis can generate peptides with enhanced molecular mobility and solvent accessibility. However, excessive cleavage may reduce activity because specific peptide sequences and combinations of amino acid residues can contribute to bioactivity. Thus, controlled production of approximately 1–3 kDa peptides may be more desirable than indiscriminate extensive hydrolysis.

Starch digestibility is similarly dependent on the extent and nature of structural modification. Digestibility and metabolic response results were reported for several fractions, including improvements in the starch hydrolysis index, reduced estimated glycemic index (GI), and enhanced bioavailability (Baah et al. 2025; Fall et al. 2025; Xiao et al. 2024). These findings indicate the potential suitability of selected cereal‐derived ingredients for low‐GI foods, diabetic formulations, or sustained‐energy‐release products. Processing that disrupts starch order can increase enzyme accessibility and accelerate hydrolysis, whereas treatments that promote resistant starch formation can reduce digestibility and estimated GI. M‐assisted restructuring of cereal flour under moisture‐restricted conditions increased resistant starch to approximately 55% and reduced the estimated GI from approximately 78.08%–56.57% in the investigated wheat system (Baah et al. 2025). Hence, the same processing strategy may produce contrasting nutritional outcomes depending on treatment severity, moisture conditions, and the structural organization of the resulting starch fraction. The glycemic response of processed cereal fractions is further influenced by interactions among starch, dietary fiber, and released phenolics. US and M treatments can disrupt starch organization and increase the accessibility of α‐amylase and amyloglucosidase. In US‐pretreatment, the FTIR band shift from approximately 1028 to 1038 cm^−1^, together with reduced band intensity, indicated disruption of short‐range starch order and increased structural accessibility (Pan et al. 2025). Conversely, increased resistant starch formation can reduce starch hydrolysis and estimated GI, as demonstrated by the wheat system described above (Baah et al. 2025).

In parallel, processing‐induced release of phenolics may provide an additional mechanism for modulating carbohydrate digestion. M and cellulase treatment of barley released approximately 64.23%–68.30% of bound phenolics during simulated colonic digestion, with ferulic acid reaching approximately 701.30 µg/g. Released phenolics can interact with α‐amylase and α‐glucosidase through hydrogen bonding and hydrophobic interactions, potentially reducing carbohydrate hydrolysis. Increased soluble dietary fiber can further increase matrix hydration and viscosity, restricting enzyme diffusion and delaying glucose transport (Liu, Wu, et al. 2024). Therefore, the final glycemic response depends on the combined effects of starch structural organization, resistant starch formation, fiber composition, phenolic accessibility, and protein–polyphenol interactions rather than starch content alone.

### 4.3 Biological Properties and Health‐Related Functionality

A large proportion of cereal‐derived fractions (Table 1) exhibited high antioxidant activity, as assessed by DPPH, ORAC, and FRAP assays, across diverse cereals and valorized streams, including bran and BSG (Bonifácio‐Lopes et al. 2022; Fall et al. 2025; He et al. 2020; Pan et al. 2025). These antioxidant activities primarily reflect the presence or release of phenolic compounds, pigments, peptides, and other redox‐active constituents and should therefore be distinguished from techno‐functional properties such as WHC, OHC, and emulsification. These antioxidant‐rich fractions align with growing consumer demand for functional foods that target oxidative stress and inflammation.

Some fractions also enhanced prebiotic effects, stimulating the production of short‐chain fatty acids (SCFAs) (e.g., butyric acid >0.90 g/L), improving probiotic growth, and increasing in vitro digestibility (Bhattacharya et al. 2020; Fareed et al. 2025; Sun et al. 2025). This was particularly evident for fiber‐rich extracts from wheat bran and BSG processed through enzymatic and M‐assisted techniques. Enhanced solubility and WHC (up to 8.05 g/g) supported use of such techniques in high‐moisture or gut‐health‐targeted formulations (Özen et al. 2024; Sukop et al. 2025). Such effects indicate that processing can generate biological functionality beyond the direct nutritional contribution of the recovered macronutrients. However, antioxidant capacity, enzyme inhibition, prebiotic activity, and related measurements should be interpreted as indicators of potential biological functionality rather than direct evidence of physiological effects in humans.

Processing can also alter biological functionality through the generation, release, or redistribution of bioactive peptides and phenolic compounds. As discussed above, <3 kDa peptide fractions obtained from hydrolyzed wheat germ and fermented cereal fractions exhibited comparatively strong radical‐scavenging activity (Pan et al. 2025; Tian et al. 2022). This demonstrates that protein fractionation and controlled hydrolysis can simultaneously influence nutritional accessibility and bioactivity. Nevertheless, the relationship is not necessarily linear because extensive hydrolysis may destroy peptide sequences responsible for specific biological effects.

These biological properties should be distinguished from techno‐functional attributes because antioxidant and prebiotic activities describe potential biological effects rather than the processing behavior of the ingredient itself. Nevertheless, they can complement functional characteristics in multifunctional formulations. For example, a protein–fiber fraction with high WHC and emulsification capacity can simultaneously provide structural functionality and deliver antioxidant or prebiotic components, thereby increasing its value in health‐oriented food systems. Similarly, the release of bound phenolics during processing can increase their accessibility to biological targets. M and cellulase treatment of barley released substantial proportions of bound phenolics during simulated gastrointestinal and colonic digestion, including ferulic acid and other phenolic compounds. These compounds may contribute to carbohydrate‐digestive enzyme inhibition through non‐covalent interactions and may complement the effects of soluble dietary fiber in controlling substrate accessibility. Such interactions illustrate how the biological activity of a cereal fraction may arise from the combined effects of several molecular components rather than from a single isolated compound. Prebiotic functionality provides another important biological dimension. Increased soluble fiber availability can enhance microbial F and SCFAs production, potentially supporting gut‐health‐related functionality. However, the magnitude and physiological relevance of these effects depend on fiber composition, F conditions, microbial community structure, and the extent to which the observed responses in in vitro systems translate to human gastrointestinal conditions.

### 4.4 Functionality and Application Potential

In cereal‐derived multifunctional fractions, structural modifications can simultaneously alter processing performance, nutrient accessibility, and biological activity. For example, enzymatic or US‐assisted disruption may improve protein solubilization and digestibility while generating low‐molecular‐weight peptides with antioxidant activity. M treatment may increase soluble dietary fiber and WHC while releasing bound phenolics and modifying starch accessibility. This interconnected behavior highlights the importance of evaluating cereal fractions according to their intended application rather than optimizing individual properties in isolation. Moving forward, integrating evaluations of multi‐FIs into system‐level formulation design is essential for guiding next‐generation food innovation. Pairing WHC and emulsification attributes with antioxidant performance could enable protein–fiber–polyphenol‐enriched emulsions or meat analogs (Gaber et al. 2025). Correspondingly, prebiotic‐active fibers coupled with slow‐release carbohydrates could offer potential for symbiotic or metabolic health‐targeted applications (Fareed et al. 2025). Fractions with high hydration capacity but low solubility may be directed toward solid or semi‐solid foods, whereas highly soluble and bioactive fractions may be more suitable for beverages or targeted delivery systems. Likewise, low‐molecular‐weight peptide fractions may be further incorporated into delivery systems when rapid bioaccessibility is desired, whereas fiber‐rich fractions with high WHC and swelling capacity may be better suited to foods requiring moisture retention and controlled digestion. Therefore, the selection of cereal fractions should consider a multidimensional performance profile encompassing technological functionality, nutritional quality, and biological activity. Such integrated evaluation can help determine whether a recovered fraction should be used primarily as a structural ingredient, nutritional protein source, bioactive component, or multi‐FIs combining several roles within the same food matrix. This approach also provides a stronger basis for connecting fractionation and processing strategies with formulation requirements and ultimately with the development of value‐added cereal‐based foods.

### 4.5 Biopolymer‐Based Functional Complex Design

The formation of novel food biopolymers is highly reliant on how extraction mechanisms influence structural characteristics and functional bioactivity. For instance, the functional qualities of cereal arabinoxylans are strongly influenced by their molecular mass, degree of branching, and ferulic acid concentration (He et al. 2021). Specific fractionation procedures have a direct influence on the nutritional and functional properties of grain fractions (Sukop et al. 2026). SWE is an effective approach for isolating cell wall polysaccharides with specific molecular structures from wheat, barley, and rye brans. The recovered extracts frequently shift from being glucan‐rich to being higher in feruloylated arabinoxylan as extraction time increases during SWE, significantly enhancing their radical‐scavenging activity (Ruthes et al. 2020). These structural changes determine the functional groups available for subsequent intermolecular association and influence whether recovered components remain dispersed, form soluble complexes, or undergo aggregation and phase separation. These interactions are relevant to the design of cereal‐derived systems for emulsification, encapsulation, controlled release, and structured food applications. Three interaction pathways are particularly relevant in cereal‐derived complexes: protein–protein, protein–polysaccharide, and protein–polyphenol interactions. Hydrogen bonding, hydrophobic interactions, electrostatic attraction or repulsion, and, in proteins, disulfide exchange contribute to these associations, although their relative importance changes with pH, ionic strength, temperature, and molecular concentration. Thus, molecular association is not inherently beneficial; the same interaction can improve network formation or bioactive retention at one condition but promote aggregation, precipitation, or reduced digestibility at another.

Protein–protein interactions determine whether extracted proteins remain soluble or form aggregates and gels. Protein unfolding during thermal, ultrasonic, or enzymatic processing can expose hydrophobic and sulfhydryl groups, increasing the potential for hydrophobic association and disulfide exchange. Conversely, electrostatic repulsion can maintain proteins in a dispersed state. Increasing ionic strength can screen this repulsion and favor hydrophobic collapse; for example, 0.1 M NaCl promoted aggregation of oat proteins and was associated with protein solubility below 7% (Sargautis and Kince 2023). This illustrates an important trade‐off: Structural opening can initially improve enzyme accessibility and solubilization, whereas subsequent intermolecular reassociation can reduce dispersibility and functional performance. Besides, protein–polysaccharide interactions can regulate protein association and water distribution through changes in the structural organization of the biopolymer network. Electrostatic attraction between oppositely charged protein and polysaccharide groups can promote complex formation, whereas hydrogen bonding and polymer entanglement can contribute to network stabilization. In cereal systems, disruption of native protein–polysaccharide associations by physical processing can increase soluble fiber and expose hydrophilic groups. For instance, M treatment of highland barley bran increased the fiber ratio and WHC after subsequent F (Fall et al. 2025). These interactions can improve hydration and viscosity and may restrict protein–protein association. However, excessive charge neutralization or unfavorable polymer ratios can instead result in coacervation, phase separation, or precipitation, emphasizing the need to control pH, ionic strength, and polymer composition. Moreover, protein–polyphenol interactions introduce an additional structural and functional trade‐off. Phenolic hydroxyl groups can form hydrogen bonds with peptide carbonyl or amide groups, whereas aromatic rings can interact with hydrophobic regions of proteins through hydrophobic association and π–π interactions. These interactions can stabilize or protect phenolics within a protein matrix, but strong association may also alter protein conformation and mask sites accessible to digestive enzymes. Polyphenol–protein complexes may reduce enzymatic accessibility during digestion while simultaneously protecting phenolics from premature release (Chen et al. 2023). Subsequent proteolysis or microbial transformation can weaken these associations and facilitate the release of smaller peptides and phenolic compounds.

The functional significance of biopolymer interactions arises from the balance between molecular association and structural accessibility. Strong protein–protein interactions may enhance gel strength and structural stability but compromise solubility, whereas protein–polyphenol interactions can improve bioactive retention while potentially restricting protein digestibility. Protein–polysaccharide association may enhance hydration and colloidal stability, but excessive association or unfavorable molecular ratios can promote phase separation and precipitation. As a consequence, fractionation and processing can shift the balance among hydrogen bonding, hydrophobic association, electrostatic attraction or repulsion, and covalent interactions (disulfide exchange), determining whether recovered components remain dispersed, form soluble complexes, or undergo aggregation. This structure–interaction–function relationship provides a mechanistic basis for tailoring cereal‐derived complexes for specific applications, including emulsification, encapsulation, controlled release, and digestion‐directed delivery. Therefore, molecular interactions should be considered dynamic determinants of cereal fraction functionality rather than isolated binding mechanisms, with processing conditions designed to achieve the required balance between association, accessibility, stability, bioactive retention, and digestion behavior.

#### 4.5.1 Nonthermal and Thermomechanical Restructuring

Nonthermal processing often induces controlled molecular modifications to enhance the functional potential of extracted ingredients. CP generates reactive oxygen and nitrogen species that depolymerize complex carbohydrates by rupturing glycosidic bonds in amylose, thereby affecting starch water solubility and simultaneously introducing new functional groups (e.g., carboxylic acids) (Bayati et al. 2024). Ozone‐assisted extraction operates via a comparable oxidative mechanism, serving as a pretreatment that oxidizes polypeptide backbones and cleaves peptide bonds, thereby facilitating the release of otherwise trapped flavonoids and soluble fibers (Jeevarathinam et al. 2026). US‐assisted techniques have been applied to extract protein–polysaccharide complexes and modulate their composition and interfacial elasticity by disrupting cell wall structures and promoting the release of previously inaccessible macromolecules (Zie et al. 2026). High‐power US drives acoustic cavitation, in which the rapid collapse of microbubbles generates intense localized shear forces; however, energy input must be controlled to prevent excessive free‐radical‐mediated lipid oxidation (Soni et al. 2021). Similarly, physical and thermomechanical approaches, such as high‐pressure homogenization (HPH) (Akyüz et al. 2024; Chen et al. 2023), thermal treatments, and PWE (Wu et al. 2020), enhance polymer recovery while improving functional properties and antioxidant capacity. Mechanical and thermal energies play a key role in restructuring biopolymers into novel food architectures. New inter‐ and intra‐molecular interactions, including disulfide bonds and hydrophobic associations, facilitate the formation of anisotropic, meat‐like fibrous structures by unfolding globular plant proteins under high heat and shear (Gulzar et al. 2025; McClements and Grossmann 2021). Superheated steam treatment induces distinctive structural modifications in cereals, promoting starch agglomeration by disrupting hydrogen bonds and inducing partial protein denaturation, which collectively alter dough rheology and flour microstructure (Liu et al. 2023). Combined thermal and nonthermal procedures have been applied to pearl millet flour to precisely modulate enzymatic activity, deactivating lipases responsible for rancidity while maintaining nutrient retention and microbial safety (Naraharasetti et al. 2025). These molecular changes can be monitored using non‐destructive spectroscopic techniques, such as attenuated total reflectance Fourier transform infrared spectroscopy and small‐angle x‐ray scattering, which monitor reductions in starch crystallinity and protein fragmentation following hydrothermal treatments (Sivakumar et al. 2023). It is essential to understand these structure–function relationships to develop plant‐based milk, meat, and egg analogs that replicate the thermodynamic behavior and solubility of their animal‐derived counterparts (McClements and Grossmann 2021).

US provides high extraction intensification at relatively low bulk temperature, with short treatment times and enhanced mass transfer, although scale‐up is constrained by non‐uniform cavitation and energy‐transfer efficiency. HPH is highly effective for cell disruption and particle‐size reduction and is particularly suitable for producing uniform colloidal structures, but its high‐pressure operation increases energy and equipment requirements (Chen et al. 2023; Zie et al. 2026). CP and ozone provide rapid, nonthermal oxidative modification and can improve matrix accessibility, but their oxidative nature requires precise control to avoid degradation of sensitive polymers and bioactives (Bayati et al. 2024; Jeevarathinam et al. 2026). Thermal treatments generally offer high throughput and reliable microbial or enzyme inactivation but require greater energy input and may cause protein denaturation, starch restructuring, or degradation of thermolabile compounds (Liu et al. 2023). The mechanistic relationships and potential complementarity of the technologies are shown in Figure 6. Each technology applies a specific physical or chemical perturbation to the molecular interaction landscape, thereby affecting component release, molecular conformation and aggregation state, and ultimately the functional properties of the resulting hydrocolloid. The technologies can be applied during the extraction phase to enhance component release and/or during the tailoring phase to reorganize the extracted compounds. Thus, US and HPH are primarily advantageous for physical disintegration and mass‐transfer enhancement, CP and ozone for rapid oxidative or functional modification, and thermal processing for high‐throughput restructuring and stabilization. Accordingly, process efficiency should be evaluated against the intended functional outcome rather than focusing just on extraction yield.

**FIGURE 6:**
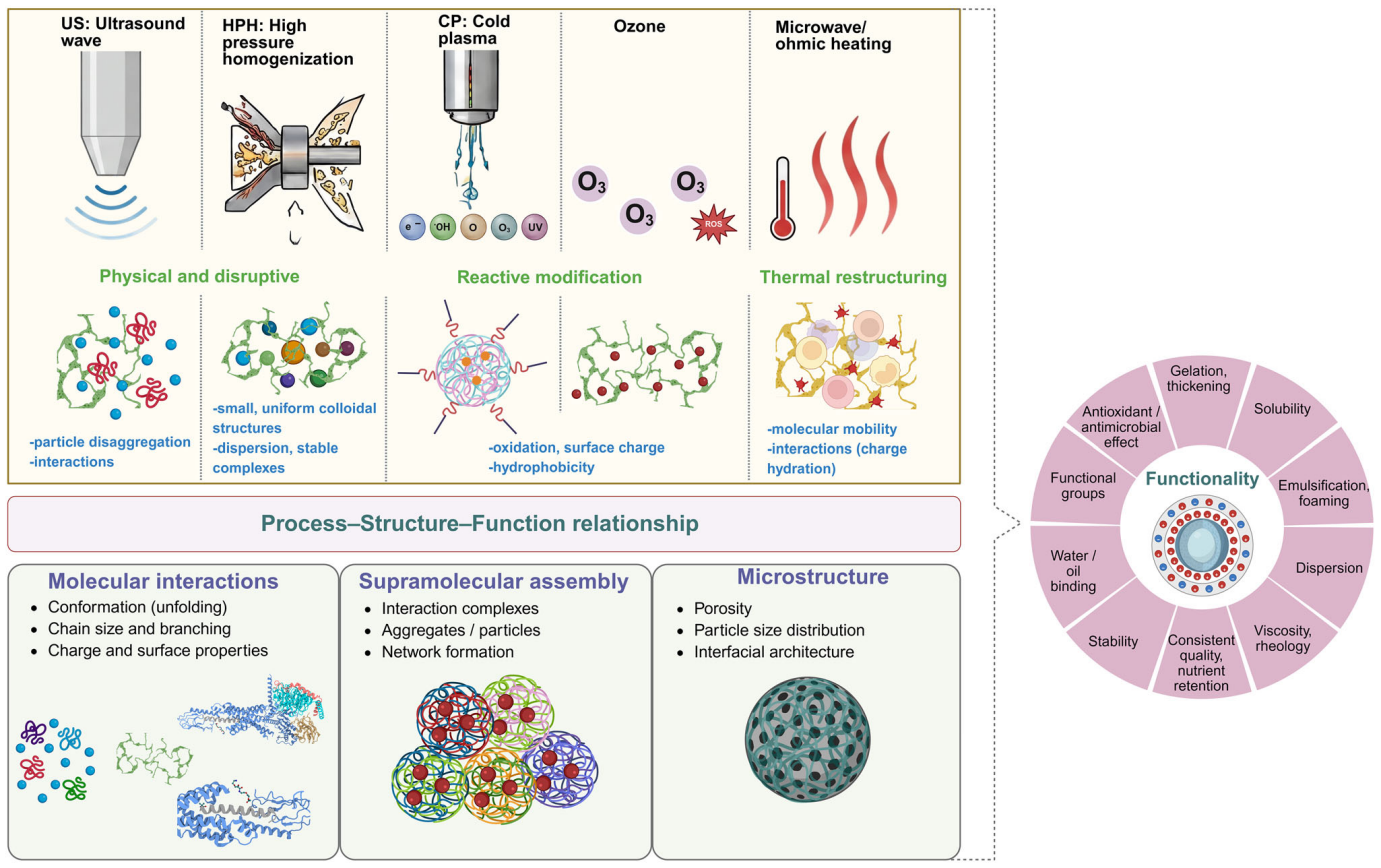
Effect of different processing technologies for structuring functional performance.

#### 4.5.2 Biochemical Functionalization

Highly effective methodologies, such as sequential acid–alkaline treatments followed by cellulase‐assisted hydrolysis, enable efficient extraction of protein–polysaccharide conjugates. This enzymatic intervention substantially enhances protein recovery compared with chemical treatments alone and produces biopolymers with improved emulsifying stability through controlled depolymerization of cell wall components (Bojorges et al. 2025). The approach also facilitates recovery of intracellular proteins and glucans for use as functional emulsifiers (Timira et al. 2024). In parallel, F provides a biological route to chemically restructure FIs and improve their bioaccessibility. Lactic acid bacteria F effectively reduces antinutritional factors, such as phytates and tannins, in plant‐based dairy analogs, alleviating protein digestibility inhibitors and converting off‐flavor aldehydes into more palatable compounds (Erem and Kilic‐Akyilmaz 2024). Biological processing further acts on cell walls to release insoluble‐bound phenolics; however, extensive dietary fiber can interact strongly with these compounds during digestion, potentially limiting their antioxidant activity (Rocchetti et al. 2022). In intact plant matrices, polyphenols are sequestered within vacuoles, but processing can cause interactions with cell wall polysaccharides, occasionally forming carbocations that covalently bind phenolics and alter their metabolic fate in the human gut (Liu et al. 2020).

Importantly, the effectiveness of biochemical functionalization is strongly dependent on enzyme–matrix specificity. Enzyme performance is governed not only by substrate composition but also by branching, substitution, cross‐linking, crystallinity, steric accessibility, and enzyme–substrate compatibility. α‐Amylase preferentially hydrolyzes α‐1,4‐linked starch structures, cellulases target β‐1,4‐glucan linkages in cellulose, whereas xylanases cleave the β‐1,4‐xylan backbone of arabinoxylans. For arabinoxylans, extensive arabinose substitution can hinder access to the xylan backbone (Bhattacharya et al. 2020; Xiao et al. 2024). Consequently, GH11 xylanases, which preferentially act on less‐substituted regions, are particularly suitable for relatively accessible wheat and rye arabinoxylans, and the broader substrate tolerance of GH10 xylanases can be advantageous for highly substituted arabinoxylans in bran‐rich matrices. In ferulate‐cross‐linked wheat bran, combining xylanases with feruloyl esterases can further improve substrate accessibility by disrupting ferulate‐mediated cross‐links (Ravichandran and Dixit 2025). Enzyme selection for protein functionalization should reflect protein architecture and the required degree of hydrolysis. Controlled proteolysis using enzymes (e.g., alcalase or trypsin) can partially disrupt compact storage‐protein structures, expose hydrophobic domains, and promote protein rearrangement and self‐assembly, whereas excessive hydrolysis may reduce the molecular integrity required for interfacial stabilization and nanoparticle formation (Yang et al. 2025). Thus, biochemical functionalization should be regarded as a matrix‐specific precision strategy in which enzyme specificity, accessibility, and degree of hydrolysis are optimized together rather than applied as a universal treatment. However, enzyme cost, thermal sensitivity, reaction time, and downstream enzyme removal remain practical constraints; enzyme immobilization, recycling, sequential dosing, and continuous enzymatic reactors may therefore improve process feasibility.

#### 4.5.3 Functional Co‐Extraction

Modern biopolymer research increasingly emphasizes the targeted co‐extraction of natural macromolecular mixtures rather than extensive and costly downstream processing. Sequential aqueous extraction enables the isolation of protein–polysaccharide‐enriched fractions in a mild and eco‐friendly manner, promoting synergistic enhancement of emulsifying and foaming properties while avoiding the use of harsh solvents (Li et al. 2023). These complexes can also be recovered directly from defatted biomass using alkaline extraction followed by isoelectric precipitation, with partial carbohydrate retention helping to preserve functional properties such as water‐holding capacity and structural stability (He et al. 2025). Importantly, the extraction and recovery conditions strongly influence the formation of highly viscous complexes with semi‐crystalline structures and enhanced thermal stability, supporting their potential applications in advanced food and material systems (Nuerxiati et al. 2022). However, the major challenge in functional co‐extraction is maintaining compositional and structural consistency. Co‐extracted protein–polysaccharide systems are inherently sensitive to raw‐material variability, including cultivar, maturity, season, geographical origin, and differences in protein‐to‐polysaccharide ratios. Small changes in ionic strength, pH, or mineral ion content can alter charge balance and shift the system between soluble complexes, coacervates, and insoluble precipitates, thereby changing recovery and interfacial functionality (Yang et al. 2025). Moreover, conditions that favor recovery of one component may compromise another; for example, aggressive solubilization of bound phenolics or cell‐wall polysaccharides may promote protein unfolding or degradation. Process heterogeneity can further amplify batch‐to‐batch variation. In US‐assisted extraction, differences in cavitation intensity, local heating, and shear exposure can modify protein conformation, surface hydrophobicity, molecular‐weight distribution, and interfacial elasticity. US treatment of protein–polysaccharide complexes increased β‐sheet content from 26.75% to 65.65%, surface hydrophobicity from 131.47 to 304.04, and interfacial dilatational elasticity from approximately 11 to 30 mN/m, while reducing solubility (Zie et al. 2026). These structural changes demonstrate that extraction intensity can improve long‐term interfacial stability while simultaneously compromising rapid solubility or emulsifying activity. Therefore, industrial co‐extraction requires tighter control of feedstock composition and process energy distribution, together with in‐line monitoring of composition and functional properties, to minimize batch‐to‐batch performance drift. Accordingly, feedstock standardization, predefined acceptable compositional ranges, process analytical technology (PAT), and adaptive control are needed to establish a robust operating window rather than relying on a single nominal extraction condition.

#### 4.5.4 Multiscale Engineering for Advanced Food Structuring

The production of advanced functional food ingredients can be enabled through targeted restructuring of agricultural by‐products. For example, BSG can undergo simultaneous extrusion and solid‐state F to substantially increase the release of soluble arabinoxylan, a dietary fiber that enhances bakery shelf life (Aradwad et al. 2025). Valorization of cereal bran and husk commonly employs subcritical water extraction to recover feruloylated arabinoxylans while deliberately preserving ester bonds essential for bioactivity (Hu and Gilbert 2025). At the colloidal scale, upcycled ingredients can be formulated into Pickering emulsions stabilized by protein‐based binary nanoparticles, in which hydrophobic and electrostatic interactions govern protein folding and interfacial adsorption. These emulsions serve as fat replacers and carriers of bioactive compounds, demonstrating the link between molecular aggregation and macro‐scale food functionality (Yang et al. 2025). Functionalization of by‐products also extends to advanced additive manufacturing, where structural porosity is engineered to optimize sensory and nutritional attributes. Three‐dimensional food printing incorporates food by‐products into extrusion‐ready inks, simultaneously fortifying the nutritional matrix and improving dimensional stability and layer resolution (Bareen et al. 2025). Printing parameters can be precisely tuned to generate hierarchical porous architectures, integrating macroscopic voids defined by the digital printing path with microscopic pores dictated by the viscoelastic and phase behavior of the bioink (Lombardi et al. 2025). This digital design strategy provides spatial control over texture, moisture dynamics, and nutrient release kinetics, demonstrating the integration of sustainable ingredient valorization into next‐generation, customized foods (Bareen et al. 2025; Lombardi et al. 2025).

Recent advances further extend this multiscale framework through AI and digital‐twin technologies. Machine‐learning approaches can assist in predicting molecular interactions, reducing the experimental search space for formulation design, whereas computational fluid dynamics‐ and finite element modeling‐based digital twins can connect ink rheology, nozzle flow, deposition behavior, and final structural fidelity across length scales (Lombardi et al. 2025; Yang et al. 2025). These tools are particularly relevant for upcycled ingredients because their heterogeneous composition creates strongly nonlinear relationships between formulation variables and printability. Nevertheless, digital twins should be based on validated mechanistic and experimental data because scale‐dependent phenomena, including heat transfer, mixing, acoustic attenuation, fouling, and residence‐time distribution, may limit direct transfer of laboratory‐scale models to industrial systems. Despite these advances, the industrial readiness of 3D food printing remains limited relative to conventional high‐throughput food manufacturing. Major constraints include insufficient standardization of printable formulations, limited printing speed, nozzle clogging, feedstock variability, hygienic design, post‐processing requirements, and regulatory and food‐safety validation (Bareen et al. 2025). Therefore, 3D food printing should currently be regarded as an emerging, application‐specific manufacturing platform rather than a mature high‐throughput alternative to conventional cereal processing. Its near‐term potential is strongest in applications where formulation flexibility and structural customization provide sufficient added value.

## 5 SFP in Cereal Fractionation

SFP should be reconsidered in the context of PB and CE; however, most cereal fractionation studies have focused primarily on yield and functionality. Integrating the circularity concept involves not only maximizing the recovery of proteins, fibers, and phytochemicals but also valorizing all co‐products (residue biomass) and minimizing waste streams (Fareed et al. 2025; Zhuang et al. 2024).

Nonthermal technologies, such as US, M, SFE, SWE, PWE, PEF, OH, CP, EH, F, and their combinations, have been introduced for a crop biorefinery (Bonifácio‐Lopes et al. 2022; Fall et al. 2025; Gaber et al. 2025; Li et al. 2021, 2025; Liu et al. 2021; Liu, Wu, et al. 2024; Martín‐García et al. 2020; Nemes et al. 2025; Özen et al. 2024; Pan et al. 2025; Sukop et al. 2025; Sun et al. 2025). These technologies have demonstrated the potential to enhance selectivity and purity of target ingredients, as well as nutrient accessibility and bioavailability. Importantly, preserving nutritional quality while minimizing processing intensity and associated environmental pressures is a key aspect of SFP (Perez‐Vega 2022). One approach to achieve both intentions is to adopt non‐conventional food processing technologies, as they often avoid the severe conditions of intensive thermal treatments that can degrade nutrients (Mir et al. 2016; Singh et al. 2016). These methods have been extensively reviewed in the literature for their potential to enhance energy and water efficiency while reducing waste generation (Chemat et al. 2017; Knorr et al. 2020; Teixeira 2018; Tsironi et al. 2021).

### 5.1 Sustainable Fractionation Strategies

The process of cereal fractionation can be improved by using various treatment and enhancement strategies to break down the complex plant structure and increase the accessibility of bioactive compounds, thereby improving the recovery of FIs. These methods can be used for multiple purposes, such as removing components like lignin and hemicellulose, reducing cellulose crystallinity, separating various bioactive elements, and increasing the surface area available for extraction (NEMES et al. 2024). Conventional procedures like thermal and steaming processes and mechanical or physical treatments, such as dry milling and flaking, can be applied before further processing to improve recovery and extraction (Aro et al. 2007; Bai et al. 2022). Additionally, such treatments can enhance the diffusivity and mass transfer mechanism into the plant matrix (Fernández‐Acosta et al. 2019). However, technological performance should be assessed beyond extraction yield. Dry fractionation offers low water demand and mature industrial operation but generally provides lower purity, whereas US, enzymatic, and other intensified processes can improve selectivity or recovery but may require greater process control, downstream separation, or CAPEX and OPEX requirements. Therefore, the most feasible technology depends on the target fraction, required purity, throughput, resource demand, and product value, as well as maximum extraction efficiency.

#### 5.1.1 Matrix Disintegration and Defatting

Another important treatment step is defatting, which can be performed with conventional solvents (e.g., hexane) or advanced SFE, which offers reduced solvent residues and improves environmental sustainability. Defatting represents a critical pretreatment step in protein–starch separation (Gaber et al. 2025; NEMES et al. 2024), and the removal of lipids improves the efficiency and selectivity of downstream processes. By eliminating lipid components, defatting enhances protein accessibility, reduces interference during aqueous extraction, and prevents lipid–protein complex formation that can hinder separation efficiency. Effective defatting also alters the physicochemical properties of the biomass matrix, increasing porosity and improving mass transfer. Furthermore, processes, such as SFE, PWE, and SWE, have been described as reducing solvent use, minimizing processing time, and being energy‐efficient and eco‐friendly, which may support the PB frameworks’ limits. SFE, in particular, is considered a green and safe method due to its non‐toxic and non‐flammable nature and can be easily separated from the extract. As it operates at a low critical temperature, it is suitable for extracting heat‐sensitive compounds (Argun 2022; Gaber et al. 2025; Özen et al. 2024; Rebolleda et al. 2020; Sahu et al. 2021; Yilmaz‐Turan et al. 2020). For instance, oily extracts rich in alkylresorcinols and tocopherol from wheat bran, obtained by SFE, have shown high antioxidant capacity and good storage stability (Rebolleda et al. 2020). According to Sahu et al. (2021), SFE defatting could yield higher phenolic levels than conventional methods. The process can be optimized by adjusting pressure and temperature; for example, for oil extraction from wheat bran, typical conditions were 30–35 MPa and 60–70°C (Argun 2022; Rebolleda et al. 2020). SFE has produced approximately 5.2%–9.6% higher oil recovery than *n*‐hexane, and defatting with SFE has subsequently increased phenolic recovery by approximately 0.85–2.6‐fold in selected wheat systems (Rebolleda et al. 2020; Sahu et al. 2021). The extracted lipid fraction can also retain valuable minor compounds, including alkylresorcinols, steryl ferulates, and tocopherols (Rebolleda et al. 2020). However, *n*‐hexane remains attractive because of its established technology, high extraction efficiency, relatively low equipment‐pressure requirements, and generally lower CAPEX and OPEX. The major disadvantages are toxicity, flammability, occupational and environmental concerns, and the need for solvent recovery and strict control of residual solvent in the defatted material. In contrast, SFE leaves essentially no conventional organic‐solvent residue and avoids solvent‐stripping operations but requires high‐pressure extraction vessels, CO_2_ compression, and associated capital investment. Therefore, although SFE offers clear advantages in solvent residue and process safety, higher equipment and compression costs can limit economic competitiveness unless the recovered lipid fraction has sufficient value or solvent‐free processing provides an additional product or market benefit. Moreover, SFE is primarily suited to lipophilic compounds and is less effective for highly polar phenolics without an appropriate food‐grade co‐solvent. Thus, selection between *n*‐hexane and SFE should not be based on merely lipid recovery but on the combined performance of recovery efficiency, environmental pressures, residual‐solvent requirements, downstream compatibility, costs, and the value of the recovered lipid fraction.

#### 5.1.2 Green Extraction

Despite these advantages, a critical trade‐off emerges between extraction dynamics and energy demand to maximize production and safety. High‐temperature treatments can achieve rapid processing rates and effectively degrade mycotoxins in cereals; however, they require immense continuous thermal energy inputs, which may create pressure on sustainability performance (Liu et al. 2023). Conversely, green solvent‐based extraction systems often operate under milder thermal conditions (<140°C), but achieving extraction kinetics comparable to conventional volatile solvents often requires process intensification through US‐ or M‐assisted processing, thereby increasing energy consumption (Mwaurah et al. 2020). This apparent contradiction illustrates that green extraction is not inherently low‐energy, but rather context‐dependent, necessitating thorough optimization of process intensity, yield, and environmental impact. Within this context, intensified techniques are nevertheless useful in this instance as they can enhance mass transfer, improve extraction efficiency, reduce solvent and energy demand, and modify functional properties such as foaming and water‐holding capacity compared to traditional methods (Anis et al. 2024; Ibrahim, Nadeem, Sultan, et al. 2024). However, their performance is also technology‐dependent; US‐ and M‐based methods are particularly effective for protein and fiber extraction, whereas SFE exhibits greater selectivity for lipid‐soluble phytochemicals and other antioxidant‐rich compounds (Burlini et al. 2020; Nemes et al. 2025). At the same time, excessive process intensity of the wave may limit the stability of heat‐ or oxidation‐sensitive phytochemical retention, particularly under high‐energy input conditions (Chen et al. 2024; Li et al. 2021; Liu et al. 2021). Furthermore, a significant change in the extraction strategy involves replacing traditional volatile organic solvents with more sustainable options. Aqueous systems based on nonionic surfactants and ionic liquids have shown their ability to solubilize hydrophobic compounds, such as carotenoids and chlorophylls, thereby enabling more selective recovery and easier downstream processing (de Souza Mesquita et al. 2021). SFE using carbon dioxide, often combined with food‐grade co‐solvents (e.g., ethanol), operates under specific phase pressure–temperature conditions that enhance solvent diffusivity and mass transfer, resulting in efficient extraction while limiting thermal degradation of sensitive molecules such as polyphenols (Ameer et al. 2017). In lipid recovery, aqueous enzymatic extraction breaks down cellular structures through targeted hydrolysis, facilitating the simultaneous release of oils and proteins without the need for hazardous organic solvents typically used in conventional Soxhlet extraction (Mwaurah et al. 2020). These considerations emphasize the necessity to conduct further investigations to simulate the process and assess the potential selectivity of the feasible technology for the desired nutrient‐rich fractions. Thus, the concept may involve designing fractionation strategies that are adaptable to specific biomass categories and target ingredients and effectively integrating them through complementary mechanisms and selectivity. Figure 7 illustrates intensified and hybrid processing arrangements achieved by combining physical, physicochemical, and biochemical methods.

**FIGURE 7:**
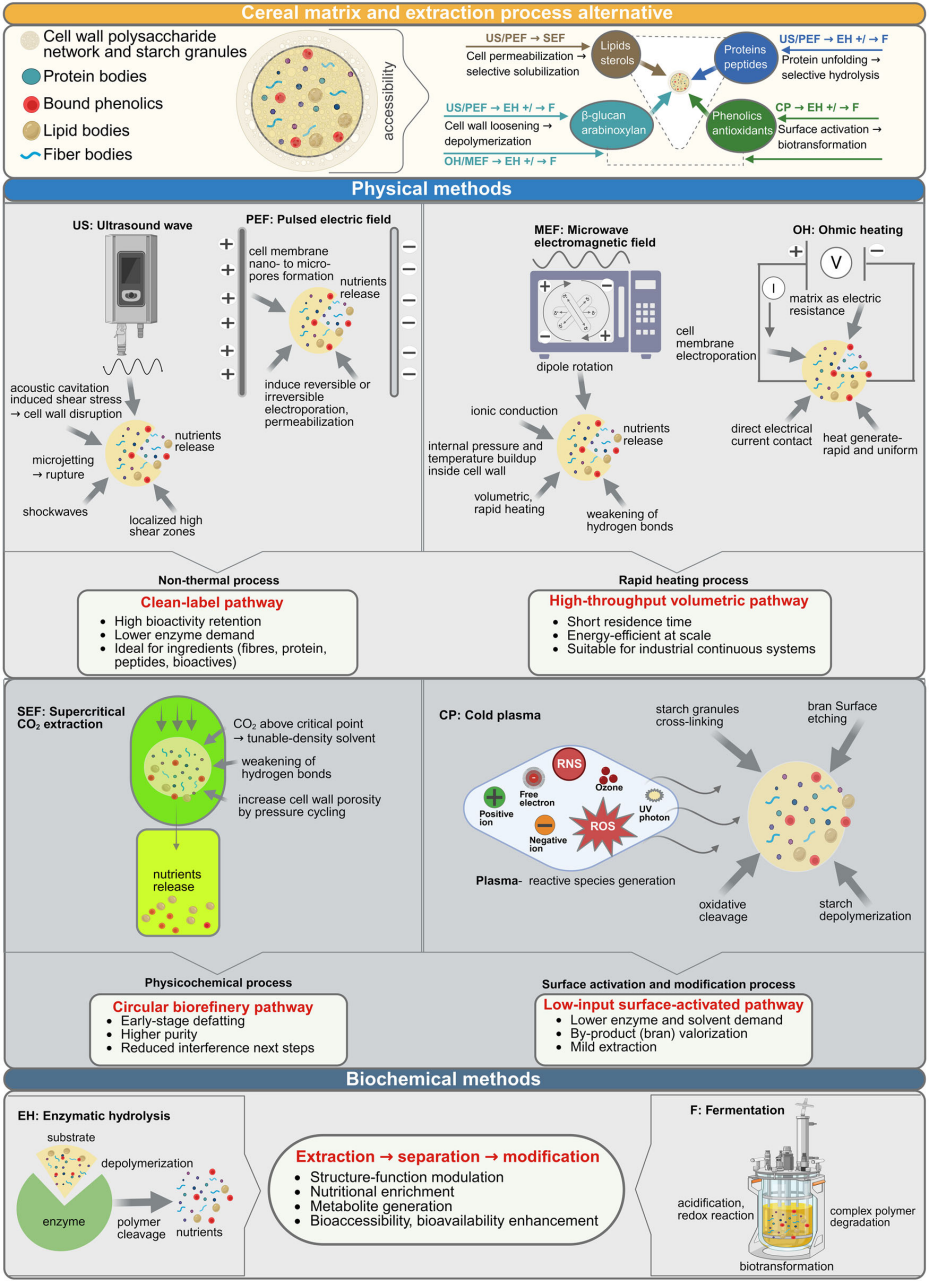
Innovative integrated conceptual processing arrangements for intensified and hybrid fractionation.

A further consideration for green extraction is solvent recyclability, because the environmental advantage of a solvent depends on its recovery and reuse over the complete process cycle. Natural DES offer low volatility and favorable safety characteristics, although their high viscosity and insignificant vapor pressure make conventional distillation inefficient. Therefore, antisolvent precipitation, membrane‐based separation, and low‐temperature evaporation can be considered for recovering the functional fraction and regenerating the DES for repeated extraction cycles (Ravichandran and Dixit 2025). Thus, solvent selection should be evaluated together with recovery efficiency, solvent lifetime, downstream separation requirements, and associated energy demand than on solvent composition. Likewise, energy optimization should target functional recovery rather than only extraction yield. Pulsed US can reduce continuous heat accumulation and cooling requirements while retaining cavitation‐driven mass transfer, whereas short, high‐intensity pretreatment followed by mild leaching can decouple cell disruption from prolonged energy‐intensive extraction. Multi‐response optimization using variables such as ultrasonic power, liquid‐to‐solid ratio, temperature, and residence time can identify operating windows that maximize FIs recovery while minimizing specific energy consumption (Zie et al. 2026). A higher extraction yield does not necessarily indicate genuine process intensification when it is achieved at disproportionate energy or solvent demand.

#### 5.1.3 Multimodal Process Integration

Researchers have been investigating cereal side‐stream biomass to extract high‐value components for improving functional food ingredients, and the aim is to apply existing green technologies in various combinations during processing (Li et al. 2025). To create more sustainable and efficient processes, these hybrid approaches aim to achieve maximum yields with shorter processing times and minimal resource use (Cengiz et al. 2021; Guerrini et al. 2020). Some treatments, like physical, enzymatic, and chemical, can give a clear concept to advance the food processes to overcome the limitations of using a single method; the most crucial part is how to merge the potential methods to develop a process innovation with respect to targeted products (Li et al. 2025). The principal multimodal processing pathways and their potential sequence of unit operations are illustrated in Figure 8. As the goal is to fractionate, for example, to modify the structure of polysaccharides such as barley β‐glucan, here US technology can play a significant role in reducing aggregation and potentially enhancing its antioxidant activity (Cao et al. 2023). Another promising method, M and US‐assisted, has been identified for recovering β‐glucan from hull‐less barley bran (Liu et al. 2021). This technique introduced pretreatment with ethanol, followed by combined treatment with enzymes to remove starch and protein (Li et al. 2021). Where the M‐assisted technique has been demonstrated to directly extract polysaccharides during the heating process, and the robust hybrid process, M–US, has been revealed to be an excellent potential approach for extracting β‐glucan from hull‐less barley bran (He et al. 2020; Li et al. 2021; Liu et al. 2021). Furthermore, food processes can also be improved by integrating with biological methods such as US‐based EH, and if necessary, the enrichment and improvement of products (e.g., nutritional value and functional properties) through F (using microorganisms like *Lactobacillus plantarum*, *Aspergillus niger*, *B. subtilis*) may be considered the most effective way to enhance the bioactive content of antioxidants, phenolics, protein digestibility, mineral and vitamin bioavailability, and the soluble dietary fiber (Hassan et al. 2025; NEMES et al. 2024; Nemes et al. 2025; Pan et al. 2025). Likewise, CP and high hydrostatic pressure nonthermal technologies also enhanced the yield and functional properties of soluble dietary fiber from wheat bran during EH (Li et al. 2025; Martín‐Diana et al. 2021). Processes may be advanced when the green techniques are merged in a diverse manner, and a US‐assisted three‐phase partitioning strategy revealed an emerging strategy when oil‐, protein‐, and fiber‐rich fractions had been separated in a single step. As nonthermal treatment could simultaneously separate different oat fractions, while also enhancing yield, reducing extraction time, and lowering solvent consumption, it might offer valuable insights into process synergy and improvement for further investigation (Perez‐Vega et al. 2023). During fractionation, US increases contact surface and analyte diffusion, enhancing solvent penetration into the plant matrix. Identifying efficient extraction parameters is crucial for determining the most significant factors to optimize using response surface methodology (RSM) (Cengiz et al. 2021). Solvents were being explored for extracting oat proteins and polyphenols, sometimes in combination with ultrasonication, to facilitate processes and avoid harsh hydrolysis treatments (Cherif et al. 2020; Perez‐Vega et al. 2023). One of the novel thermal treatments is SWE, which uses water at high temperatures (100–374°C) and pressure to separate hemicelluloses from plant biomass, and it is considered a green process for producing biomaterials from hemicellulose‐rich biomass (e.g., wheat bran) (Li et al. 2020). Moreover, another efficient approach is to break down the complex and heterogeneous polysaccharide network in starch‐rich biomass (e.g., cereals, which have a higher content of dietary fiber and phenolic compounds), and this modification into prebiotic dietary fiber step could be performed by applying a suitable mixture of enzymes, such as cellulase, xylanase, and ferulic acid esterase, which had been employed to release targeted nutraceuticals (e.g., ferulic acid and arabinoxylan‐oligosaccharides) (Bautista‐Expósito et al. 2020; Martín‐Diana et al. 2021). Basically, EH is a targeted approach for releasing specific FIs to avoid the use of toxic chemicals often found in other purification processes, and it depends on selecting the right combination of enzyme classes; for instance, amylases and proteases could be used to remove interfering starch and protein, followed by xylanases and feruloyl esterases to release the ferulic acid (Ferri et al. 2020). Correspondingly, α‐amylase and trypsin can be used to remove starch and protein, respectively, to isolate β‐glucans. Destarching can be performed with α‐amylase, which is crucial, followed by specific enzymatic treatments to release target molecules (Bhattacharya et al. 2020; Li et al. 2021; Liu et al. 2021). This technique is the most attractive green alternative due to its energy efficiency and high selectivity, and it operates under mild conditions that preserve the organoleptic properties (Bautista‐Expósito et al. 2020; Li et al. 2025).

**FIGURE 8:**
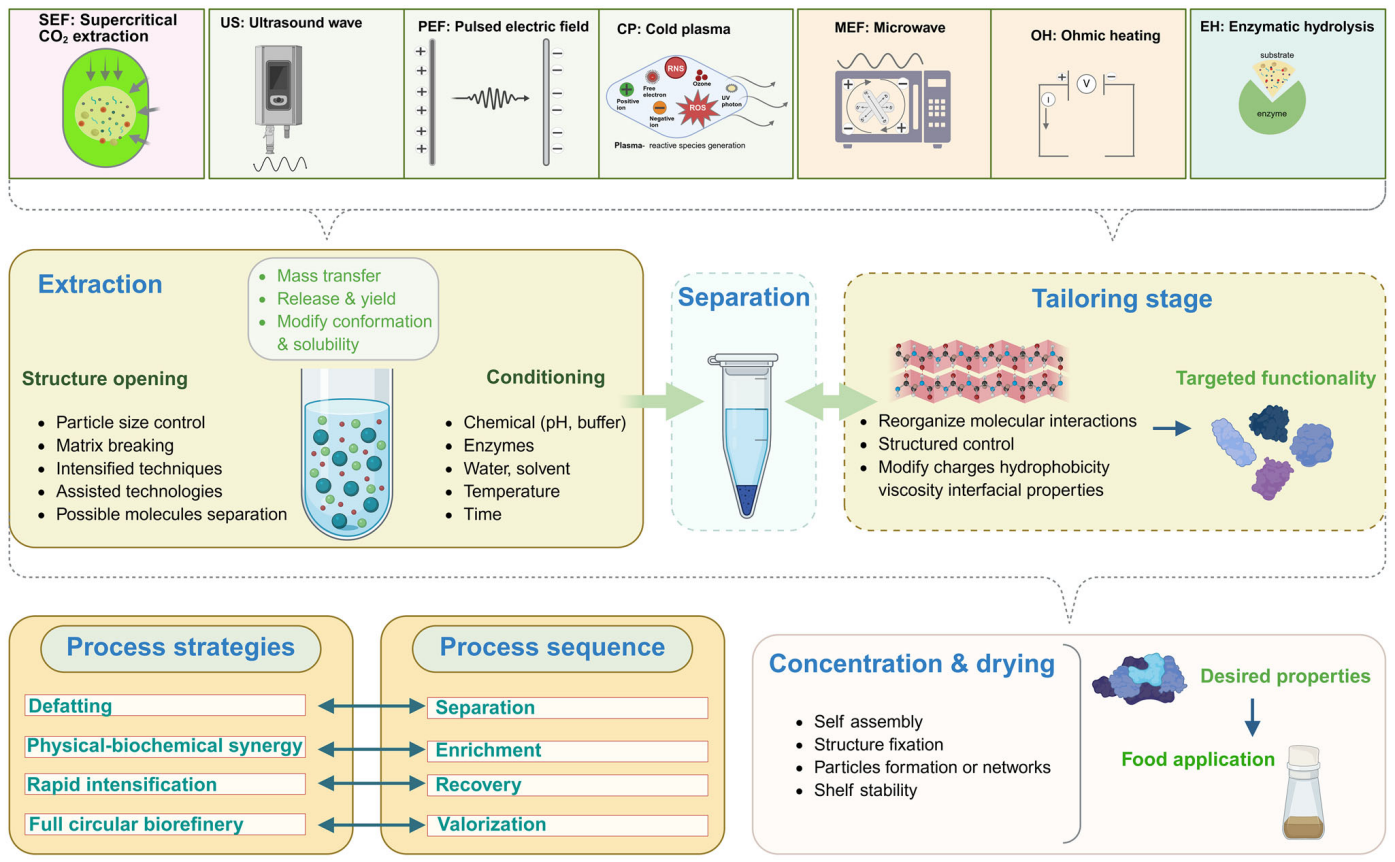
Integrating processing sequences flowchart for fractionation.

Despite these demonstrated synergies, compatibility between unit operations becomes a critical constraint during scale‐up. As illustrated in Figure 8, the compatibility of sequential unit operations becomes a critical constraint during scale‐up. High‐intensity US and EH should generally be operated sequentially because the localized thermal and mechanical effects generated by cavitation can reduce enzyme activity. Enzymatically treated slurries may retain heterogeneous fiber particles that can restrict HPH through nozzle blockage or excessive equipment wear. Hence, upstream particle‐size control and solid–liquid management are important before high‐pressure processing (Yang et al. 2025). Integration with additive manufacturing introduces an additional rheological constraint. HPH can generate finer and more uniform colloidal structures, but excessive homogenization may alter WHC and reduce the yield stress or storage modulus required for stable filament deposition. An intermediate conditioning step, such as controlled concentration or mild cross‐linking, may be necessary to recover printability before extrusion (Bareen et al. 2025; Lombardi et al. 2025). These interactions indicate that multimodal processing cannot be optimized by maximizing the performance of individual operations independently; instead, residence time, temperature, particle size, enzyme activity, rheology, and downstream process requirements must be co‐optimized across the complete processing line. Accordingly, industrial scale‐up would benefit from PAT, including in‐line spectroscopic, acoustic, and rheological measurements, to detect changes in feedstock composition and dynamically adjust operating conditions. This adaptive control is particularly important for agricultural side streams, where upstream variability can propagate through enzymatic conversion, particle formation, and final product structuring.

### 5.2 CE and Systemic Biorefinery Concept

Although single‐process approaches excel at producing high‐purity isolates, particularly protein from oat and wheat, they often generate underutilized biomass streams (e.g., bran or BSG); the consequence may contradict CE principles (Olalere and Gan 2023; Sargautis and Kince 2023). Consequently, other valuable components, including dietary fiber, phytochemicals, and prebiotics, are frequently underexploited or discarded. This scenario diminishes all aspects of recovery potential and keeps the processing model in a segregated, narrow state. By contrast, integrated biorefinery approaches, combining multiple techniques, have shown potential for multi‐nutrient extraction while enhancing prebiotic activity (Fareed et al. 2025; Rudjito et al. 2024), butyrate production (Zhuang et al. 2024), and digestibility and glycemic control (Bai et al. 2022; Xiao et al. 2024). In addition, the cereal side streams like BSG and bran are rich in prebiotic fibers and antioxidants but are often used only as low‐value animal feed or discarded (Alonso‐Riaño et al. 2020; Martín‐García et al. 2020). Where combined fractionation for BSG yielded prebiotic oligosaccharides and enhanced SCFAs production, demonstrating true circular valorization potential (Fareed et al. 2025; Zago et al. 2022). Furthermore, merging techniques of M, US, and EH have extracted enhanced fiber and facilitated protein or phytochemical yields from barley, which illustrates multi‐component valorization (Li et al. 2021; Liu et al. 2021).

#### 5.2.1 Upcycling‐Based Resource Recovery

Value‐added recovery necessitates a shift from linear fractionation to upcycling‐oriented system design, in which underutilized side streams are reconceptualized as functional resource streams rather than waste. Upcycling agricultural and industrial residues expands the scope of circular biorefineries by converting low‐value side streams into high‐value FIs. Circular biorefineries can utilize diverse biomass sources to produce antioxidant and nutritionally important compounds through fractionation strategies aligned with green chemistry principles (Yin et al. 2026). The efficiency of phytochemical recovery from crop residues is strongly influenced by process parameters such as solvent polarity, extraction pressure, and system selectivity, which determine both yield and functional quality (Sanatombi 2025). In addition to physicochemical processing, emerging biotechnological routes enable the repurposing of processing waste through microbial metabolic engineering, facilitating the production of dietary supplements and novel bioactive metabolites without the need for entirely new processing infrastructures (Liu, Gao, et al. 2024). Collectively, these strategies reinforce the importance of system‐level design in which biomass is utilized holistically, maximizing value extraction while minimizing environmental pressures. Such integration helps advance CE principles in cereal biorefineries by recovering more from each fraction.

#### 5.2.2 Lifecycle and PB

According to those prior observations, the recent technology performed effectively with enhanced recovery and is still associated with green processing principles, although the system‐level sustainability impacts of those processes have been inadequate in most reviewed studies. Hence, in addition to extraction conditions, sustainability should be emphasized to be evaluated using comparable indicators, including energy consumption, water use, solvent consumption and recovery, GHG emissions, eutrophication, and ecotoxicity. Nonetheless, Perez‐Vega et al. (2023) evaluated detailed cradle‐to‐gate LCA metrics of the US‐assisted two‐phase precipitation process and identified critical environmental hotspots linked to butanol manufacture, which dominated freshwater eutrophication (89.11%), global warming potential (GWP) (87.19%), water consumption (89.26%), and terrestrial ecotoxicity (87.16%). Similarly, land‐use impacts (98.93%) were primarily attributed to oat production, underscoring the upstream agricultural trajectory (ISO 2006). Moreover, the solvent recycling sensitivity analysis demonstrated that increasing recovery rates from 0% to 90% can noticeably reduce impacts across most factors and create a clear circularity opportunity. For example, freshwater eutrophication potential declined by >60%, GWP dropped from around 45 kg CO_2_‐eq/kg protein isolated to <10 kg CO_2_‐eq, and water consumption was reduced by nearly 80%. In addition, terrestrial ecotoxicity was an important concern, with impact contributions exceeding 80% in scenarios using higher butanol concentrations (50% volume). As a result, it is primarily linked to solvent production and upstream agricultural inputs. However, land‐use footprints remain essentially unchanged and reflect the fixed demand for agricultural areas for feedstock production. Even though PB frameworks have not yet defined a precise global threshold for chemical pollution or ecotoxicity (Steffen et al. 2015; Willett et al. 2019), LCA showed considerable insight regarding the novel entities (e.g., synthetic chemicals and heavy metals). These results demonstrate that the environmental performance of fractionation depends not only on the extraction technology but also on upstream biomass production, solvent selection, solvent recovery, and process integration. Thus, technology‐level sustainability claims should be based on the complete process system; this emphasizes the urgency of integrating sustainable process design criteria.

From the PB perspective, reductions in eutrophication, GHG emissions, water consumption, and ecotoxicity directly help keep the biorefinery's footprint within the boundaries of biogeochemical flows, climate change, freshwater use, and novel entities. Nevertheless, persistent land use poses a risk of overshoot not only at the land‐system change boundary but also in relation to biosphere integrity, as agricultural land expansion and intensification are directly associated with biodiversity loss and ecosystem degradation. These impacts are not currently mitigated by downstream processing innovations and therefore require upstream interventions targeting both land‐use efficiency and biodiversity preservation. Even at high solvent recovery rates, significant residual terrestrial ecotoxicity persists, indicating that mitigation strategies must extend beyond end‐of‐pipe solutions. To address these challenges, upstream interventions, including low‐impact feedstock sourcing, greener solvent alternatives, and the deployment of closed‐loop chemical recovery systems, are required. Such measures are important to ensure that cereal fractionation operates within safe and just PB limits while simultaneously advancing multiple SDGs. Thus, an innovative biorefinery concept could be considered to reveal the SFP context, as shown in Figure 9, which always introduces a zero‐waste model and a safe processing space to enable multi‐stream valorization.

**FIGURE 9:**
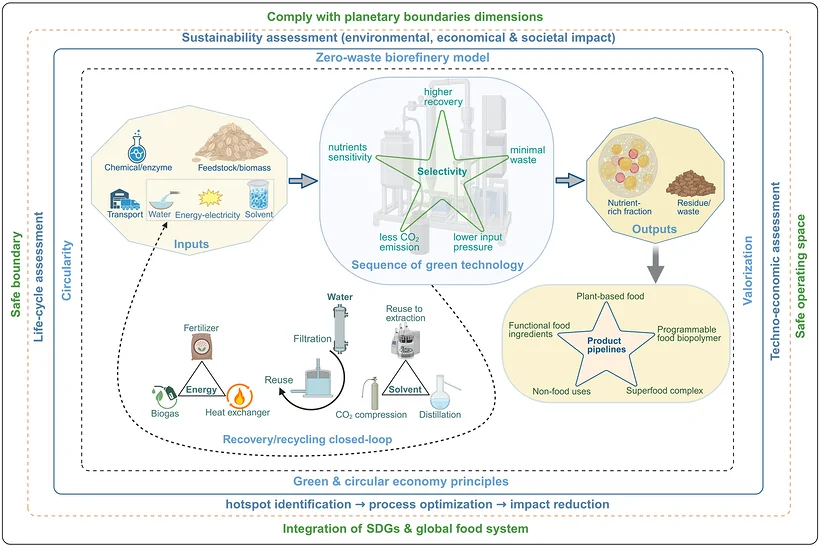
SFP model by novel biorefinery and circular bioeconomy concept.

#### 5.2.3 Techno‐Economic Feasibility

The scalability and industrial uptake of green fractionation technologies were constrained by the absence of TEA. Most studies operated at a lab scale (≤400 g batches), with very few scaled up or modeled, whereas CAPEX, OPEX, and energy trade‐offs in a PB context were not yet examined for proof‐of‐concept and industrial adoption. Although pilot‐scale SFE and EH have been demonstrated for grain and bran (Gaber et al. 2025; Radenkovs et al. 2021; Sukop et al. 2025), their economic competitiveness versus conventional methods remains unknown. In line with this, energy‐intensive methods such as SFE and US can be environmentally favorable when optimized for solvent use; nonetheless, without process integration, they may be prohibitively expensive (Özen et al. 2024; Perez‐Vega et al. 2023). For industrial translation, CAPEX and OPEX should also be considered in conjunction with throughput, product value, downstream processing, equipment availability, and co‐product revenue. Accordingly, TEA should be performed using a consistent functional unit and system boundary before claims of economic feasibility are made. At minimum, future assessments should emphasize CAPEX, OPEX, energy consumption, water consumption, solvent consumption and recovery, labor, maintenance, waste treatment, product yield, product purity, co‐product credits, and revenue per unit of biomass processed. Sensitivity analysis should additionally examine raw‐material price, energy price, solvent recovery, equipment utilization, product selling price, and plant capacity. Recent studies demonstrate the value of combining process simulation with TEA and LCA because process intensification does not necessarily produce economic or environmental benefits when additional downstream operations are required (Wang et al. 2024). Although ultrasonic stress germination treatment represents a bioprocessing rather than a conventional fractionation strategy, the 60‐h processing period highlights an important scale‐up trade‐off between enhanced bioactive enrichment and process throughput (Liu, Wu, et al. 2024). Integrated biorefineries must be optimized not solely for protein, fiber, or phytochemical yields, but also for the joint recovery of whole biomass within a unified recovery model, and for distributing costs across multiple revenue streams, thereby improving competitiveness (Fareed et al. 2025; Zhuang et al. 2024). Moreover, integration of energy and water recycling loops may enhance both economic durability and PB compliance, though these interventions require upfront CAPEX for closed‐loop systems. The design and scale‐up of the state‐of‐the‐art biorefinery model for food processing can be considered within a boundary scenario that encompasses the nutritional, environmental, economic, and social dimensions. Therefore, future research should incorporate variable optimization to reduce the impact. Correspondingly, for dynamic process innovation, modeling and simulation approaches using LCA and TEA can enhance scale‐up feasibility and industrial deployment.

#### 5.2.4 Regulatory Frameworks and Safety Implications of Green Fractionation

Regulatory constraints, solvent and chemical residuals, and food‐grade safety requirements must be carefully addressed. Because these factors are critical for market translation as well as the transition from laboratory to industrial‐scale adoption, ensuring consumer safety requires alignment with EU circular bioeconomy frameworks and relevant global food safety standards. Novel solvents (e.g., DES, *t*‐butanol) and enzymes, while demonstrating efficiency gains, raise questions about residuals, food‐grade safety, consumer acceptance, and regulatory consent; therefore, they require precise consideration of regulatory scope or food contact safety and intended food use (Olalere and Gan 2023; Perez‐Vega et al. 2023).

##### 5.2.4.1 Regulatory Classification Systems

The regulatory landscape for phytochemicals intended for use as food additives is governed by complex classification systems and stringent safety assessments that can act as significant bottlenecks to innovation. The concept of “novelty” under Regulation (EU) 2015/2283 is defined as a “novel food,” meaning any food not used for human consumption to a significant degree within the EU before May 15, 1997, which triggers a comprehensive authorization process that may take an average of 2.56 years from submission to approval (Le Bloch et al. 2025; Kaim et al. 2025). Even when derived from historically consumed sources, some traditionally consumed preparations (e.g., aqueous plant extracts) may be considered non‐novel in specific regions; more purified fractions or those obtained through non‐conventional extraction methods are frequently reclassified as novel foods (Kaim et al. 2025). Beyond novelty, regulatory classification is also influenced by the intended technological function of the phytochemical. When used as food additives (e.g., antioxidants, colorants, or stabilizers), these compounds must meet strict purity, exposure, and toxicological requirements under frameworks such as EU Food Additive Regulation (EC) No. 1333/2008, which defines food additives as any substances not typically consumed as food itself but added deliberately to food for a technological purpose. As a result, highly purified extracts designed for consistent performance often face a dual regulatory burden, in which both additive approval and novel food authorization may be required, depending on their composition and level of modification. Accordingly, regulatory classification should be established early in process development because the intended food application, degree of purification, structural modification, and production method can influence the type and extent of safety evidence required.

##### 5.2.4.2 Safety and Risk Assessment

Safety evaluation remains one of the most challenging aspects of phytochemical commercialization. Regulatory authorities such as EFSA often require extensive toxicological datasets, often pausing the evaluation process to request additional information, thereby delaying market access (Le Bloch et al. 2025). This complexity is compounded by the heterogeneity of plant‐based extracts, whose safety profiles depend on biomass origin, processing conditions, and the presence of naturally occurring or process‐induced contaminants (Intrasook et al. 2024). These include risk assessment strategies: the application of the threshold of toxicological concern for low‐exposure compounds; the use of documented history of safe consumption for higher exposures; and detailed chemical characterization combined with in silico toxicity modeling when data gaps remain (Roe et al. 2018). However, such frameworks are not always sufficient for highly modified or purified phytochemicals, particularly when their structural and functional properties deviate significantly from traditional food matrices.

In addition, food‐safety assessments should address contaminants and consider the raw material history that may originate from upcycled cereal side‐streams or become concentrated during fractionation, including mycotoxins, microbiological hazards, pesticide residues, heavy metals, environmental contaminants, and process‐induced contaminants. Fractionation does not inherently reduce these hazards and may preferentially partition or concentrate undesirable compounds into protein‐, fiber‐, bran‐, or phenolic‐rich fractions. Therefore, contaminant partitioning should be evaluated together with compositional enrichment during process development. Solvent residues represent further safety consideration for extraction routes. Solvent selection should consider food‐grade status, permitted use, residual limits, recovery efficiency, worker exposure, and waste treatment. Closed‐loop solvent recovery together with validated residual‐solvent analysis is required before food application. Allergen management is also relevant to cereal fractionation, particularly in integrated facilities processing wheat, barley, rye, and gluten‐free cereal streams. Cross‐contact may occur through shared equipment, processing lines, or insufficient cleaning, making segregation, cleaning validation, traceability, and appropriate labeling important components of food‐safety design. Similarly, microbial safety becomes increasingly important when aqueous extraction, EH, germination, or F is introduced. High‐moisture conditions and extended processing times can increase the opportunity for microbial proliferation and require hygienic process design, microbial monitoring, and appropriate control of F and storage conditions.

##### 5.2.4.3 Implications in Fractionation

A critical but often underexplored issue is the role of fractionation in driving regulatory outcomes. The transition of phytochemicals from their native food matrix into concentrated functional additives involves a “molecular transformation” that directly influences their classification and safety assessment. During intensive fractionation, the removal of structural associations with cell wall polysaccharides alters the stability, bioavailability, and metabolic behavior of these compounds (Liu et al. 2020). This structural de‐compartmentalization can shift phytochemicals from being considered part of a traditional food matrix to isolated bioactive substances with potential dose‐dependent toxicity. For instance, polyphenols that exhibit safe antioxidant behavior when bound within plant fibers may act as pro‐oxidants at high concentrations when purified, thereby requiring more rigorous toxicological evaluation (Liu et al. 2020; Rocchetti et al. 2022). In addition, harsh extraction conditions (e.g., high temperature, extreme pH, or solvent‐driven depolymerization) may generate new chemical entities, such as oxidation products or covalent derivatives, which are treated as novel entities by regulatory authorities (Liu et al. 2020). The same concentration effect can apply to undesirable constituents. Milling and fractionation may redistribute heavy metals and other contaminants between bran, germ, protein, and fiber streams, particularly when these compounds are associated with mineral‐rich or outer grain tissues. Therefore, safety evaluation should be performed at the level of the recovered fraction rather than inferred only from the safety profile of the original cereal biomass.

##### 5.2.4.4 Technological Uncertainty

The choice of extraction technology is therefore a key determinant of regulatory feasibility. Although advanced techniques such as SFE, US‐assisted extraction, and innovative solvents improve yield and sustainability, they can also alter phytochemical composition and introduce process‐specific uncertainties that complicate regulatory approval (Akdemir Evrendilek 2026; Hndeya et al. 2026). Even when the source material has a history of safe consumption, significant compositional changes induced by processing may trigger reclassification as a novel food or require additional safety validation. Moreover, regulatory acceptance depends not only on composition but also on demonstrating the absence of process‐induced contaminants and ensuring stability and reproducibility at scale (Ahamed et al. 2025; Akdemir Evrendilek 2026). These criteria create a regulatory‐technical gap in which advancements in green extraction technologies exceed existing frameworks’ capacity to adequately assess their safety consequences. Process validation is essential for demonstrating that laboratory‐scale safety and quality performance can be reproduced at industrial scale. Dry fractionation approaches such as air classification and tribo‐electrostatic separation can reduce solvent and water requirements and may better preserve native protein functionality; however, their lower fraction purity may result in the co‐concentration of antinutritional factors, requiring appropriate compositional and safety verification. Critical process parameters may include temperature, pressure, pH, moisture, residence time, enzyme activity, solvent concentration, and microbial load, whereas critical quality attributes may include residual solvents, contaminant concentrations, composition, purity, moisture stability, and functional performance. Validation should also demonstrate reproducibility across raw‐material lots and operating cycles because cereal biomass can vary substantially in cultivar, storage history, and side‐stream origin.

##### 5.2.4.5 Integration Into Process Design

A fundamental concern exists between the industrial drive for high purity and the regulatory preference for compositional familiarity and historical use. Although purification enhances functional performance and standardization, it simultaneously removes the native matrix context that supports established safety evidence. Thus, increasing purity often leads to increased regulatory attention. This creates a regulatory dilemma: the same processing steps required to achieve consistent additive functionality may also increase the likelihood of triggering more severe safety assessments due to altered bioavailability, higher exposure levels, and loss of historical consumption data. Consequently, a successful regulatory pathway requires regulatory‐by‐design or matrix‐aware fractionation strategies. The processing approaches should be designed to balance functional performance, structural integrity, and regulatory compatibility. Mild extraction techniques, such as enzymatic treatments or controlled SFE, can help preserve the association between phytochemicals and dietary fibers, thereby maintaining more predictable safety profiles and facilitating alignment with existing regulatory categories (Rocchetti et al. 2022; Yin et al. 2026). In this context, the development of standardized functional complexes, rather than ultra‐purified isolates, represents a promising strategy. These systems retain key structural features of the original food matrix while delivering targeted techno‐functional properties, allowing the use of existing safety data to support regulatory approval pathways. Process design should also incorporate hazard analysis and critical control point principles, particularly for integrated cereal biorefineries involving aqueous processing, F, multiple cereal feedstocks, and shared processing equipment. This approach can link critical control points with contaminant monitoring, allergen control, microbial safety, and process reproducibility rather than treating food safety as a downstream regulatory requirement.

By integrating regulatory considerations directly into process design and optimization, the translational potential of phytochemical fractionation can be significantly enhanced. In this context, developing a functional food readiness framework can facilitate early alignment between extraction strategies and regulatory requirements, thereby reducing downstream barriers to approval (Akdemir Evrendilek 2026). Such a framework integrates matrix‐preserving extraction (Liu et al. 2020), predictive bioaccessibility modeling (Rocchetti et al. 2022; Yin et al. 2026), and safety‐by‐design processing (e.g., use of food‐grade solvents and compliance with maximum residual limits) (de Souza Mesquita et al. 2021; Yin et al. 2026) within international safety standards and regulations to support more efficient approval processes (Kaim et al. 2025). In parallel, incorporating real‐time monitoring technologies and AI‐driven process modeling provides a promising basis for meeting regulatory expectations for consistency, traceability, and safety in complex formulations (Hndeya et al. 2026). However, technological validation does not merely ensure market acceptance. Consumer perception of ingredient naturalness, familiarity, labeling, sensory quality, color, flavor, texture, and perceived health benefits can influence adoption of fractionated ingredients. Clean‐label and solvent‐free processing may improve consumer acceptance, whereas thermal browning, oxidation, undesirable flavor development, or poor formulation performance may create market barriers. Hence, sensory evaluation, oxidative stability, shelf‐life assessment, and consumer acceptance should be considered alongside safety and process validation during scale‐up. Industrial adoption depends not only on technical performance but also on regulatory status, ingredient familiarity, sensory quality, labeling requirements, and consumer perception of the processing technology and resulting ingredients.

#### 5.2.5 Integrated Biorefinery

The sequential recovery of different valuable compounds from a single stream can be made attainable by deploying integrated biorefinery models for maximizing resource utilization. To achieve efficient fractionation in lignocellulosic biomasses typically requires the coordinated application of physical, chemical, and biological treatments to break the complex structural bonds and improve the accessibility of target compounds (Ale et al. 2025). This is particularly important for cereal bran, where functional constituents, such as carbohydrates, lipids, proteins, phenolic acids, and bioactive peptides, are often tightly bound to cell wall matrices, thereby limiting their extractability and bioaccessibility (Bamigbade et al. 2025). Consequently, the combination of specific treatments with bioprocesses (e.g., F, EH) is essential to enhance their release and functional performance (Nemes et al. 2022). Solid‐state F of wheat bran using fungal strains, such as *Trichoderma*, has been shown to increase phenolic and amino acid content, resulting in enhanced antioxidant properties that persist even after subsequent enzymatic extraction (Dey et al. 2026). More broadly, such multi‐step valorization strategies can convert cereals and side‐streams into a diverse range of products, including dietary fibers, FIs, and edible biomaterials, supporting a comprehensive approach to resource efficiency and reducing GHG emissions (Ravichandran and Dixit 2025). Although the integration concept of these technologies shows immense promise, the large‐scale implementation of these biorefineries faces constraints. Process scalability remains challenging due to variability in biomass composition and processing batches, which complicates process standardization and optimization (Adil et al. 2026). In addition, economic feasibility and process integration remain key barriers, particularly when combining multiple unit operations with differing energy and resource requirements. To address these hurdles, technological advances and supportive policy frameworks will be essential for facilitating industrial adoption (Ravichandran and Dixit 2025).

#### 5.2.6 Zero‐Waste Biorefinery

The development of zero‐waste biorefineries requires a shift from conventional single‐component purification to the co‐extraction of natural functional complexes. The different purified isolates are often at the expense of yield, and substantial residual biomass and chemical waste streams are generated. In contrast, co‐extraction strategies retain naturally associated macromolecular systems (e.g., protein–polysaccharide, polyphenol–protein–polysaccharide complexes), thereby enabling more thorough biomass utilization and sustaining functional synergies. This transition is typically achieved through the integration of green treatment and sequential extraction strategies. Mechanical and physicochemical approaches, for instance, improve the accessibility of intracellular components and facilitate subsequent fractionation steps. When combined with mild aqueous extraction or enzymatic treatments, they provide the progressive recovery of multiple fractions from a single feedstock, thereby minimizing reliance on organic solvents and extreme pH conditions (Bojorges et al. 2025; Li et al. 2023). This integrated performance demonstrated significant recovery of complex conjugate fractions from defatted biomass, alongside conserving functional properties and reducing waste generation (Li et al. 2023). Likewise, physical disruption techniques coupled with EH enhance the recovery of intracellular proteins, glucans, and phenolics for use as FIs by releasing bound components from recalcitrant matrices (Bojorges et al. 2025; Zie et al. 2026). These cascading extraction strategies ensure that both supernatant and residual fractions are valorized, resulting in a more systematic conversion of biomass into value‐added products (He et al. 2025). However, there are significant challenges to industrial deployment. Energy demand associated with downstream operations, particularly drying and fraction stabilization (e.g., lyophilization or centrifugation), can offset sustainability gains. In addition, the use of less‐refined fractions introduces variability in composition and likely retains anti‐nutritional factors or non‐desired compounds that are carried through into the final product. This places greater emphasis on raw materials control and processing conditions. Process scalability and continuous operation design require the development of low‐energy separation and dewatering technologies, improved process integration, and robust optimization strategies. In this context, membrane‐based separations, continuous‐flow extraction platforms, and hybrid processing configurations may offer viable pathways to translate zero‐waste biorefinery concepts from laboratory to industrial scale.

### 5.3 Process‐Functional Implications in Food Biopolymer Systems

Laboratory‐scale studies often exhibit high extraction efficiencies that do not translate directly to industrial operations. Techniques such as microencapsulation and precision F can effectively retain the bioactivity of polyphenols; however, maintaining these controlled conditions in continuous large‐scale systems introduces regulatory complexity and sensory variability (Yin et al. 2026), thereby remaining a key engineering limitation (Bermudez‐Aguirre and Niemira 2023). In industrially relevant processing, nonthermal technologies introduce important biochemical performance trade‐offs. CP application delivers strong activity through reactive oxygen species; nevertheless, these same species can promote non‐selective oxidation of polyunsaturated fatty acids and vitamins, which can affect nutritional quality (Bayati et al. 2024). Conversely, PEF and ultraviolet irradiation can effectively preserve sensitive bioactive molecules in liquid and solid food systems (Hirneisen et al. 2010; Soni et al. 2021). The optimal hydrothermal treatment can inhibit endogenous lipase activity, increasing shelf life and reducing rancidity without the need for chemical preservatives (Naraharasetti et al. 2025). Beyond preservation, biological processing plays a key role in the functional product's restructuring. Lactic acid bacteria F in plant‐based systems reduces antinutritional factors such as phytates, hydrolyzes allergenic proteins, and produces organic acids that enhance the masking of undesirable notes in flavor (Erem and Kilic‐Akyilmaz 2024). In cereal‐based bakery systems, similar microbial interventions improve dough rheology and contribute to natural preservation through the formation of bioactive metabolites (Dong and Karboune 2021). At the structural level, F and ingredient engineering are increasingly integrated into design strategies such as 3D food printing, in which agro‐industrial residues are used to generate hierarchical porosity that reduces density while maintaining mechanical integrity in structured foods (Bareen et al. 2025; Lombardi et al. 2025). These structure–function phenomena are critical for altering the complex architecture of animal‐derived foods using plant‐based ingredients (McClements and Grossmann 2021). However, upcycled ingredient systems continue to face barriers related to consumer acceptance, high processing costs, particularly for fractionation of BSG, and internal structural variations in biomass structures that influence polyphenol–polysaccharide interactions (Aradwad et al. 2025; Liu et al. 2020). The valorization of bran and agricultural residues is constrained by heterogeneous composition and limited predictability of bioactive release, which reduces scalability and functional consistency (Hu and Gilbert 2025; Sanatombi 2025).

#### 5.3.1 Challenges for Scale‐Up and Valorization

In the development of circular food biorefineries, transitioning green extraction strategies from laboratory‐scale protocols to industrial‐scale production may pose several challenges, including potential modifications to inputs, different dilution ratios, and the need to re‐evaluate costs for larger operations (Liu et al. 2021; Perez‐Vega et al. 2023). The principal scale‐up challenges include continuous feeding and discharge, heat and mass transfer, acoustic or electromagnetic field uniformity, solvent recovery, enzyme stability, fouling, membrane flux, wastewater treatment, product drying, equipment availability, and quality‐control requirements. However, prior few investigations often simulate continuous, high‐throughput industrial systems using simplified batch systems in the lab (Li et al. 2020). Ferri et al. (2020) developed a process for treating bran by scaling up a bioreactor 40‐fold, and the targeted molecule release was successfully replicated at the smaller scale, though it required adjustments. Likewise, high‐moisture extrusion processes were scaled up using the same core technique, with modifications such as a cooling die and adjustments to feed rate and screw speed to accommodate various material properties (Gaber et al. 2025). As the described green extraction methods demonstrated promising fractionation of plant‐based food materials, several investigations were conducted to scale up the advanced food biorefinery concept, as summarized in Table 5, thereby constructing an industrially feasible process that minimizes steps and resource use (Ferri et al. 2020). Nevertheless, process scaling may introduce hurdles in achieving process efficiency, ensuring product quality, and managing resource use (Climent Barba et al. 2022). Several factors can be considered during processing, including inconsistent raw material varieties or compositions, unpredictable effects of energy use, and limitations in controlling or monitoring temperature and time at larger volumes, which may result in undesirable yields and functionality (Milićević et al. 2021; Prandi et al. 2022). In this instance, phenolic extraction from cereal brans is influenced by complex kinetics; in particular, this effect is related to particle size and solvent properties (Milićević et al. 2021), whereas higher solids loading in EH may lead to unnecessary mixing and increased pumping power demands (Climent Barba et al. 2022). Moreover, various processing technologies were explored for their efficiency and scalability in valorizing food by‐products; thermal methods were investigated for effects that could provide insights for industrial production (Hong et al. 2022). One emerging technology, SFE, is recognized as a promising, environmentally friendly method for the efficient extraction of valuable, heat‐sensitive compounds (Sahu et al. 2021). Nowadays, the spray‐drying method is the most widely used in industry to stabilize downstream products into powders, and it is a straightforward, rapid, and relatively inexpensive technology. However, this may risk damaging sensitive components at high temperatures (Martín‐Diana et al. 2021). These potentials were identified as the optimal time and temperature for an energy‐efficient, scalable pathway. Most studies have determined optimal conditions for different cereal feedstock varieties using RSM for yield; however, although these advanced technologies are energy‐efficient, further investigation is needed into their process innovation and system‐level design. Even though an effective lab scale may face difficulties in maintaining optimal process conditions and gradients over larger volumes, thus, all of these challenges emphasize a critical requirement—this is not only a focus on the preliminary level of experimental protocol for lab‐scale study but also offers a scale‐up of robust innovation in real‐time insights into process dynamics, moving beyond traditional, time‐consuming offline analyses as a result of advanced process control strategies (Liu et al. 2022).

**TABLE 5:** Scale‐up biorefinery for cereal‐to‐ingredients processing using advanced techniques.

Feedstocks	Technology	Protein (% w/w)	Fiber/Prebiotic (% w/w)	Phytochemical (% w/w)	Process conditions	Functional evaluation	References
Maize	PEF	43	26.7	—	Lactic acid, pH 2.5; PEF energy input: 50 kJ/kg; PEF field strength: 1.5 kV/cm ; steeping time: 40 h	Enhanched water‐holding capacity of fiber fraction by 44%; better protein hydrophobicity	Sukop et al. (2025)
Wheat	US + EH + F	13.99	66.2	0.063	Power: 80 W, 40 kHz; pretreatment times: 30 min	Higher antioxidant activity	Pan et al. (2025)
Sorghum	EH	64.25	—	—	Steeping: 36 h; enzymatic hydrolysis: 2 h amylase, 1 h cellulase	Better water‐holding capacity (2.78–6.06 g/g); oil‐holding capacity (3.47–6.69 g/g); 84.85%–86.38% in vitro digestibility	Xiao et al. (2024)
Rye bran	EH	—	—	0.37	Enzymatic hydrolysis time: 12–72 h	—	Radenkovs et al. (2021)
Wheat bran	EH	—	—	0.26		—	
Oat bran	EH	—	—	0.03		—	
Rye bran	SFE			0.46	SFE parameters varied: temperature (40–60°C), pressure (10–25 MPa); modifier (7.5%–20% ethanol)	—	
Wheat bran	EH	10.36	15.21	1.18	Hydrothermal treatment: 15 min; hydrolysis with lactic acid; spray‐drying at 130°C	Higher antioxidant activity	Martín‐Diana et al. (2021)
Wheat bran	SFE	—	—	2.5	Pressure 25 MPa; temperature 40°C	Higher antioxidant activity	Rebolleda et al. (2020)
Wheat bran	EH	15.8		1.2	Pretreatment with alcalase and termamyl; final hydrolysis with pentopan and feruloyl esterase	—	Ferri et al. (2020)
Wheat bran	SWE+EH	—	74.2	1.6	Temperature: 160°C, time: 5–30 min; hydrolysis time up to 48 h	Not quantified directly; describe the potential of nutraceutical, prebiotic, biomedical applications	Rudjito et al. (2024)

*Note*: Yield values were collected from the original studies and standardized to a uniform unit (% w/w). Although some methodological heterogeneity exists, including differences in cereal type, pretreatment, extraction conditions, and product purity, only yield data were aggregated by processing method and biomass type. Data on process conditions and functional evaluations are presented in detail, as reported in the original studies.

Abbreviations: EH, enzymatic hydrolysis; F, fermentation; PEF, pulsed electric field; SFE, supercritical fluid/CO_2_ extraction; SWE, subcritical water extraction; US, ultrasound.

#### 5.3.2 Digitalized Decision‐Support Strategy for Scale‐Up Engineering

A rapid, low‐cost, and accurate method is in high demand, particularly in food industry renovations, to analyze product quality and address variability in biomass materials, processing efficiency, and resource‐use suitability (Lin et al. 2024). The concept is to employ predictive modeling and digital twin frameworks for scalable food production processes by translating insights from advanced fractionation technologies, as depicted in Figure 10. This strategy is used in diverse industrial systems; however, researchers and stakeholders may consider applying it to the cereal fractionation industry. The analytics, vibrational spectroscopy like Fourier‐transform infrared (FTIR) or attenuated total reflectance FTIR (ATR‐FTIR), which includes near‐infrared (NIR) and mid‐infrared (MIR) regions, can offer a powerful solution for overcoming these scale‐up hurdles by enabling rapid, non‐destructive characterization of biomass and its transformed fractions (Levasseur‐Garcia 2018; Liu et al. 2022; Topi et al. 2025). This technique is cost‐effective and can identify molecular‐level fingerprints of compounds’ chemical structures, either qualitatively or quantitatively (Shan et al. 2025). These spectral fingerprints may be used to build robust prediction models when coupled with chemometric tools and machine learning algorithms. For instance, several kinds of tools and algorithms might be employed depending on the application, such as principal component analysis (PCA), partial least‐squares regression (PLS‐R), unsupervised domain adaptation (UDA), random forest, and artificial neural networks (ANNs) (Lin et al. 2024; Shan et al. 2025). These models can correlate spectral data with important quality attributes and process parameters regarding the extraction yields for the desired ingredients (e.g., β‐glucans, arabinoxylans, proteins, and phenolics) with critical sustainability factors and reaction kinetics (e.g., EH progress), coefficients, solute release, or structural changes in the biomass (Barron 2011; Kuchta et al. 2022). The spectral states of the prospect are predicted by trained models, as Kuchta et al. (2022) showed using encoder‐decoder neural networks for enzymatic reactions. Interestingly, a significant advancement over reactive adjustments is the ability to anticipate process deviations and implement proactive control. Hence, this potential may enable operators to control the inherent variability of grain feedstocks, which frequently causes irreproducibility in industrial settings. For online FTIR detection, consistent measurement conditions, such as temperature, humidity, and instrument setup, are required (Shan et al. 2025).

**FIGURE 10:**
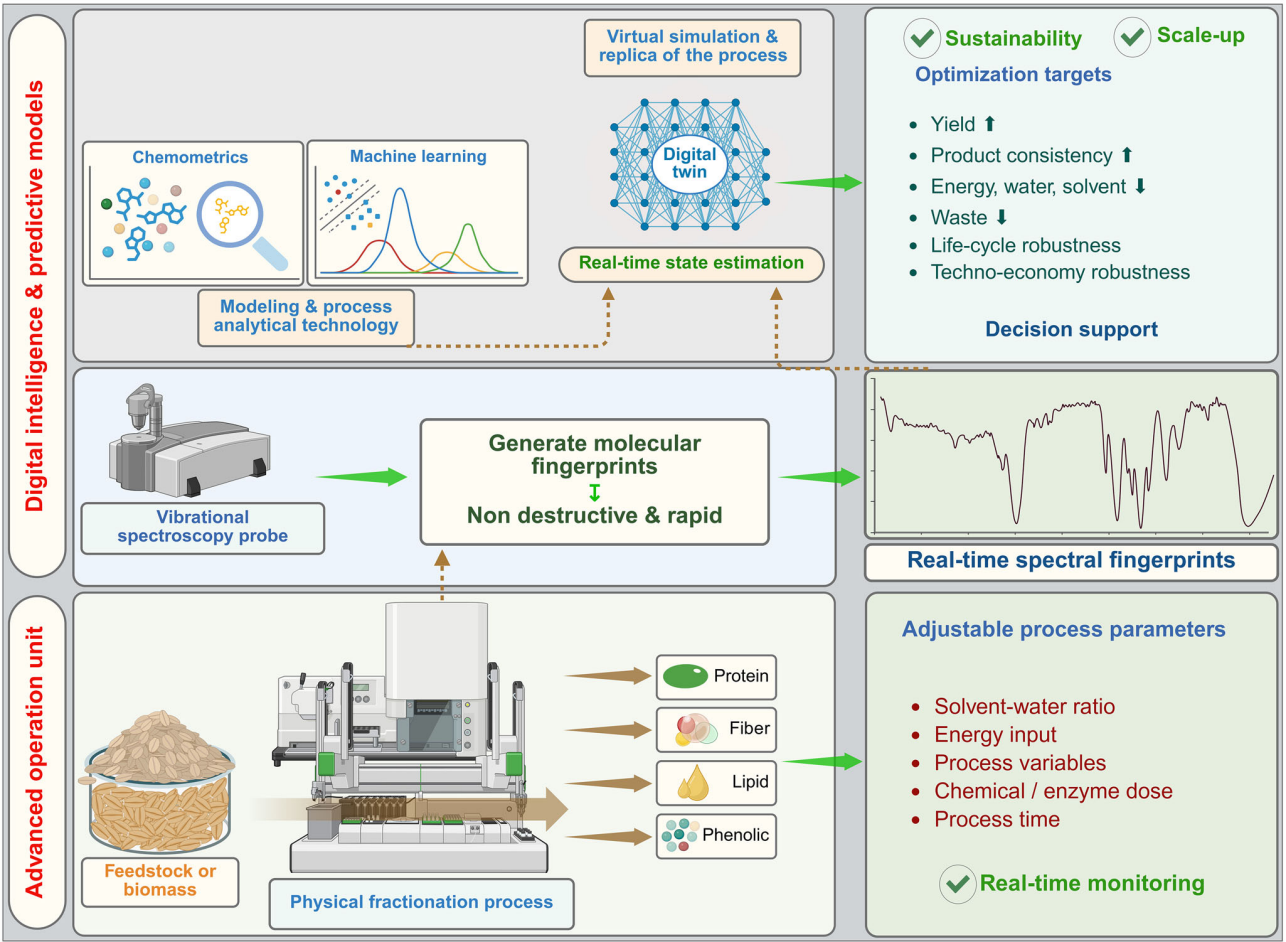
Digital twin framework for scalable and adaptive biorefinery processes.

Moreover, spectroscopy can be used as a PAT tool, and this implementation can be a critical conceptual advancement, facilitating continuous, real‐time monitoring of process performance. In particular, inline ATR‐FTIR probes can monitor chemical or molecular transformations and solute concentrations within a reactor or process channel without interfering with operation (Veale et al. 2007). This real‐time observed data channel can allow adaptive control loops to autonomously regulate process considerations such as solvent/water‐to‐solid ratios, operation times, power or energy levels, and enzyme or chemical uses; as a result, this digital twin system can maintain consistent product quality while producing high yield and minimizing the consumption of resources (Galanakis 2022). The execution of the PAT‐based systems can transform cereal biorefineries into self‐calibrating, resource‐efficient, and resilient processing units. This might become a strategic digital approach to shift from static, predefined operational setpoints to dynamic, data‐driven optimization (Tufail et al. 2022). A highly precise digital twin of the food biorefinery system could be achieved through a real transformative development. Correspondingly, spectroscopy‐informed predictive models could be integrated into process simulation software (e.g., Aspen Plus or SuperPro Designer) (Aranda‐Barradas et al. 2025; Çilak et al. 2023). Generally, using a digital twin tool to improve or operate a food industrial system can provide continuous updates with real‐time PAT data, enabling simulation of the full process under variable conditions and resulting in a dynamic virtual representation of the physical operation (Bartling et al. 2021). Importantly, the most expensive or impractical circumstances require a suitable solution, often for experimental investigation at scale (Eisenmenger et al. 2006). This virtual setting can facilitate the in silico evaluation of process‐escalation approaches, the optimization of energy trade‐offs, and solvent‐recovery loops. Furthermore, digital twins can improve the precision of LCA and TEA by replacing static assumptions with dynamic, empirically embedded data; thus, this may mitigate uncertainty in the design and scaling up of cereal biorefineries that are both feasible and ecologically sound (Bartling et al. 2021). The circular biorefineries always desire a boundary‐safe process operation in order to support SDGs and PB frameworks, and in these cases, optimization of input consumptions with respect to output benefits (high yield, zero waste, and minimal environmental impact) is necessary; to address the ambiguous situation, the fusion of multi‐sensor data along with the development of standardized and transferable models will be critical for accelerating the transition from proof‐of‐concept extractions to industrial scale (Arzami et al. 2022; Taylor et al. 2024). SFP is especially relevant as it integrates both global food system perspectives and the operational and economic realities of industrial business environments. In this regard, the decision‐support conceptual framework can be leveraged as a feasible innovation pathway to build a powerful digital tool that supports the design of a techno‐economic basis for de‐risking scale‐up.

## 6 Process Synergies and Valorization Opportunities in Cereal Biorefineries

Among the reviewed studies, oat and barley emerged as the most dominant cereals, reflecting both heightened research interest and their commercial significance. Although the multi‐step processes dilute protein concentration due to co‐extraction of additional components, they align more closely with biomass valorization principles, facilitating more circular use of cereal matrices. Moreover, underexplored climate‐resilient cereals, such as sorghum, rye, and millet, and pseudocereals (e.g., buckwheat and quinoa), as well as industrial by‐products (e.g., BSG and bran), offer potential opportunities for valorization as input biomass due to their complex, nutrition‐rich structures. They provide high protein content and a high‐quality prebiotic potential but lack robust data on the integrated recovery of fiber or phytochemicals. In addition, BSG and bran are promising by‐products with CE potential that require targeted fractionation to enhance protein isolation and optimize co‐product valorization, given their high availability and rich nutrient composition. Hence, the reviewed studies indicated the importance of simultaneously recovering multiple ingredient modules (e.g., protein, fiber, lipid, phytochemicals, or potential nutrient‐rich complex fractions) from the same process stream. Process synergy is crucial for determining a design by optimizing variable conditions for whole‐biomass valorization, as CE principles can be perceived as a sustainable, edible biorefinery impression. Despite the broad functional scope, relatively few studies (Table 1) had explicitly assessed the behavior of cereal‐derived fractions within multi‐ingredient or complex food matrices. Functional evaluations remain largely ingredient‐centric, with limited translation into prototype formulations or product development pipelines. There is also an imbalance in the literature, with most studies focusing on single‐function evaluations (e.g., only antioxidant capacity or WHC), rather than integrated assessments of synergistic performance (e.g., texture, digestibility, bioactivity‐bioavailability, and bioaccessibility). Cereal side streams such as BSG and bran exhibited promising multifunctionality but require optimized fractionation to decouple co‐extracted compounds that may hinder functionality (e.g., high lignin content, antinutrients). The lack of scale‐up data or pathways and the absence of CAPEX/OPEX evaluations raise uncertainty about industrial translation. Although promising integrated biorefineries were proposed but not fully spectrum‐valorized, the absence of validated scale‐up models impedes adoption in industrial contexts. At present, commercial‐scale evidence for the intensified technologies reviewed here remains limited, although conventional dry fractionation, milling, air classification, and extrusion have greater technological maturity. Pilot‐scale demonstrations provide encouraging evidence for SFE‐integrated oat protein fractionation, including downstream air classification and extrusion (Gaber et al. 2025), and OH of wheat bran, which reported approximately 63% lower energy consumption than the conventional process (Al‐Hilphy et al. 2020). However, these demonstrations indicate technical feasibility rather than confirmed commercial deployment. For M, US, and enzymatic fractionation, reported improvements in extraction or recovery remain predominantly at laboratory or pilot scale, whereas equipment requirements, process control, residence time, and operating costs require further validation at commercial throughput. Thus, the current evidence supports promising industrial transition but does not yet establish commercial maturity for most intensified cereal fractionation technologies. In this context, alongside these observations, digitalized decision‐support strategies could accelerate process optimization, enhance reproducibility, and facilitate industrial deployment. Accordingly, emphasis should be placed on balancing critical methods with LCA and technological readiness levels, such as those that assess not only process yield, technical feasibility, and product functionality but also systemic sustainability.

A critical consideration emerges at the material level, namely, the stability–functionality trade‐off associated with co‐extracted and less‐refined fractions. Co‐extraction strategies are often promoted to preserve native protein–polysaccharide–polyphenol interactions and enhance apparent antioxidant capacity. The literature also emphasizes evaluating the shelf‐life stability of complex matrices relative to their purified counterparts. Oxidative degradation may occur if not adequately controlled due to the presence of residue from chemicals or enzymes, lipid fractions, or retained moisture. These structural interactions can also restrict bioaccessibility, mass transfer, absorption kinetics, and subsequent biotransformation because polyphenols are often partially bound to cell wall protein–polysaccharide networks. As a result, improvements observed in vitro functionality may not directly translate into in vivo efficacy. From a processing perspective, energy intensity and operational complexity may give rise to environmental pressure. Nevertheless, sequential co‐extraction and integrated fractionation strategies support zero‐waste objectives and unit operations such as separation, drying, concentration, and stabilization. Moreover, the assumption that nonthermal or intensified techniques are inherently benign requires critical reassessment. Processes involving high shear, acoustic cavitation, or reactive oxidative species—associated with US, CP, or similar technologies—may induce unintended structural modifications, including protein cross‐linking and lipid oxidation, thereby affecting both nutritional quality and functional performance. These critical aspects may pose challenges in linking biopolymer structure to functionality and end‐use application. Cereal‐derived biopolymer structuring is well established under controlled laboratory conditions. However, applications demand opportunities to generate further knowledge of their behavior within complex food matrices or under dynamic physiological conditions.

## 7 Future Perspectives

Industrial implementation poses challenges due to the requirement to balance fraction purity and environmental sustainability in both dry and wet fractionation procedures, as well as limited pilot‐scale validation and insufficient integration of energy and solvent recovery. Furthermore, scalability may hinder the transition because high‐selectivity technologies such as SFE require significant capital investment and complex operations outside laboratory settings. Moreover, intensive processing steps can lead to undesirable loss of protein structural integrity and techno‐functional performance in sensitive food formulations. Additionally, progress toward commercialization is limited by a lack of in vivo health effects studies and incomplete regulatory compliance for novel food ingredients.

Future research should prioritize structured strategies for technology transfer between academia and industry to efficiently scale up process innovations within CE models. In particular, pilot‐scale and continuous validation of integrated fractionation pathways should be prioritized, including process robustness, mass and energy balances, and the recovery of water and solvents. Research should expand the use of compositionally enhanced, functionally designed fractions in food formulations and further investigate F and enzymatic techniques to enhance the accessibility and nutritional value of dry‐enriched ingredients. The main challenge is to maintain ingredient functionality and purity while reducing resource consumption and process complexity; therefore, research should optimize fractionation conditions according to both recovery performance and techno‐functional properties rather than emphasizing only the extraction yield.

Alongside potential cereal fractionation studies, these must consider energy use, water and solvent use, and GHG emissions to enable significant process‐based assessments throughout the processes. The integration of LCA and TEA at an early stage of process development can help identify environmental hotspots, economic constraints, and trade‐offs between recovery yield, product purity, process complexity, and sustainability. More focus is required on the relationship between processing history and techno‐functional performance in complex food matrices. Thus, the combination of TEA and LCA with real‐time analytical monitoring and digital process modeling can help to mitigate scale‐up risks and foster industrial adoption. A subsequent priority is to strengthen in vivo validation, safety assessment, stability evaluation, and regulatory consideration of recovered ingredients to support their transition from experimental products to food applications. Process control and decision‐making under variable feedstock compositions can be supported through real‐time monitoring, process modeling, and multi‐criteria optimization. Figure 11 depicts an industrial translation roadmap that integrates technological, environmental, economic, food‐application, safety, and regulatory aspects. Therefore, a practical research pathway should progress from laboratory‐scale process optimization to integrated process development, pilot‐scale validation, LCA and TEA, food formulation and functional validation, safety and regulatory assessment, and finally industrial demonstration. Such coordinated research can help overcome the current technological, environmental, economic, and regulatory barriers to cereal biorefinery implementation.

**FIGURE 11:**
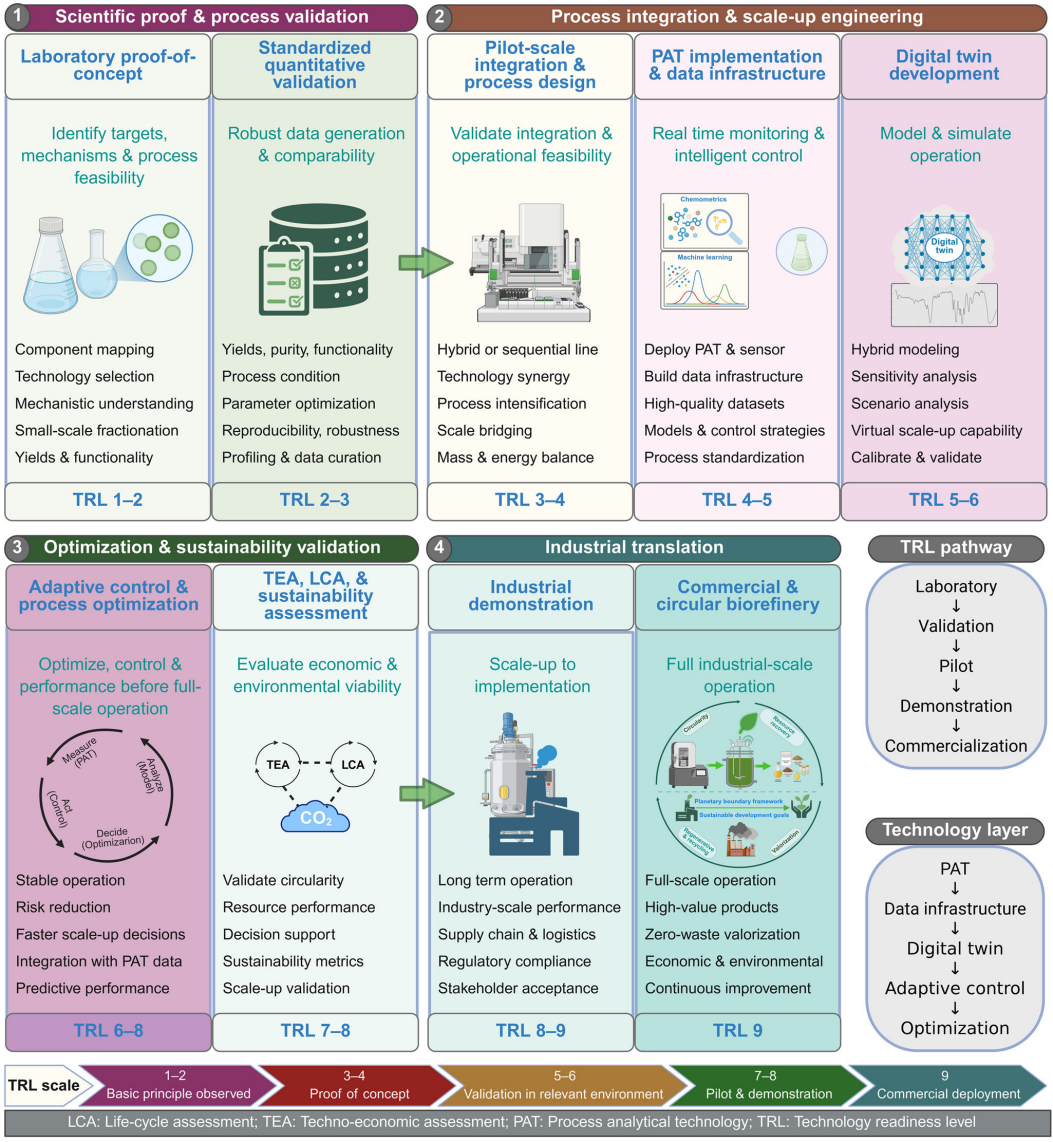
Industrial translation roadmap for cereal biorefineries.

## 8 Conclusion

The state‐of‐the‐art cereal fractionation is structurally transitioning from a linear produce‐use‐dispose model toward circular biorefinery systems designed for whole‐grain valorization. However, current processing focuses on refined flour production, with a preference for dry or wet milling, whereas nutrient‐dense germ and bran fractions are typically downgraded to low‐value animal feed or fertilizer. This practice can limit the recovery of food components, bioactives, and micronutrients, thereby reducing nutritional value and resource efficiency. Zero‐waste food biorefinery approaches can offer a unique perspective by treating grains and side streams as multifunctional feedstocks for the recovery of high‐value biochemicals and FIs, whereas residual fractions may be further valorized in food‐compatible or safe downstream applications. The sequential extraction of lipids, phytochemicals, proteins, and dietary fibers can provide significant functional and sustainability advantages by improving inclusive material efficiency, broadening product varieties, and promoting economic resilience compared with traditional milling operations. The choice of methods can be based on the fraction's intended application, the high‐throughput efficiency of dry mechanical separation, and the higher purity achievable through wet‐chemical or enzymatic fractionation. Dry fractionation methods, such as air classification and electrostatic separation, are considered the most prominent due to their minimal water and energy requirements and their ability to enrich protein and soluble fiber fractions without chemical solvents, compared with wet extraction. Consequently, recent process advances have focused on improving the recovery of proteins, starch, fiber, and bioactive compounds through moderate fractionation to preserve the native biopolymer functionality previously degraded by harsh thermal or chemical conditions. This preservation is important for developing the next generation of functional foods, in which the bioactivity of a phenolic compound or the gelling properties of a plant protein determine the final product's quality. The commercial feasibility of cereal derivatives increases as recovery rates improve, enabling producers to replace synthetic additives with clean‐label, grain‐based alternatives while maintaining excellent nutritional and sensory quality. The integration of nonthermal treatment, enzymatic procedures, membrane separation, and SFE demonstrates an advancement in precision‐based techniques that enable complex molecular‐level modification. Therefore, a hybrid strategy, such as cascading wet and dry fractionation with auxiliary technologies (e.g., US and PEF), can overcome the selectivity and purity challenges of linear dry processes while improving extraction efficiency. The study indicates that the transition toward industrial cereal biorefineries depends not only on improving individual extraction yields but also on integrating multiple fractionation operations to recover diverse FIs while maintaining product functionality, resource efficiency, and process sustainability. The integration of dry and wet fractionation, advanced nonthermal and bioprocess technologies, process monitoring, LCA, TEA, and digital process modeling provides a basis for developing more integrated and circular cereal processing systems. Optimal valorization of whole‐grain cereals and side streams can reduce food system waste, enhance resource efficiency, and provide resilient, low‐impact alternatives to non‐renewable inputs amid increasing climate and resource constraints. The industrial translation of these approaches will ultimately require coordinated optimization of recovery performance, functional quality, environmental impact, economic feasibility, safety, and regulatory suitability across the complete biorefinery system.

## Author Contributions

**Rahul Biswas**: conceptualization, data curation, methodology, visualization, writing – original draft, formal analysis, writing – review and editing. **Marjo Pöysä**: conceptualization, supervision, writing – review and editing. **Samson Adeoye Oyeyinka**: conceptualization, methodology, supervision, writing – review and editing. **Abayneh Getachew Demesa**: data curation, methodology, writing – review and editing. **Savindi Wanninayake**: data curation, methodology, writing – review and editing. **Samuel Perez‐Vega**: conceptualization, supervision, writing – review and editing, project administration.

## Funding

This study was funded by Regional Council of Kymenlaakso (Alueiden kestävän kasvun ja elinvoiman tukeminen, AKKE funding), KooRuoKo Project (Korkean osaamisen ja kestävän ruokasysteemin Kouvola), grant number: D/40/10.00.02/2024; Lappeenranta–Lahti University of Technology (LUT) (open access funding).

## Conflicts of Interest

The authors declare no conflicts of interest.

## Data Availability

Data will be made available on request.
